# DMS-informed secondary structure modeling of Epstein–Barr Virus LMP-1 pre-mRNA defines novel elements spanning introns

**DOI:** 10.1371/journal.pone.0345208

**Published:** 2026-07-02

**Authors:** Taylor O. Eich, Evelyn C. Coppenbarger, Abdelraouf O. Dapour, Walter N. Moss

**Affiliations:** 1 Department of Biochemistry, Biophysics and Molecular Biology, Iowa State University, Ames, Iowa, United States of America; 2 Bioinformatics and Computational Biology Program, Iowa State University, Ames, Iowa, United States of America; Guangdong Medical University, CHINA

## Abstract

The Epstein–Barr virus (EBV) infects over 95% of adults, establishing lifelong latency and contributing to the development of various malignancies, including Burkitt lymphoma and nasopharyngeal carcinoma. However, the RNA structures regulating the splicing of the critical EBV gene, latent membrane protein 1 (*LMP1*), remain uncharacterized. To identify these regulatory elements, we applied spliceosome inhibition with RNA probing and sequencing (SIRP-seq) to the BJAB-B1 cell line. By utilizing the spliceosome inhibitor pladienolide B, we enriched pre-mRNA species, enabling the detection of structural features within both the full-length pre-mRNA (*LMP1-FL*) and an alternatively spliced isoform retaining intron 2 (*LMP1-AS*). The resulting chemical probing datasets informed the RNA folding algorithms RNAfold and ScanFold to generate the first high-resolution secondary structure models for the *LMP1* pre-mRNA, encompassing both exonic and intronic regions. Our results identify 11 novel, thermodynamically stable RNA structures, with several key elements positioned near splice junctions. Notably, three structures (Structures 8, 9, and 10) were identified near the 3′ splice site of intron 2, appearing in alternative conformations that may influence splicing accessibility. Furthermore, these structures map to regions containing disease-relevant mutations associated with patient survival in Burkitt lymphoma. This structural framework provides new insights into how *LMP1* splicing may be regulated by RNA structure and identifies potential novel therapeutic targets for mitigating EBV-associated diseases.

## Introduction

The Epstein–Barr virus (EBV) has a pervasive impact on human health, infecting over 95% of the global population. EBV infection is associated with over 200000 new cases of cancer annually, including Burkitt lymphoma [1], nasopharyngeal carcinoma, Hodgkin lymphoma, and diffuse large B-cell lymphoma [2]. There is an increased risk of developing EBV-associated lymphomas in the immunocompromised, including post-transplant patients and those with HIV [3]. EBV is also linked to the development of several autoimmune disorders [4–7], including multiple sclerosis [8]. Its ubiquitous prevalence stems from its adaptation to multiple cellular environments and evasion of the host immune system [9], underscoring the need to understand EBV pathogenesis to develop therapeutics for treating and preventing EBV-associated diseases. EBV spreads primarily via the shedding of virions into saliva during the lytic cycle following reactivation from latency. After primary infection, the virus establishes lifelong latency as a circular episome within the host B cell nucleus, hijacking host cell machinery to replicate and transcribe its latent genes. Viral latency patterns depend on the differentiation stage of the host B cell. Latency III is the most permissive and all of the latent genes are expressed, including the EBV-nuclear antigens (*EBNA-LP*, *−1*, *−2*, and *-3A*, *-3B*, and *-3C*), latent membrane proteins (*LMP1*, *LMP2A*, and *-2B*), EBV-encoded RNAs (*EBER1* and *EBER2*), and BART miRNAs [10]. EBV contains a double-stranded DNA genome of ∼172 kb and encodes over 80 genes, but significant sequence divergence within the *EBNA2* and *EBNA3* genes distinguishes EBV into two strains (type 1 or type 2) that differ in lytic activity and transformation efficiency [11]. During latency, *EBNA2* activity drives metabolic reprogramming with cellular c-MYC (MYC) and LMP1. This cooperation upregulates glycolysis, lipid biosynthesis, and nucleotide synthesis pathways to support viral genome replication and maintain proliferative growth [12]. Cellular stress can activate B cell receptors and trigger lytic reactivation, which is required for transmission to new hosts. Immediate-early genes, *BZLF1* and *BRLF1*, initiate the switch from latent to lytic replication by activating downstream lytic genes [13], a process that may also enhance oncogenesis [14,15]. *LMP1* maintains cell growth and survival by acting as a functional mimic of the CD40 receptor to signal the activation of NF-κB pathways [16]. LMP1 is a driver of malignant transformation in B lymphocytes, where it appears during latency and lytic infection. The expression of *LMP1* can be differentially regulated by the multiple gene promoters encoded in the *EcoRI-Dhet* region of the EBV genome [17]. The full-length *LMP1* mRNA transcript is initiated from the ED-L1 promoter during latency, translating into a protein with a molecular weight of 62−65 kDa. The downstream ED-L1A promoter, located within intron 1, is upregulated during lytic cycle induction. The lytic *LMP1* (*lyLMP1*) mRNA transcript produces a 45-kDa protein from an initiation site at methionine 129, resulting in a truncated N-terminus [18]. Structurally, the full-length *LMP1* protein consists of a short N-terminal cytoplasmic domain, six transmembrane domains, and a cytoplasmic tail containing three C-terminal activating regions (CTARs) [19]. CTARs 1 and 2 possess critical transformation effector sites that induce B-cell immortalization by recruiting tumor necrosis-associated factors to activate the NF-κB pathways [20]. Interestingly, the lyLMP1 protein can negatively regulate LMP1-mediated carcinogenesis [21]. LMP1 has a rapid turn-over rate (2−3 hours), driven by serine/threonine phosphorylation, which initiates proteolytic cleavage, resulting in a 25 kDa C-terminal fragment (p25) that accumulates in the cytoplasm [19]. During early infection of primary B cells, MYC can repress the transcription of *LMP1* [22]. MYC is a master regulator of transcription capable of amplifying the transcription of genes involved in cell cycle progression to upregulate cell growth and proliferation [23]. Dysregulated *MYC* and *p53* inactivation are hallmarks of oncogenesis. In approximately 70−80% of Burkitt lymphoma cases, a chromosomal translocation event repositions the *MYC* gene under the control of the highly active immunoglobulin heavy-chain (*IgH*) promoter, where it becomes constitutively activated and drives cancer progression [24]. In normal B cells, overexpressed *MYC* activates apoptosis via p53-dependent pathways. However, *p53* is commonly mutated or deleted in Burkitt lymphoma cases, abrogating apoptotic signaling [25]. Furthermore, the viral protein EBNA1 competes with p53 for binding to the ubiquitin-specific protease USP7, protecting the infected cell from apoptosis by reducing the levels of p53 [26]. These interactions highlight how viral and host pathogenic factors cooperate to facilitate tumorigenesis. While multiple factors contribute to EBV-mediated oncogenesis, our study focuses specifically on *LMP1* to investigate how its pre-mRNA structures may be involved in regulating splicing. Splicing is a critical regulator of gene expression and is frequently dysregulated in cancer [27]. Alternative splicing expands the proteome by altering coding sequences, which can fine-tune function or generate pathologic isoforms. Viruses exploit host splicing machinery by modulating the expression or localization of splicing factors, including heterogeneous nuclear ribonucleoproteins (hnRNPs), serine-arginine rich (SR) proteins, and small nuclear ribonucleoproteins (snRNPs) [21]. For example, *EBNA1* is expressed in all EBV-associated malignancies, alters the alternative splicing of cancer-related host genes, and directly dysregulates the expression of splicing factors such as *Fox-2*, *hnRNPA1*, and *SF1* [28]. In a comprehensive analysis of the EBV transcriptome, a collection of new and existing alternatively spliced isoforms expressed under lytic reactivation were suggested to have a role in the lytic cycle [29]. Splicing modulation requires specific interactions with splicing regulatory elements (SREs) within RNA sequences that can either suppress or enhance splice site recognition. SRE interactions rely on RNA-RNA or RNA-protein interactions and may become sequestered or exposed as RNA structures assemble and disassemble. Here, we map and characterize high-resolution RNA structures that form near the *LMP1* splice junctions, offering a structural framework for understanding splicing regulation and uncovering potential vulnerabilities in EBV-associated malignancies. Previously, a transcriptome-wide structural analysis of EBV was completed using the EBV-infected lymphoma cell line (BJAB-B1) and presented thermodynamic characterizations for the latent mature mRNA transcripts and the type-2 genome [30]. In the transcriptome-wide structural analysis, there was a lack of coverage for the introns of *LMP1*. To deduce the structures within the pre-mRNAs that could be involved in *LMP1* splicing, we applied the spliceosome inhibition with RNA probing and sequencing (SIRP-seq) method to the BJAB-B1 cell line. SIRP-seq captures intronic RNAs by inhibiting the spliceosome, which leads to an accumulation of retained introns that can be chemically probed and targeted to acquire structural data for pre-mRNAs [31]. The *LMP1* pre-mRNA contains three exons separated by two introns. When the spliceosome inhibitor is applied, two isoforms were observed, the full-length (*LMP1-FL*) unspliced isoform containing both introns, and an alternatively spliced (*LMP1-AS*) isoform with only intron 2. The sequencing datasets collected for the *LMP1-FL* and *LMP1-AS* pre-mRNAs were integrated into RNAfold, ScanFold, and DRACO to generate the first secondary structure models for *LMP1* pre-mRNAs.

## Materials and methods

### ScanFold analysis

ScanFold is an RNA structure discovery pipeline that determines local structural stability and unusual sequence-ordered stability to assess potentially functional RNA secondary structures [32]. This is accomplished by weighing each base pair by its thermodynamic contributions to the order of structural stability within an RNA sequence, which is split into two steps: a scanning step and a folding step. During the scanning step, a 120-nucleotide window was used to analyze the sequence of interest, which was either full-length or alternatively spliced *LMP1* sequence. For each window, the program RNAfold from the Vienna RNA 2.0 package [33] is used to generate a native minimum free energy (MFE) and secondary structure for the sequence within the window. The $\Delta {\text{G}}^{\circ }$ value for each MFE structure is calculated from empirically measured thermodynamic energy parameters (the Turner rules) [34,35]. The sequence is then shuffled 100 times using mononucleotide shuffling, and the $\Delta {\text{G}}^{\circ }$ calculations are repeated for the shuffled sequences, generating a distribution of $\Delta {\text{G}}^{\circ }$ values. The average of the randomized $\Delta {\text{G}}^{\circ }$ values is calculated and used to determine the thermodynamic z-score. The z-score is calculated using the difference between the native $\Delta {\text{G}}^{\circ }$ value from the unshuffled sequence and the averaged $\Delta {\text{G}}^{\circ }$ value from the distribution of shuffled sequences before normalizing the difference by the standard deviation of the native sequence from the mean $\Delta {\text{G}}^{\circ }$ value of the distribution. Thus, the z-score reflects the number of standard deviations the native MFE structure is more or less stable than random and can be used as an estimate for unusual sequence-ordered stability in RNA structures. The z-score can be used as an initial filter for determining the functional propensity of a structure, as it may have been ordered by evolution of sequence to maintain thermodynamic stability. Next, the folding step analyzes all the associated z-scores to generate a consensus structure that represents each recurring nucleotide pairing across the low z-score analysis windows. The metrics output from the ScanFold analysis thus includes the MFE, ΔG z-score, p-value, and ensemble diversity. This last metric is derived from a partition function calculated (via RNAfold). A single nucleotide step was used for the sliding window analysis, and the folding temperature was set to 37°C. All secondary structures with a ΔG z-score below −1.0 were extracted for further analyses, revealing 11 structures for the *LMP1-FL* dataset and 12 structures for the *LMP1-AS* dataset. The ScanFold data is available in S1 Dataset. ScanFold structures were extracted for the merged 4-hour pladienolide B (4-hour PB merged) *LMP1-FL* and *LMP1-AS* datasets, which were used for annotating the pre-mRNA structure models.

### Cell culture and treatment

BJAB-B1 cells were initially derived from an EBV-negative diffuse B-cell lymphoma cell line immortalized with EBV-2 and express the latency III genes [36,37]. The BJAB-B1 cells (gift from the Joan Steitz Lab) were cultured at 37°C with 5% CO_2_ in RPMI (Life Technologies Corporation, New York, Gibco) media supplemented with 2 mM L-glutamine (Life Technologies Corporation, New York, Gibco), 1% penicillin–streptomycin (Life Technologies Corporation, New York, Gibco), 10 mM HEPES (Life Technologies Corporation, New York, Gibco), 1 mM sodium pyruvate (Life Technologies Corporation, New York, Gibco), and 10% FBS (Life Technologies Corporation, New York, Gibco). Cell density was measured using trypan blue staining and an automated cell counter. Approximately 10 million cells were plated into each well of a 6-well dish.

### Spliceosome inhibition

A 1 mM stock of pladienolide B (Cayman Chemical Company, Ann Arbor, Michigan) was first diluted into DMSO (VWR, Solon, Ohio), forming a 35 µM working solution. The working solution was added into RPMI media to generate a final concentration of 350 nM. Cells incubated with pladienolide B or DMSO (control) for 4 hours at 37°C with 5% CO2. Briefly, the predominant effects of pladienolide B inhibition include exon skipping and intron retention events, which can significantly impact other cellular processes and lead to reduced cell viability after extended incubation times [38]. However, pre-mRNAs can be captured using a shorter incubation time, limiting exposure to 4 hours. After incubation, the cells were pelleted by spinning at 200 x g for 3 minutes before subsequent chemical probing with DMS in a fume hood.

### DMS probing

RNA structure probing followed the DMS-MaPseq protocol [39,40]. A fresh 2% dimethyl sulfate (DMS) solution was prepared by diluting a concentrated stock of 99.9% DMS (MilliporeSigma, Saint Louis, Missouri) into a pre-warmed solution (37°C) of DPBS (Life Technologies Corporation, United Kingdom, Gibco) mixed with 25% ethanol. Cells were chemically probed for exactly 1 minute at room temperature by submerging the pellet in the 2% DMS solution. After the 1-minute incubation, the reaction was quenched twice with an equal volume of a pre-warmed dithiothreitol (DTT) (Gold Biotechnology, Saint Louis, Missouri) in DPBS as a quench solution, which contains a 5-times molar excess of DTT (1.1 M) to DMS (0.22 M). Each reaction was quenched twice before the next sample was probed. After the reactions were quenched, the samples were centrifuged at 200 x g for 3 minutes. The quenched solution was disposed and an aliquot of TRIzol reagent was used to dissolve the remaining cell pellet. The TRIzol samples were stored on ice for subsequent processing. The pladienolide B treatment and DMS chemical probing steps are outlined in S1 Protocol.

### Isolation and purification of total RNA from TRIzol

The RNA was isolated and purified following the manufacturer protocol and materials provided in the Direct-zol RNA miniprep kit (Zymo Research, Irvine, California). TRIzol samples were mixed with equal volumes of ethanol before loading onto a spin column. The samples received a treatment with DNase I to remove genomic DNA. The samples were washed to remove the proteins and lipids before the RNA was eluted with 75 µl of sterile nuclease-free water. The concentration of the total RNA was measured using a NanoDrop 2000 Spectrophotometer (Thermo Scientific), and yields were recorded (619–1355 ng/µl) with acceptable purities (A260/A280 > 1.9).

### Selection of poly-adenylated RNAs from total RNA

For each sample, 600 ng of total RNA were processed with the poly-(A) purist-MAG purification kit (Invitrogen) to isolate poly-A RNAs from the total RNA pool. This step was performed to increase the specificity for poly-adenylated mRNA and pre-mRNAs during reverse transcription by removing the highly abundant ribosomal RNAs. The poly-A selected RNAs were quantified using the NanoDrop (12–41 ng/µl) and each sample contained pure RNA (A260/A280 > 2.0). 75 ng of each sample was used as input for the reverse transcription reactions.

### Reverse transcription and polymerase chain reaction

During the reverse transcription reaction, random mutations are introduced into the cDNA at sites where DMS methylated an unpaired nucleotide in the template RNA. DMS preferentially methylates the Watson-Crick faces of unpaired adenosine and cytosine nucleotides during the chemical probing step [40]. The accumulation of multiple mutations on a single cDNA molecule is possible due to the Induro® thermostable group II reverse transcriptase (New England Biolabs, Ipswich, Massachusetts). Mutational read-through provides additional structural information to be collected for each RNA molecule. The entire transcriptome can be accessed from the generated cDNA by utilizing random hexamer priming. Additionally, increased coverage of the poly-adenylated regions can be achieved by adding poly-dT primers. The cDNA samples were processed with a DNA clean and concentration kit (Zymo) following the manufacturer protocol and eluted with 15 µl of sterile nuclease-free water. The cDNA was quantified at the ISU DNA facility using a Qubit® 2.0 Fluorometer (27–49 ng). The concentration of template cDNA was normalized to 1.0 ng/µl. For each unique PCR reaction, a separate primer mix containing a 1:1 ratio of 5 µM forward and reverse primer was prepared (primers obtained from Integrated DNA Technologies, Coralville, Iowa). Then for each sample, 2.0 µl of template cDNA and 2.0 µl of the designated primer mix were combined into a separate sample tube and kept on ice. Next, the PCR master mix was prepared by combining 0.4 µl of 10 mM dNTPs, 4.0 µl of 5x Q5 Reaction Buffer (NEB), and 11.4 µl of nuclease-free water. Then, 0.2 µl of Q5 High-Fidelity DNA Polymerase (NEB) was added to the PCR master mix and gently mixed. Each 20 µl reaction was assembled by combining 16 µl of the PCR master mix with 4.0 µl of the sample-primer mix. The primer sequences and PCR stage settings used for amplification of cDNA are provided in S1 Table.

### Validation of intron retention

Three sets of gene-specific primers were designed to span across each splice junction of *LMP1* and cover 85% of the entire sequence (S1 Fig). The targeted region begins in the 3′ untranslated region (UTR) region and ends upstream of the truncated region in exon 3. Intron retention was confirmed during an initial screen using 35 PCR amplification cycles and the alternatively spliced product verified with the DNA 1000 chip for the Agilent 2100 Bioanalyzer (S1 Appendix). Following validation, the PCR reactions were repeated using only 30-cycles to reduce off-target amplification. The target products were isolated by preparing a gel with the SYBR-Safe DNA gel stain (Thermo Fischer Scientific, Carlsbad, California) and visualized on a gel doc EZ imager (Bio-Rad) using the blue light setting. Individual product bands were collected by excising the spliced (lower), alternatively spliced (middle), and full-length (upper) PCR target product bands from the gels on a blue light LED transilluminator (Invitrogen). Gel slices were processed with the Zymoclean™ gel DNA recovery kit (Zymo Research, Irvine, California) and quantified with the Qubit® fluorometer (28–486 ng) (S2 Appendix).

### Illumina DNA library preparation and sequencing on iSeq100

The Illumina DNA library preparation protocol was used to prepare libraries that were pooled for sequencing on the iSeq100 benchtop sequencer (Illumina). The library preparation protocol is separated into five parts and allows for multiplexing of up to nine individual libraries on a single-flow cell. The Illumina DNA prep kit uses a bead-linked transposome complex to tagment the DNA, which fragments and tags the DNA with adapter sequences and normalizes the distribution of DNA fragment sizes. Then, a limited number of PCR cycles are used to apply sequence specific index adapter sequences to the ends of the DNA fragments. Following amplification, the libraries are purified with the purification beads and quantified before a normalized amount of each library is added into the final pool for loading onto the flow cell. The concentrations of 16 individual libraries were determined with the Qubit® 2.0 Fluorometer using the DNA high sensitivity kit and the average fragment size of each library was determined using a DNA high sensitivity chip for the Agilent 2100 Bioanalyzer at the ISU DNA facility (S3 Appendix). The final concentration of the pooled libraries that were directly loaded onto the flow cell was 75 pM.

### Sequencing data analysis with RNAframework

The paired-end sequencing reads were collected and stored on the iSeq100 as FASTQ files, which were uploaded onto the Iowa State HPC cluster for data analysis. The quality of the forward and reverse reads was checked with the FASTQC module and Phred scores were consistently above 30, where a Phred score of 30 indicates 99.9% base calling accuracy during sequencing (S1 Dataset). FASTQ files were trimmed using SAMtools [41] and the Nextera DNA Flex adapter sequence “CTGTCTCTTATACACATCT” before further processing with RNAframework [42]. The RNAframework pipeline (Version: 2.9.3) was utilized to analyze the trimmed FASTQ files with the rf-map, rf-count, rf-norm, DRACO, and rf-fold modules. Datasets for the *LMP1* unspliced, alternatively spliced, and spliced isoforms were generated by mapping the individually prepared libraries to their respective fasta files (S1 Dataset). Following the mapping step with rf-map, the binary alignment map (BAM) files from replicate experiments were merged into a single BAM file using SAMtools. Since the adenosine and cytosine nucleotide mutations were the most informative for DMS chemical probing, they were selected for during the rf-count step. The resulting RNA count (RC) files were normalized with the rf-norm module using the Zubradt method. First the raw signal at each position is calculated by dividing the per-base mutational frequency by its coverage, then taking the top 10% of raw signals and performing a 2–8% normalization. The 2–8% normalization uses the top 10% of the raw signal values calculated, eliminates the upper 2%, and divides the raw signal at every position by the average of the remaining 8%. The normalized signal values are output as an extensible markup language (XML) file, which can be directly used for structure prediction with the rf-fold module. Shannon entropies were also calculated using the RNAframework rf-fold module to identify regions that were well-defined or disordered across a 600-nt window. The normalized values were also converted into separate reactivity (REACT) files for the ScanFold algorithm. A Pearson Correlation was used to show that the nucleotide z-scores, ensemble diversity values, and the reactivity values had a high correlation between the *LMP1-FL* and *LMP1-AS* datasets (S2 Table).

### *LMP1* and *LMP1-AS* read coverage validation

Raw sequencing reads from the previously published transcriptome-wide analysis of EBV in BJAB-B1 [30] were reanalyzed using the Structure-seq2 pipeline [43]. The dataset (BioProject accession PRJNA865760) was downloaded from the NCBI Sequence Read Archive using the SRA Toolkit (Version 3.4.1) to inspect coverage across the intronic regions in *LMP1*. Raw reads were evaluated with FastQC, trimmed using conservative fastp quality-filtering parameters, and summarized with MultiQC. Trimmed reads were then merged by probing condition into DMS-positive and DMS-negative groups. Separate Bowtie2 indexes were generated for the *LMP1* and *LMP1-AS* transcript references, and each condition was aligned independently to both references using Bowtie2 in very-sensitive local mode. The resulting alignments were converted to sorted and indexed BAM files using SAMtools, and mapping summaries were generated using flagstat and idxstats. Final BAM files were visualized in Integrative Genomics Viewer [44] to assess read coverage across the introns in *LMP1-FL* and *LMP1-AS* pre-mRNAs (S2 Fig).

### Deconvoluting RNA conformations with the DRACO algorithm

DRACO is a computational algorithm designed to identify multiple distinct RNA structural conformations from mutational profiling experiments [45]. This was accomplished using RNAframework by generating the mutational map (MM) files during the rf-count step. The DRACO algorithm was set to run with default parameters and uses a sliding window analysis to analyze a reference sequence. For each window, spectral clustering is performed to determine the number of conformations present. The number of conformations is equivalent to the number of informative eigengaps when compared to the null model that was built by permutating the original data matrix. After determining the number of conformations in each window, each base within the conformation is weighed based on its affinity to each conformation during fuzzy clustering and a stoichiometric abundance for each conformation is calculated. Once the optimal solution is defined, the mapped sequencing reads are assigned to a single conformation. Lastly, consecutive windows with the same number of conformations are merged into larger window sets. DRACO outputs the conformational reactivity data as a JSON file, which can be converted into an RC file with the rf-json2rc module. The resulting RC files are generated with a text file containing the extracted conformational regions and the stoichiometric abundances associated with each conformation in the region. The DRACO regions for the 4-hour PB merged *LMP1-FL* and *LMP1-AS* datasets were highlighted (S1 and S2 Files), and regions with alternative structures near the splice sites were characterized further.

### Folding *LMP1* RNAs with RNAfold using the DMS reactivities and modeling with VARNA

The secondary structure models for the *LMP1-FL* and *LMP1-AS* pre-mRNAs of *LMP1* were predicted with RNAfold using the DMS reactivities obtained from the pladienolide B treated samples. A maximum folding distance was set to 600 bp, since over 99% of base pairs that occur within ribosomal RNAs are found within that distance [46]. The partition function was calculated to get the base pair probabilities for each i and j pair that formed within constrained folding distance. Base pair probabilities were calculated using the partition function [47], and then were colored based on their frequency. The VARNA software [48] was used to model the RNAfold models and annotate the exons. The REACT files were converted into VARNA text files before mapping the reactivity values to RNA structure models as a color map, by setting all reactivities on a scale from 0.0 to 1.0, where a reactivity value of 1.0 is considered reactive.

### Conservation of sequence within variants isolated from tumors of EBV infected patients

The full-length *LMP1* sequences were extracted from the EBV genomic sequences that were downloaded from the BV-BRC database [49], which were collected from the tumors of cancer patients infected with EBV. Incomplete sequences were filtered out and manually trimmed before using MAFFT [50] to align the sequences to the *LMP1* reference sequence for the EBV-2 genome (NC_009334.1:170575–169188). The sequence alignment was utilized for analyzing the sequences where ScanFold structures were identified (S4 File). The WebLogo tool [51] was utilized to create sequence logos to illustrate the conservation of nucleotides within each ScanFold structure (S3 Fig). The aligned sequences were also used for RNAalifold [52,53], which provided a secondary structure model that highlighted structures that formed within highly conserved sequences (S5 File) within the alignment and resulted in the identification of two conserved structure regions in the pre-mRNA structure models at positions (705–720) and (1032–1074).

## Results and discussion

### Identification of an alternatively spliced *LMP1* isoform

RT-PCR confirmed that pladienolide B inhibition induced intron retention in *LMP1*. Gel electrophoresis shows intron retention accumulated as incubation times increased, with upper (unspliced) and lower (spliced) bands appearing in each of the treated time points. When simultaneously targeting multiple introns, a third intermediate band was revealed, which represented an alternatively spliced isoform for *LMP1*. When mapping the reads from the alternatively spliced libraries to the full-length *LMP1* transcript (S4 Fig A), the read coverage across the intron 1 splice junctions was significantly reduced compared to intron 2 (S4 Fig B). Some reads mapped to the sequence of intron 1, but the read depths were significantly reduced compared to the *LMP1-FL* dataset (S4 Fig C). The alternatively spliced libraries were remapped to a reference sequence with intron 1 removed, and the *LMP1-AS* datasets were created and processed alongside the *LMP1-FL* datasets.

### Data analysis of the *LMP1-FL* and *LMP1-AS* isoforms

**Fig 1:**
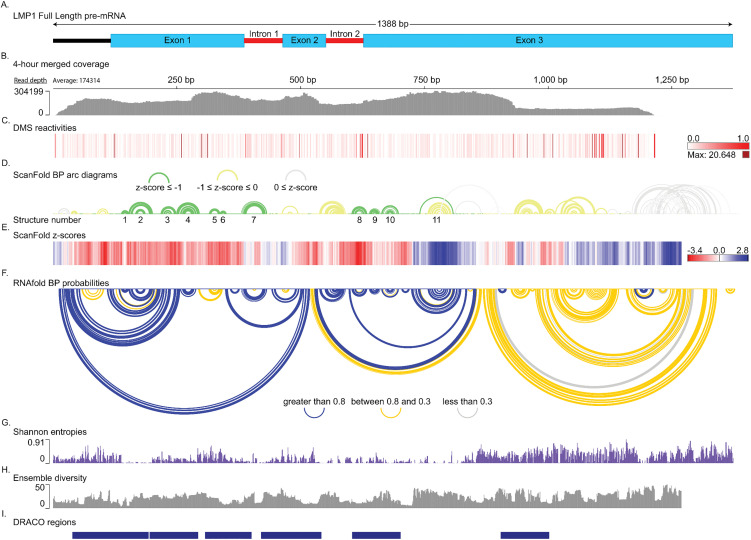
Data analysis overview for the full length *LMP1* isoform. **(A)** The cartoon for the *LMP1-FL* isoform is shown with a scale bar at the top. **(B)** The sequencing read coverage for the merged data generated from the 4-hour incubation with pladienolide B. The average and maximum read depth are shown on the left, with a scale for nucleotide position at the top. **(C)** The DMS reactivities obtained from RNAframework are aligned to their corresponding positions to the *LMP1-FL* transcript, and the intensities are given as a split range that has values between 0.0 and 1.0 increase from white to red, and values greater than 1.0 approach a darker shade of red set to the maximum reactivity value (20.648). **(D)** The ScanFold base pair (BP) arc diagrams are shown, which are colored according to their calculated z-scores (grey: value greater than 0, yellow: value between 0 and −1, and green: value between −1 and −2). **(E)** The individual per-nucleotide z-scores are shown by setting their values to a split color scale. The negative z-scores approach red as they near the lowest z-score (−3.4), z-scores at 0.0 are white, and positive z-scores turn a darker blue as they approach the highest z-score (2.8). **(F)** The base pair probabilities are colored, where the base pair probabilities above 80% are blue, pairings between 80% and 30% are yellow, and all pairings below 30% are grey. **(G)** The Shannon entropies are range from 0 to 0.91, where a lower Shannon entropy indicates more defined pairings compared to higher values that indicate disorder and less defined pairing. **(H)** The ensemble diversity is shown with a range from 0 to 50 alternative structures within the ensemble. **(I)** The DRACO regions that suggest alternative conformation profiles based on the DMS reactivities that clustered within its windowed analysis are shown as dark blue bars.

**Fig 2:**
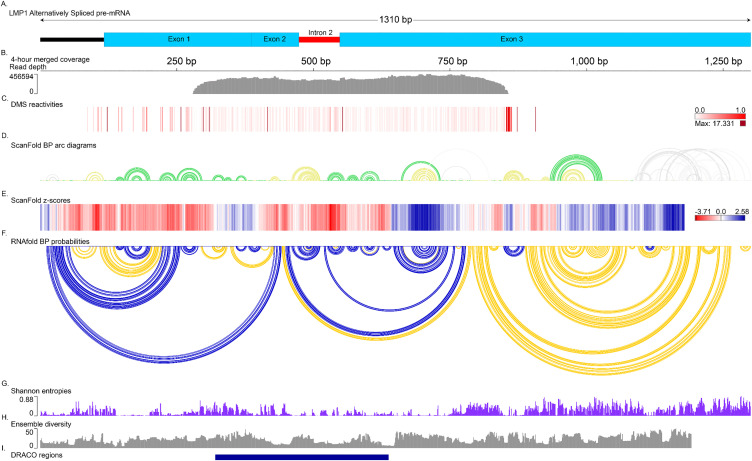
Data analysis overview for the alternatively spliced *LMP1* isoform. **(A)** The cartoon for the *LMP1-AS* isoform is shown with a scale bar at the top. **(B)** The sequencing read coverage for the merged data generated from the 4-hour incubation with pladienolide B. **(C)** The DMS reactivities obtained from RNAframework are aligned to the *LMP1-AS* transcript, and the intensities are given as a split range that has values between 0.0 and 1.0 increase from white to red, and values greater than 1.0 approach a darker shade of red set to the maximum reactivity value (17.331). **(D)** The ScanFold base pair (BP) arc diagrams are shown, which are colored according to their calculated z-scores (grey: value greater than 0, yellow: value between 0 and −1, and green: value between −1 and −2). **(E)** The individual per-nucleotide z-scores are shown by setting their values to a split color scale. The negative z-scores approach red as they near the lowest z-score (−3.71), z-scores at 0.0 are white, and positive z-scores turn a darker blue as they approach the highest z-score (2.58). **(F)** The base pair probabilities are colored, where the base pair probabilities above 80% are blue, pairings between 80% and 30% are yellow, and all pairings below 30% are grey. **(G)** The Shannon entropies range from 0 to 0.88, where a lower Shannon entropy indicates more defined pairings compared to higher values that indicate disorder and less defined pairing. **(H)** The ensemble diversity is shown with a range from 0 to 50 alternative structures within the ensemble. **(I)** The DRACO regions that suggest alternative conformation profiles based on the DMS reactivities that clustered within its windowed analysis are shown as a dark blue bar.

A total of 13 DMS reactivity datasets were generated from the individual sequencing libraries for the 0-hour, 2-hour, and 4-hour samples, but the complete analysis was limited to only the 4-hour PB merged *LMP1-FL* and *LMP1-AS* datasets because intron retention was more consistent. The 4-hour PB merged datasets were used to inform secondary structure predictions for the *LMP1-FL* and *LMP1-AS* sequences. The data collected for the *LMP1-FL* (Fig 1) and *LMP1-AS* (Fig 2) datasets were illustrated to give an overview of the structural features and metrics produced by ScanFold and RNAfold when informed with the DMS reactivity values.

Although the transcriptome-wide strategy offers a broad insight into the structural architecture of mature viral mRNA transcripts, they frequently lack sufficient read depth to resolve low-abundance intronic pre-mRNA species, as evidenced by the sparse intronic coverage observed in baseline transcriptomic datasets (S2 Fig). When comparing the read depths obtained in the 4-hour merged datasets, an average read depth of 174314 reads was obtained for the *LMP1-FL* transcript (Fig 1B), and an average read depth of 153887 reads was achieved for the *LMP1-AS* transcript (Fig 2B). This targeted, deep-sequencing strategy provides the high-resolution mutational profiling data required to confidently model intronic secondary structures and track alternative conformational shifts at critical regulatory junctions. In the transcriptome wide analysis, average read depths were lower. Averages of 746 and 789 reads from the DMS+ datasets mapped to the *LMP1-FL* and *LMP1-AS* transcripts, respectively (S2 Figs C and F). The targeted strategy produced significant increases to the read depths within intronic regions, where the average read depths for intron 1 (192023 reads) and intron 2 (141359 reads) were obtained in the *LMP1-FL* 4-hour merged dataset. In the *LMP1-AS* dataset, intron 2 had an average read depth of 321408, offering higher number of reads to analyze RNA structures formed within the context of intron 2. In contrast, the transcriptomic dataset resulted in average read depths of 24 (DMS-, *LMP1-FL*) and 29 (DMS + , *LMP1-FL*) for intron 1, 21 (DMS-, *LMP1-FL*) and 19 (DMS + , *LMP1-FL*) for intron 2, and 19 reads for intron 2 (DMS+ and DMS-, *LMP1-AS*).

### *LMP1-FL* isoform data analysis

Fig 1A shows the *LMP1-FL* pre-mRNA cartoon, which is 1388 bp and starts upstream from exon 1 and ends at the 3′end of exon 3. Consistent read coverage was obtained for the targeted portions of the *LMP1-FL* transcript, where an average depth of 174314 reads and a maximum depth of 304199 was achieved (Fig 1B). The DMS reactivities are shown in Fig 1C, where the highest reactivity value was 20.648. The ScanFold base pair (BP) arc diagrams in Fig 1D show all the ScanFold structures that contained MFE z-scores that range from 0.0 to −2.0. The individual per-nucleotide z-scores are shown in Fig 1E, which had a range from −3.4 to 2.8. The RNAfold base pair probabilities are colored in Fig 1F, where high base pair probabilities and low Shannon entropy values (Fig 1G) indicate well-defined folding. Similarly, the ensemble diversity (Fig 1H) suggests the diversity of the ensemble of potential structures, with higher numbers indicating more potential conformations or a lack of a defined structure. The DRACO regions are shown (Fig 1I), which may indicate there are alternative conformations that can form near the 3′ splice sites, which is supported by the increased ensemble diversity at the 3′ splice junctions. Of the nine DRACO regions identified in the *LMP1-FL* dataset, two regions with alternative structures were extracted near splice junctions. Both regions were identified at 3′ splice junctions. The conformations found within the region spanning the 3′SS of intron 1 is shown in S5 Fig. The conformations spanning the 3′SS of intron 2 (S6 Fig) overlapped with ScanFold Structures 9 and 10.

### *LMP1-AS* isoform data analysis

The *LMP1-AS* isoform is shorter (1310 bp) than the *LMP1-FL* isoform. While both transcripts begin and end at the same respective nucleotides of the gene locus, removing the sequence for intron 1 shifts the nucleotide positions downstream of the exon 1 splice junction in the model for the *LMP1-AS* isoform (Fig 2A). The read coverage was consistent across the exon-exon and exon-intron junctions, with an average depth of 153887 and a maximum depth of 458594 (Fig 2B). The highest reactivity value obtained in the *LMP1-AS* isoform was 17.331 (Fig 2C). ScanFold BP arc diagrams for the *LMP1-AS* dataset are shown in Fig 2D. The per-nucleotide z-scores were lower when intron 1 was absent from the transcript, and the range shifted from −3.7 to 2.6. By comparing the z-scores upstream from the exon 2 splice junction in Fig 1E and the z-scores upstream from the exon 2 splice junction in Fig 2E, the z-scores surrounding the splice junction are lower when intron 1 is absent. The base pair probabilities of the subdomain between exon 1 and exon 2 are also lower when the intron is absent (Fig 2F). The Shannon entropies are very similar to the *LMP1-FL* model, with reduced entropies appearing near the 3′end of exon 3, upstream from Structure 12 (Fig 2G). When intron 1 is removed from the sequence, the Shannon entropies increase near the modified region, and moderate to high ensemble diversities are predicted (Fig 2H). Of the five DRACO regions extracted in the *LMP1-AS* dataset (Fig 2I), one region is highlighted at the 3′ splice junction of intron 2 (S7 Fig) and overlapped with ScanFold Structures 9 and 10.

### RNA secondary structure modeling for the *LMP1-FL* and *LMP1-AS* isoforms

**Fig 3:**
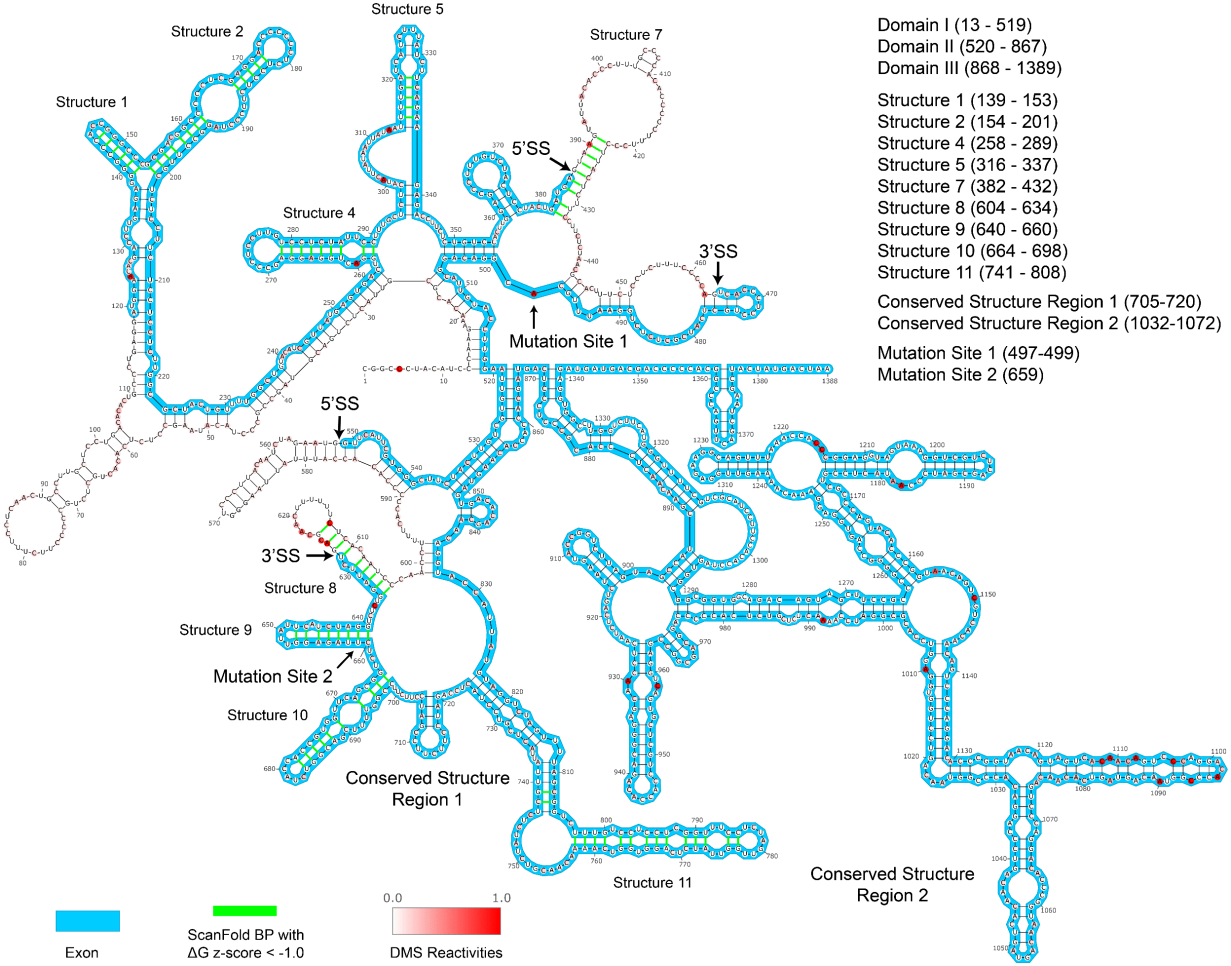
Secondary structure model for *LMP1* full length pre-mRNA. The model includes highlighted portions of exon 1 and exon 2 (light blue), the splice sites marked with arrows, and base pairs co-predicted with ScanFold −1 z-score structures (green). Normalized DMS reactivities from the merged 4-hour datasets are presented on a scale from 0.0 to 1.0, the most reactive bases were set to a maximum value of 1.0. The start and end coordinates for the major structural domains, overlapping ScanFold structures, conserved structure regions, and discussed mutation sites are listed.

**Fig 4:**
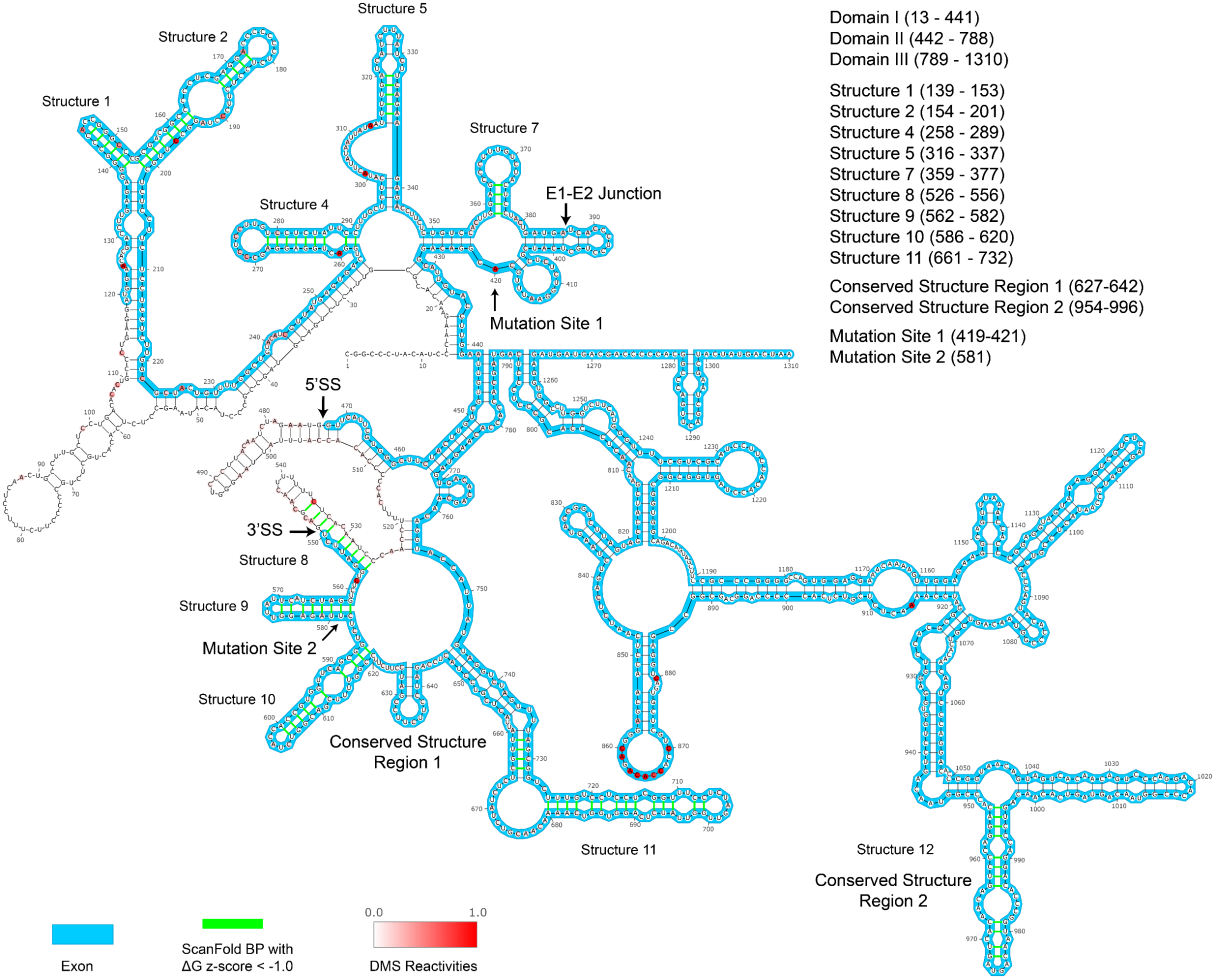
Secondary structure model for *LMP1* alternatively spliced pre-mRNA. The model includes highlighted portions of exon 1 and exon 2 (light blue), the splice sites marked with arrows, and base pairs co-predicted with ScanFold −1 z-score structures (green). Normalized DMS reactivities from the merged 4-hour datasets are presented on a scale from 0.0 to 1.0, the most reactive bases were set to a maximum value of 1.0. The start and end coordinates for the major structural domains, overlapping ScanFold structures, conserved structure regions, and discussed mutation sites are listed.

The secondary structures for the *LMP1-FL* and *LMP1-AS* isoforms were very similar. Each model contained three major structural domains with nine overlapping ScanFold structures. Base pairs shared between the extracted ScanFold structures and the RNAfold models were annotated to highlight RNA structures with potential for function. In both isoforms, multibranched structural domains form near the splice sites and single-stranded loops separate the 5′SS and 3′SS. The secondary structure for the *LMP1-FL* isoform is shown in Fig 3 and the structure for the *LMP1-AS* isoform is shown in Fig 4.

In Domain I (Figs 3 and 4), the main stem forms between the base pairs of the 3′UTR and exon 2. The main stem leads to a multibranch loop with four separate branches. The first branch has a stem that forms through base pairing between the 3′UTR and exon 1. The first branch splits into sub-branches, where the first sub-branch is made from a structure with multiple loops, formed from the 3′UTR sequence. Upstream is the start codon of exon 1, and the second and third sub-branches, which form using the sequence of exon 1. These sub-branches are also ScanFold structures (Figs 3 and 4, Domain I, Structures 1 and 2). Following the first branch of Domain I, the second branch and third branches form within the multibranch loop. The stem of the third sub-branch contains a 16-nucleotide bulge with an AU-rich sequence (302−314) and reactive bases and separates Structures 4 and 5 (Figs 3 and 4, Domain I, Structures 4 and 5). The AU-rich sequence (5′-UUAUAAUUAUAA-3′) is complementary to itself. The fourth branch of Domain I forms a stem between the sequences of exon 1 and exon 2. This stem leads to a second multibranch loop with three sub-branches. The first sub-branch is a short, four-paired stem with an 11-nucleotide loop, which is considered a ScanFold structure in the *LMP1-AS* model (Fig 4, Domain I, Structure 7). The second sub-branch forms between the sequences of exon 1 and intron 1 and forms ScanFold Structure 7 (Fig 3, Domain I, Structure 7). In Fig 3, Structure 7 contains reactive bases near the 3′SS of intron 1 and two of the four single-stranded regions within the intron, which form a 12-nucleotide internal loop that is separated by two-pairs and a tri-loop. Intron 1 contains a ten-nucleotide loop within the multibranch structure that separates the two splice sites. In the third sub-branch, there is an 11-nucleotide internal loop with reactive bases near the 3′ splice site of intron 1. At the 3′ splice site of intron 1 (Fig 3, Domain I, 3′SS), a short stem forms between the intron 1 and exon 2 sequences with two intron-exon pairs, a single nucleotide bulge, and two exon-exon base pairs. When the intron-exon junction gets spliced, the two intron-exon base pairs may dissociate to accommodate the incoming splicing machinery and the incoming exon 2. The sequence of exon 2 has sequence complementarity to the incoming exon 1 sequence, and RNA structures within the multibranch loop are maintained after splicing. The second and third sub-branches form a new sub-branch as the sequences of exon 1 and exon 2 are paired, which can be shown in the *LMP1-AS* model (Fig 4, Domain I, E1-E2 Junction). A mutation site is annotated at the end of the multibranch loop (Figs 3 and 4, Domain I, Mutation Site 1), upstream from the remaining sequence that forms the stem of the fourth branch and the main stem. In Domain II (Figs 3 and 4), the main stem is formed by exon 2 and exon 3. The main stem leads to a multibranch loop with three branches. The first branch contains the 3′SS of intron 2, which is formed within a hairpin that shares base pairs between exon 2 and intron 2. The sequence of intron 2 contains a pyrimidine-rich loop that connects the first and second branches. The second branch contains four base pairs shared between intron 2 and exon 3, which expands into a second multi-branch loop with five sub-branches. The first three sub-branches contain ScanFold structures (Figs 3 and 4, Domain II, Structures 8, 9, and 10). The 3′SS was predicted near the hairpin loop of Structure 8 and contained reactive nucleotides near the 3′SS of intron 2. The three ScanFold structures near the splice site could be involved in splicing, but further work is required to establish this. At the base of Structure 9, a second mutation site is annotated (Figs 3 and 4, Domain II, Mutation Site 2). There are two pyrimidine-rich loops after the third sub-branch and one is within a conserved region in the fourth sub-branch (Figs 3 and 4, Domain II, Conserved Structure Region 1). The fifth sub-branch contained ScanFold Structure 11 (Figs 3 and 4, Domain II, Structure 11). The backbone of the structures surrounding the splice junction includes a single-stranded loop in the second multibranch, a two-pair stem in the first multibranch, and the main stem of Domain II. The main stem of Domain III (Figs 3 and 4) leads to the first of two multibranch structures. In the first multibranch, there are four branches. The fourth branch leads to the second multibranch structure with two sub-branches. In the first sub-branch, there is a bifurcated loop that contains a conserved structure region (Figs 3 and 4, Domain III, Conserved Structure Region 2), which overlaps with ScanFold Structure 12 in the *LMP1-AS* model (Fig 4, Domain III, ScanFold Structure 12). The downstream structure of the bifurcated loop was highly reactive to DMS. The remaining sequence contains some reactive bases, but there is also reduced coverage in the downstream regions closer to the 3′end of exon 3, which result in structural differences in the *LMP1-AS* model (Fig 4, Domain III).

### DRACO identified alternative conformations near the 3′ splice sites

The DRACO program was used to identify potential alternative RNA structures that may form near the splice site junctions. Of the nine DRACO regions identified in the *LMP1-FL* dataset, two regions were extracted near splice site junctions. Three alternative conformations were extracted near the 3′ splice junctions of intron 1 (S5 Fig) and intron 2 (S6 Fig) in the *LMP1-FL* dataset. The c0 conformation spanning the intron 1 3′SS (S5 Fig A) had the lowest reactivity values upstream of the 3′SS, while the c2 conformation (S5 Fig B) and c3 conformation (S5 Fig C) had higher values. The reactivity profiles produced alternative structures that position the 3′SS at different locations within the structure of the RNA. When the splice site is positioned near the middle of the stem (S5 Figs A and B), the splice site may be less accessible, than when the 3′SS is positioned near the hairpin loop (S5 Fig C). Three alternative conformations were also extracted near the 3′ splice junctions of intron 2 (S6 Fig), containing ScanFold Structure 9 and Structure 10. The c0 conformation (S6 Fig A) shows Structure 10, while the c1 and c2 conformations (S6 Figs B and C) show Structure 9. When Structure 10 forms, the stem is shortened and the exonic sequence downstream from the 3′SS is positioned within a bulge (S6 Fig A). In the two conformations with Structure 9, the 3′SS is positioned near the base of the stem (S6 Figs B and C). In one conformation, the hairpin loop has a few high reactivity values near the hairpin loop, while the base of the stem has low values (S6 Fig B). In the other conformation, the reactivity values near the base of the stem are high, which could indicate the 3′SS is in a more accessible state (S6 Fig C). In the five DRACO regions identified for the *LMP1-AS* dataset, and one region contained two alternative conformations that formed at the 3′SS of intron 2 (S7 Fig). Both conformations position the 3′SS upstream of a branched loop structure with high reactivities near the splice junction. In the c0 conformation (S7 Fig A), Structure 9 is predicted without Structure 10 following an asymmetric loop with high reactivity values. In the c1 conformation (S7 Fig B), both Structure 9 and Structure 10 are present within the branched loop structure. The ability for Structure 9 to form in alternative RNA conformations may indicate how RNA structure engages in regulating splicing at the 3′SS of intron 2. One way alternative RNA structure could regulate splicing is by altering the accessibility of the 3′ SS for interactions with the splicing machinery.

### Mapping *LMP1* variants for disease relevant mutations to characterized RNA structures

Variable selection models identified *LMP1* variants as most relevant for predicting Burkitt Lymphoma patient survival rates [54]. Two mutations from a list of relevant mutations were selected (His101 and Met129), since they are located downstream from the 3′ splice junctions of intron 1 and intron 2. Using the aligned *LMP1* sequences in S5 File, the codon frequencies were calculated for both amino acid (AA) positions. Mutation site 1 (AA101) and mutation site 2 (AA129) are annotated in Figs 3 and 4. Position 101 had frequencies of His 298/340 (87.6%), Arg 21/340 (6.1%), Gln 18/340 (5.3%), Ser 1/340 (<1%), and Asn 2/340 (<1%), while position 129 had frequencies of Ile 313/340 (92.1%) and Met 27/340 (7.9%). We hypothesize these mutations impact RNA structures to alter splicing regulation in *LMP1*. Amino acid position 101 encodes histidine using the ‘CAC’ codon in the *LMP1* coding sequence (Figs 3 and 4, Mutation Site 1). When position 101 encodes the glutamine codon ‘CAA’, the mutation is significantly associated with the survival outcomes of African patients. The sequence of intron 1 contained U-rich single-stranded loops that could offer base pairing partners for this mutation (Fig 3). Mutation site 1 can also be observed in the alternative conformations (S5 Fig, positions 76–78), where a base pair could be made if the C > A mutation occurs in the alternative conformations with increased 3′SS accessibility (S5 Fig C). The sequence changes reflecting the other mutations were not reported to have effects on survival outcomes, but a mutation at the first position of the codon could alter the base pairing at the base of the third sub-branch of Domain I (Fig 3, Mutation Site 1). When position 129 reflects the Ile codon, the ‘AUU’ sequence is maintained in Structure 9 (Fig 3, Structure 9) by allowing an additional base pair, which may play a role in stabilizing the structure during splicing. When mapping the position of the G > U mutation to the alternative conformations (S6 Figs A, B, and C, position 54), the presence of a G-nucleotide reduces base pairing in Structure 9. If Structure 9 is absent, the position of mutation site 2 is within a single-stranded region between the 3′SS and Structure 10 (S6 Fig A). However, in the *LMP1-AS* dataset, when Structure 9 is absent, the mutation site is positioned within a tri-loop of a hairpin upstream from Structure 10 (S7 Fig A, position 45).

### Limitations to SIRP-seq

Chemical probing datasets aim to improve the accuracy of RNA folding algorithms by providing information of which nucleotides are likely to be unpaired. While RNA structure prediction is a powerful tool, RNA secondary structure models often provide a limited representation of the true state of molecules. It is critical to recognize that RNA structures are sensitive to their environment and RNA folding can be influenced by ionic concentrations, long-distance interactions, and noncanonical base pairing. Furthermore, this study is limited to view the state of RNAs at the time of probing. Also, when proteins interact with RNA, structures can become perturbed and alternative conformations may form to complicate the interpretation of the reactivity information. However, some protein-RNA interactions are transient and have low specificity, which is true for protein scanning domains utilized for localization before high-affinity binding occurs [55]. Additionally, the spliceosome may also introduce alternative RNA conformations throughout the different stages of splicing, such intron removal and exon ligation. When the spliceosome is inhibited, the splicing of all transcripts is impacted, which could affect the distribution of splicing factors interacting with the pre-mRNA at the time of interrogation with DMS.

## Conclusion

The latent membrane protein 1 (*LMP1*) pre-mRNA of the Epstein–Barr virus (EBV) is a critical factor in viral latency and oncogenesis, yet the structural elements governing its complex splicing regulation have remained largely unknown. By applying the DMS-informed SIRP-seq method, coupled with RNAfold and ScanFold analysis, we have generated high-resolution secondary structure models for both the full-length (*LMP1-FL*) and alternatively spliced (*LMP1-AS*) isoforms of the *LMP1* pre-mRNA. Our results define novel, stable RNA secondary structural elements, particularly near the *LMP1* splice junctions. We hypothesize the structures near the splice junctions play a role as splicing regulatory elements, although their potential functions are not fully explored in this study. ScanFold analysis identified 11 thermodynamically stable RNA structures (low z-scores) across *LMP1*. Three structures (Structures 8, 9, and 10) were predicted near the 3′SS of intron 2 and within alternative conformations identified with the DRACO algorithm. These alternative conformations map to regions of interest where disease-relevant mutations occur in Burkitt’s Lymphoma patients. Structure 9 was suggested to have a role in splicing regulation, based on its alternative conformations at the 3′SS of intron 2. By identifying these novel RNA structures within the *LMP1* pre-mRNA, our work provides a structural framework to aid in understanding EBV pathogenesis. Future functional studies could focus on mutating these defined ScanFold structures to validate their role in *LMP1* splicing efficiency, particularly at the 3′SS of intron 2. Such studies may reveal novel therapeutic targets that exploit the *LMP1* structure to disrupt viral gene expression and mitigate EBV-associated malignancies.

## Supporting information

S1 Fig RT-PCR targeting strategy and validation of *LMP1* isoforms. The illustrated PCR targeting guide provides a schematic for the various products used to capture the *LMP1* pre-mRNA. Panel A displays the full-length *LMP1* cartoon with annotated lengths for intron 1 at 76 bp and intron 2 at 78 bp. Panels B through E detail the cartoons for Products 1, 2, and 3, showing the forward and reverse primers with respect to the full-length transcript along with their anticipated product sizes for unspliced, spliced, and alternatively spliced variants. The targeted regions and final product sizes are indicated to the right of each schematic.
(TIF)

S2 Fig Coverage for the transcriptomic and targeted sequencing of *LMP1.* (A) *LMP1* full-length pre-mRNA cartoon. (B) The 4-hour merged coverage for the full-length *LMP1* pre-mRNA, with a max read depth of 304199. (C) The DMS+ (top) and DMS- (bottom) transcriptomic coverage for the full-length *LMP1* pre-mRNA, with a max read depth of 3045 and 2000, respectively. (D) *LMP1* alternatively spliced pre-mRNA cartoon. (E) The 4-hour merged coverage for the alternatively spliced *LMP1* pre-mRNA. (F) The DMS+ (top) and DMS- (bottom) transcriptomic coverage for the alternatively spliced *LMP1* pre-mRNA, with a max read depth of 3045 and 2000, respectively.
(TIF)

S3 Fig Weblogos for the aligned BV-BRC sequences for ScanFold structures. Sequence logos generated from the aligned BV-BRC sequences demonstrate that the sequences for each of the eleven ScanFold structures are highly conserved. The height of each nucleotide on the bit score scale indicates its abundance in the aligned patient-derived sequences. Alignment gaps are represented by reduced bit scores, and a specific cytosine insertion in structure 7 is highlighted at position 603, resulting in a single nucleotide shift in the sequence position.
(TIF)

S4 Fig Sequencing libraries for the merged 4-Hour datasets mapped to *LMP1.* This figure provides a comparative view of the sequencing read coverage for the merged 4-hour datasets. Panel A shows the reference cartoon and scale for the full-length *LMP1* pre-mRNA transcript. Panel B illustrates that the mapped *LMP1-AS* reads show low coverage across intron 1 while maintaining increased coverage across intron 2. In contrast, Panel C shows that the mapped *LMP1-FL* reads provide consistent coverage across all targeted regions of the pre-mRNA.
(TIF)

S5 Fig DRACO conformations for the merged 4-Hour *LMP1-FL* dataset at the intron 1 3′SS. The DRACO program was used to identify potential alternative RNA structures near the splice site junctions. Conformations c0, c1, and c2 are displayed with their stoichiometric abundances shown on the left of each model. The DMS reactivity scale is set from 0 to 1.0, with red indicating higher reactivity to increase contrast for lower values after normalization. These conformations illustrate how different folding patterns may position the 3′SS at different locations, potentially impacting its accessibility to the splicing machinery.
(TIF)

S6 Fig DRACO conformations for the merged 4-Hour *LMP1-FL* dataset at the intron 2 3′SS. These panels display the alternative conformations identified for the full-length transcript near the 3′ splice junction of intron 2. Conformation c0 contains ScanFold Structure 10, while conformations c1 and c2 contain Structure 9. Each conformation is shown with its stoichiometric abundance and DMS reactivity values mapped to the structure models. These alternative states demonstrate the structural flexibility surrounding key mutation sites.
(TIF)

S7 Fig DRACO conformations for the merged 4-Hour *LMP1-AS* dataset at the intron 2 3′SS. This figure shows the conformations identified for the alternatively spliced isoform at the 3′ splice junction of intron 2. Conformation c0 is predicted to contain Structure 10, whereas conformation c1 contains both Structure 9 and Structure 10 within a branched loop structure. The stoichiometric abundances are provided on the left, and the structures are colored according to normalized DMS reactivity to highlight the accessibility of the splice junction.
(TIF)

S1 Table PCR Primers and cycling conditions for *LMP1* amplification. This table lists the specific forward and reverse primer sequences used to target three distinct regions of the *LMP1* transcript, designated as Product 1, Product 2, and Product 3. It details the PCR stage settings, including temperatures and durations for initial denaturation, cycling, and the final extension step. These conditions were optimized using Q5 High-Fidelity DNA Polymerase to ensure the accurate capture of various isoforms.
(DOCX)

S2 Table Pearson correlation analysis of *LMP1* isoform metrics. A Pearson correlation analysis was performed to evaluate the consistency of structural metrics between the *LMP1-FL* and *LMP1-AS* datasets. The table presents correlation coefficients for per-nucleotide z-scores, ensemble diversity values, and DMS reactivity values across overlapping regions of the transcripts. The high correlation values reported across these metrics indicate that the local RNA folding remains highly consistent regardless of the presence or absence of the upstream intron 1.
(XLSX)

S1 Protocol Step-by-step methodology for spliceosome inhibition and DMS-MaPseq. This protocol provides a comprehensive guide to the experimental procedures used to capture *LMP1* pre-mRNA structural data from cultured cells. It details the preparation of the spliceosome inhibitor pladienolide B, including specific concentrations and incubation times required to induce intron retention while maintaining cell viability. The document further outlines the DMS chemical probing steps, specifying the 1-minute reaction time and the subsequent quenching process using dithiothreitol.
(DOCX)

S1 Appendix Bioanalyzer validation of *LMP1* isoforms. This appendix provides a sample of the initial Agilent 2100 Bioanalyzer electropherogram results derived from 35-cycle PCR reactions used to identify the alternative isoform of *LMP1*. It includes comparative data for multiple samples across 0-hour, 2-hour, and 4-hour time points for PCR products 1, 2, and 3. The section also presents raw and annotated gel images to confirm the successful capture of different splicing variants.
(PDF)

S2 Appendix PCR product extraction and DNA yields. Appendix S2 contains gel images documenting the extraction of PCR products used to build the sequencing libraries. It features both unannotated and annotated images for 30-cycle PCR reactions across various sample time points. A detailed summary table is included to report the final DNA concentrations and total yields for each recovered band, distinguishing between spliced, unspliced, and alternatively spliced targets.
(PDF)

S3 Appendix Library preparation and fragment size analysis. This appendix serves as a guide for the Illumina DNA library preparation and pooling process. It includes the Bioanalyzer results used for fragment size analysis to ensure a normalized distribution of DNA fragment sizes prior to sequencing on the iSeq100. These quality control steps were necessary to confirm the average fragment size for each of the sixteen individual libraries.
(ZIP)

S1 Dataset Compiled sequencing and structural analysis metrics. The S1 Dataset is a complete repository of the sequencing data and processed results from the RNAframework and ScanFold analyses. It contains the Phred quality scores for the iSeq100 reads as well as the comprehensive output from the sliding window structural assessments. This dataset provides foundational metrics such as z-scores, ensemble diversity, and reactivities used to generate the high-resolution *LMP1* models. These files can all be found on Zenodo: https://doi.org/10.5281/zenodo.18841968.
(TXT)

S1 File DRACO conformational clusters and abundances for the *LMP1-FL* dataset. This file provides the specific DRACO regions and stoichiometric abundances for the merged 4-hour *LMP1-FL* dataset. It details the alternative RNA conformations identified by spectral clustering, highlighting distinct structural profiles found near splice site junctions. These data support the observation of alternative structures that may influence splicing accessibility.
(TXT)

S2 File DRACO conformational clusters and abundances for the *LMP1-AS* dataset. This file provides the specific DRACO regions and stoichiometric abundances for the merged 4-hour *LMP1-AS* dataset. It details the alternative RNA conformations identified by spectral clustering, highlighting distinct structural profiles found near splice site junctions. These data support the observation of alternative structures that may influence splicing accessibility.
(TXT)

S3 File *LMP1* sequence alignment. The full-length *LMP1* sequence alignment derived from the sequences obtained from the BV-BRC database.
(FASTA)

S4 File Sequence alignments for ScanFold structures. Alignments used in the conservation analyses of ScanFold extracted structures in FASTA format.
(7Z)

S5 File Conserved structural regions identified with RNAalifold. Conserved structural regions 1 and 2 were obtained from RNAalifold using the sequence alignment file, and regions 1 and 2 were annotated at positions (705–720) and (1032–1074).
(DBN)
