# Supplementary material for: DMS-informed secondary structure modeling of Epstein–Barr Virus LMP-1 pre-mRNA defines novel elements spanning introns
Source: PLoS One. 2026 Jul 2;21(7):e0345208. doi: 10.1371/journal.pone.0345208 (PMC13327190; doi:10.1371/journal.pone.0345208)
Supplement: S1 Table — This table lists the specific forward and reverse primer sequences used to target three distinct regions of the LMP1 transcript, designated as Product 1, Product 2, and Product 3. It details the PCR stage settings, including temperatures and durations for initial denaturation, cycling, and the final extension step. These conditions were optimized using Q5 High-Fidelity DNA Polymerase to ensure the accurate capture of various isoforms. (DOCX) [file pone.0345208.s008.docx]

| Primer Name | Primer Sequence | Stage 1 | Stage 2 | | | Stage 3 | Stage 4 |
| --- | --- | --- | --- | --- | --- | --- | --- |
| LMP1_FWD1 | cggccctacatcccaagaaa | 98 | 98 | 59 | 72 | 72 | 4 |
| LMP1_REV1 | gcacccgaagatgaacagca | 98 | 98 | 59 | 72 | 72 | 4 |
| LMP1_FWD2 | tggagccctttgtctactccta | 98 | 98 | 59 | 72 | 72 | 4 |
| LMP1_REV2 | tctgccctcgttggagttag | 98 | 98 | 59 | 72 | 72 | 4 |
| LMP1_FWD3 | cgctctctggaatttgcacg | 98 | 98 | 59 | 72 | 72 | 4 |
| LMP1_REV3 | tggtgggcctccatcatttc | 98 | 98 | 59 | 72 | 72 | 4 |
|  | Time (s) | 30 | 10 | 15 | 20 | 120 | ∞ |
|  |  | Initial Denature | Denature | Annealing | Extension | Final Extension | Incubation |
|  |  |  | Number of Cycles (30-35) | | |  |  |

S1 Table. PCR cycle conditions
