# Supplementary material for: DMS-informed secondary structure modeling of Epstein–Barr Virus LMP-1 pre-mRNA defines novel elements spanning introns
Source: PLoS One. 2026 Jul 2;21(7):e0345208. doi: 10.1371/journal.pone.0345208 (PMC13327190; doi:10.1371/journal.pone.0345208)
Supplement: S1 Protocol — This protocol provides a comprehensive guide to the experimental procedures used to capture LMP1 pre-mRNA structural data from cultured cells. It details the preparation of the spliceosome inhibitor pladienolide B, including specific concentrations and incubation times required to induce intron retention while maintaining cell viability. The document further outlines the DMS chemical probing steps, specifying the 1-minute reaction time and the subsequent quenching process using dithiothreitol. (DOCX) [file pone.0345208.s010.docx]

**Protocol Outline for SIRP-seq**

The steps of the SIRP-seq protocol can be optimized further for different cell types. This protocol outlines example conditions used for adherent cells (HeLa) and suspension cells (BJAB-B1).

**Suggested Starting Reagents**

- Dimethyl Sulfoxide (DMSO), Pladienolide B, Dimethyl Sulfate (DMS), Dithiothreitol (DTT), DPBS, and TRIzol reagent

**Step-by-Step Protocol**

**I. Cell Culture**

**Adherent and Suspension Cell Lines:**

- - Maintain cells normally with complete growth media following standard cell culture practices.
  - Ensure cells are in an active growth state at 70-90% confluence or optimal cell density before starting the cell plating step.

**II. Cell Plating**

Note: 6-well and 12-well plates work well for probing in replicates with adherent cells and for the incubation step with suspension cells.

**Adherent Cell Line:**

- - Trypsinize, quench, and resuspend cells into a single cell suspension using media. Count the total population of cells using a cell counter and normalize the cell density.
  - Plate an equivalent number of cells at a density that will allow them to reach approximately 70-80% confluence on the day of treatment.
  - Incubate cells 18-24 hours at 37°C in an incubator with 5% CO_2_ to allow the cells to adhere to the bottom of the wells.
  - If low volumes are used, add a parameter of PBS to the outside wells surrounding the wells with cells to reduce media evaporation.

**Suspension Cell Line:**

(Review Section III: Treatment Preparation before continuing)

- - Resuspend the cells, count the total number of cells, and normalize the cell density.
  - Centrifuge the desired number of cells into new tubes for 3 minutes at 200xg. Remove the supernatant without disturbing the pellet.
  - Individually resuspend the cell pellet from each of the tubes using the prepared treatment media. Plate the cells into a new well of a marked plate and incubate.

**III. Treatment Preparation**

1. **Prepare a Working Solution:**
   - The final concentration of pladienolide B in the media can be 350 nM.
   - The 100x working solution 35 µM. Dilute 1:100 in media to achieve 350 nM.
   - If starting with a stock concentration of 1 mM, 100 µL of a 100x working solution can be made with the calculations below.
2. **Calculations for a 100x Working Solution:**
   - Dilute your 1 mM stock solution in DMSO to achieve a 35 µM solution.

$$\frac{\left[ M2 \right]*V2}{\left[ M1 \right]} = V1$$

If *M2* = 35 µM, *V2* = 100 µL, and *M1* = 1000 µM, then *V1* = 3.5 µL

- - Add 3.5 µL of 1 mM stock to 96.5 µL of DMSO to make 100X solution.

1. **Prepare DTT Quench Solution:**

The DTT quench should be at a molar concentration 5x molar excess relative to the DMS in solution. If 0.5 mL of 2% DMS is used, two 1.0 mL aliquots of the 5x DTT solution can be used for quenching.

- - Use the molarity of the 2% DMS to determine how much DTT to make.

$$\frac{2}{100}*\frac{\rho_{DMS}}{{MW}_{DMS}}=[DMS]$$

The density ($\rho$) of DMS = 1.33 g/mL, its molecular weight (*MW*) = 126.13 g/mol, and the concentration of a 2% DMS solution is 0.211 M.

The DTT solution should be approximately 5 * 0.211 M = **1.055 M DTT**. Dissolve DTT into DPBS to get a ~1.06 M solution. Place it into a water bath and keep nearby during probing. Prepare enough DTT quench solution for two quenches for each sample, with extra amounts for quenching the residual DMS on surfaces. Aim for about 15-20 mL of 1.1 M DTT.

1. **Prepare 2% DMS Solution:**

Since DMS is a highly toxic and cell permeable carcinogen, review its safety data sheets, only use it in a fume hood, and practice caution while handling it. DMS needs to be quenched, and the volume used should be minimized.

**Selecting the volume to use:**

The volume required for adherent cells is dependent on the surface area of the dish used for plating, while the volume for suspension cells can be reduced to 0.5 mL for easy handling with a p1000 pipet.

**Recipe for 2% DMS**

- - To make 10 mL of 2% DMS: Add 200 µL of >99% DMS to 9.8 mL of the appropriate buffer (e.g. DPBS mixed with 25% ethanol). Scale as necessary for experiments.

Note: The ethanol is added to break down the DMS, since it does not easily dissolve in DPBS. It is recommended to make the 2% DMS solution in a larger tube (at least 1.5X the volume of the prepared solution), to prevent liquid overflow from additional pipetting and vortexing.

**IV. Treatment Incubation**

General incubation times between 0-hours and 4-hours can be used with 350 nM pladienolide B. Longer incubations (24-72 hours) and higher concentrations of pladienolide B can increase the negative impacts to cell viability. Stagger the incubation times between time points. Allow for at least 20 minutes, depending on the number of replicate samples that need to be probed. More time should be allowed if spinning is required to remove the treatment media.

**Adherent Cell Line:**

- - On the day after plating, begin by aspirating the media from the cells.
  - Wash the cells once gently with DPBS.
  - Add freshly made treatment media containing diluted pladienolide B.
    - Example: For 2 mL, add 20 µL of your 100x working solution to 1.98 mL of media.
  - For the DMSO control, add 20 µL of DMSO to 1.98 mL of media.
  - Once the time has been reached, remove the treatment media and proceed to the DMS probing step.

**Suspension Cell Line:**

- - After resuspending the cells into the prepared treatment media, incubate the cells for the desired time points.
  - When removing the treatment media from suspension cells, a spin step is required to remove treatment media before probing.

**V. DMS Probing Procedure (Perform all steps in a fume hood):**

For each sample:

1. Carefully aspirate the treatment media from the cells or cell pellet.
2. Set the timer to 1 minute. Add the 2% DMS solution, which should completely cover the cells. For suspension cells, gently resuspend the pellet using the DMS solution.

Incubate at room temperature for **exactly 1 minute**.

1. Immediately quench the DMS reaction by carefully dispensing the DTT solution directly into the reaction. Avoid splashing.

Repeat steps 1-3 for each sample before proceeding to step 4. For suspension cells, the cells will need to be spun down before removing the quenched DMS/DTT quench solution (see below).

1. Remove the quenched DMS/DTT solution into the designated container marked “DMS liquid waste” in the fume hood. For suspension cells, skip this step.
2. Repeat the DTT quench step for each sample, this time ensure all surfaces that encountered the DMS solution are carefully washed with the quench solution.
3. Repeat step 4. For suspension cells, pellet the cells before removing the entire quenched DMS and DTT solution.
4. Collect cellular lysate containing the RNA by adding an appropriate amount of TRIzol reagent (~400 µL). For suspension cells, resuspend the pellet in the TRIzol reagent.
5. Store on ice for subsequent processing, or at -80°C for long-term storage. Avoid unnecessary freeze/thaw steps to preserve RNA integrity prior to reverse transcription.

Proceed with your downstream applications (RNA isolation and purification, RIN analysis, RT-PCR, library preparation, etc.).
