# Supplementary material for: DMS-informed secondary structure modeling of Epstein–Barr Virus LMP-1 pre-mRNA defines novel elements spanning introns
Source: PLoS One. 2026 Jul 2;21(7):e0345208. doi: 10.1371/journal.pone.0345208 (PMC13327190; doi:10.1371/journal.pone.0345208)
Supplement: S1 Appendix — This appendix provides a sample of the initial Agilent 2100 Bioanalyzer electropherogram results derived from 35-cycle PCR reactions used to identify the alternative isoform of LMP1. It includes comparative data for multiple samples across 0-hour, 2-hour, and 4-hour time points for PCR products 1, 2, and 3. The section also presents raw and annotated gel images to confirm the successful capture of different splicing variants. (PDF) [file pone.0345208.s011.pdf]

S1 Appendix

This appendix contains a sample of the initial Agilent 2100 Bioanalyzer electropherogram results from the 35-cycle PCR reactions that were used to identify the alternative isoform of *LMP1*. For Figures S1-S3, the PCR products for sample 1 (A-C) and sample 2 (D-F) are shown for each time point, while product 1 is shown in panels A and D, product 2 is shown in panels B and E, and product 3 is shown in panels C and F.

Figure S1. 0-hour time point results.
Figure S2. 2-hour time point results.
Figure S3. 4-hour time point results.
Figure S4. Example of the ladder used for the integration of peaks.
Figure S5. Raw unannotated gel images for the 35-cycle PCR reactions. (Top) Gel image for the 0-hour samples and 2-hour sample 1. (Bottom) Gel image for 2-hour sample 2 and the 4-hour samples.
Figure S6. Annotated gel images for the 35-cycle PCR reactions.

Figure S1.

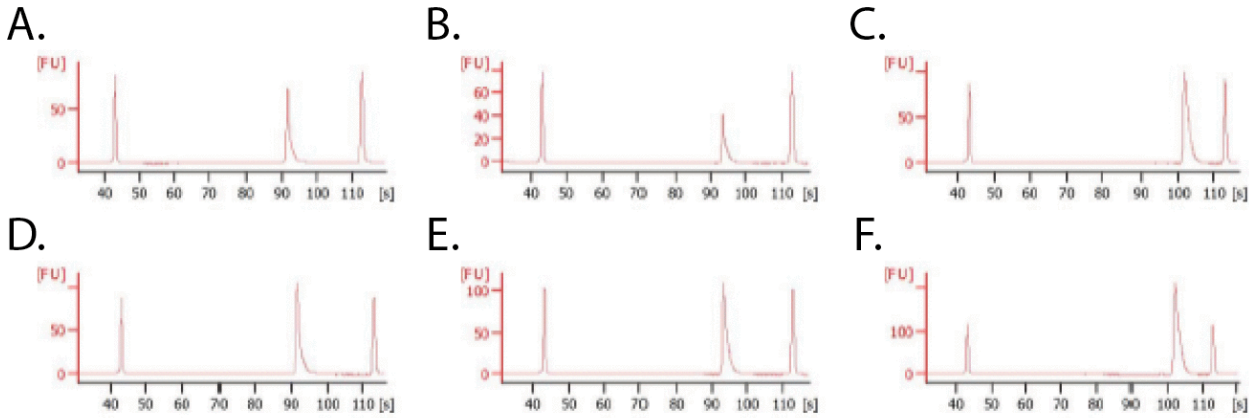

Figure S2.

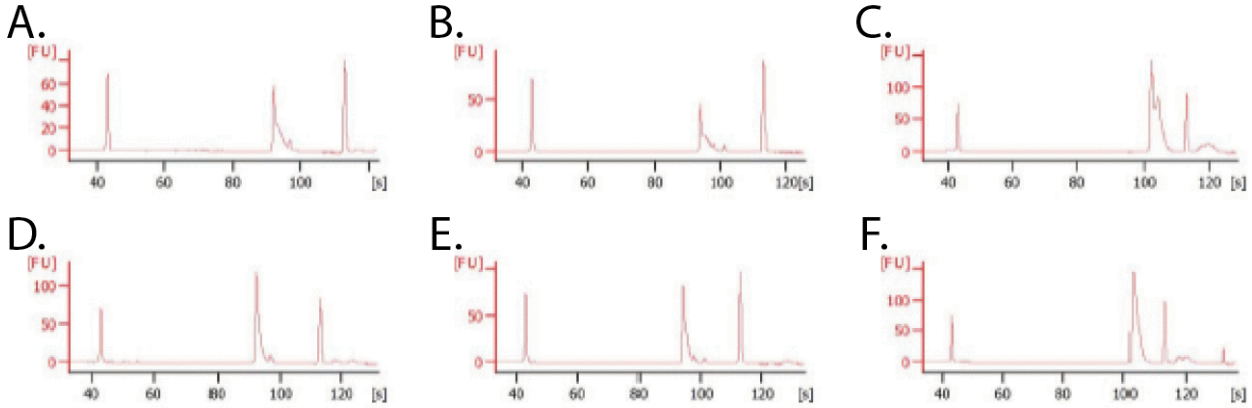

Figure S3.

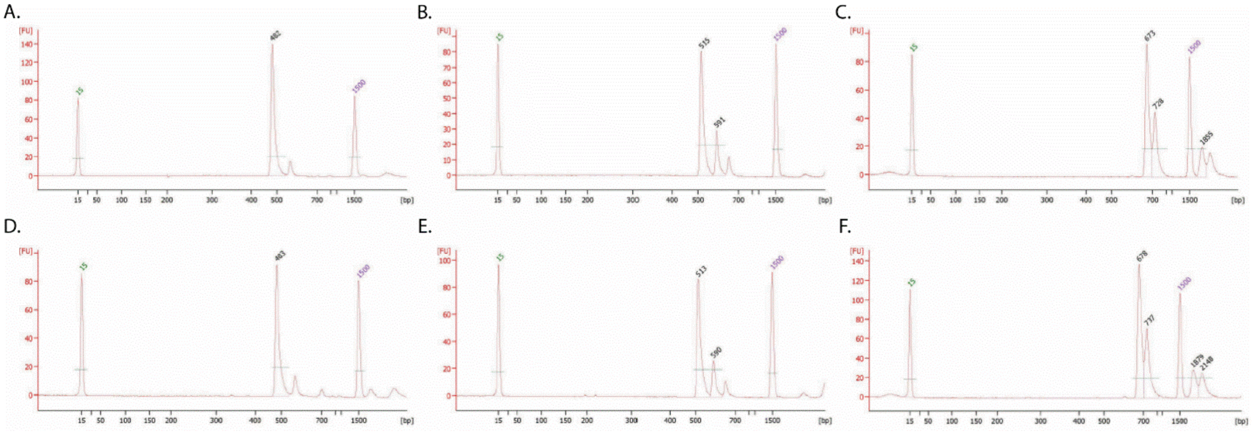

Figure S4.

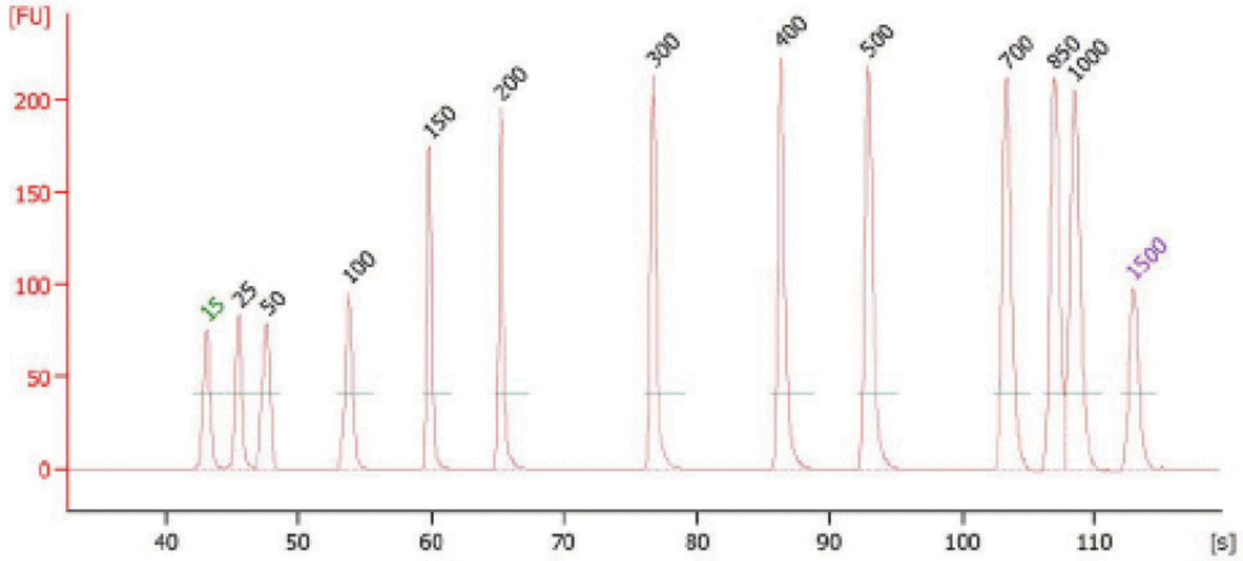

[FU]
200
150
100
50
0
15
25
50
100
150
200
300
400
500
700
850
1000
1500
40
50
60
70
80
90
100
110
[s]

Figure S5.

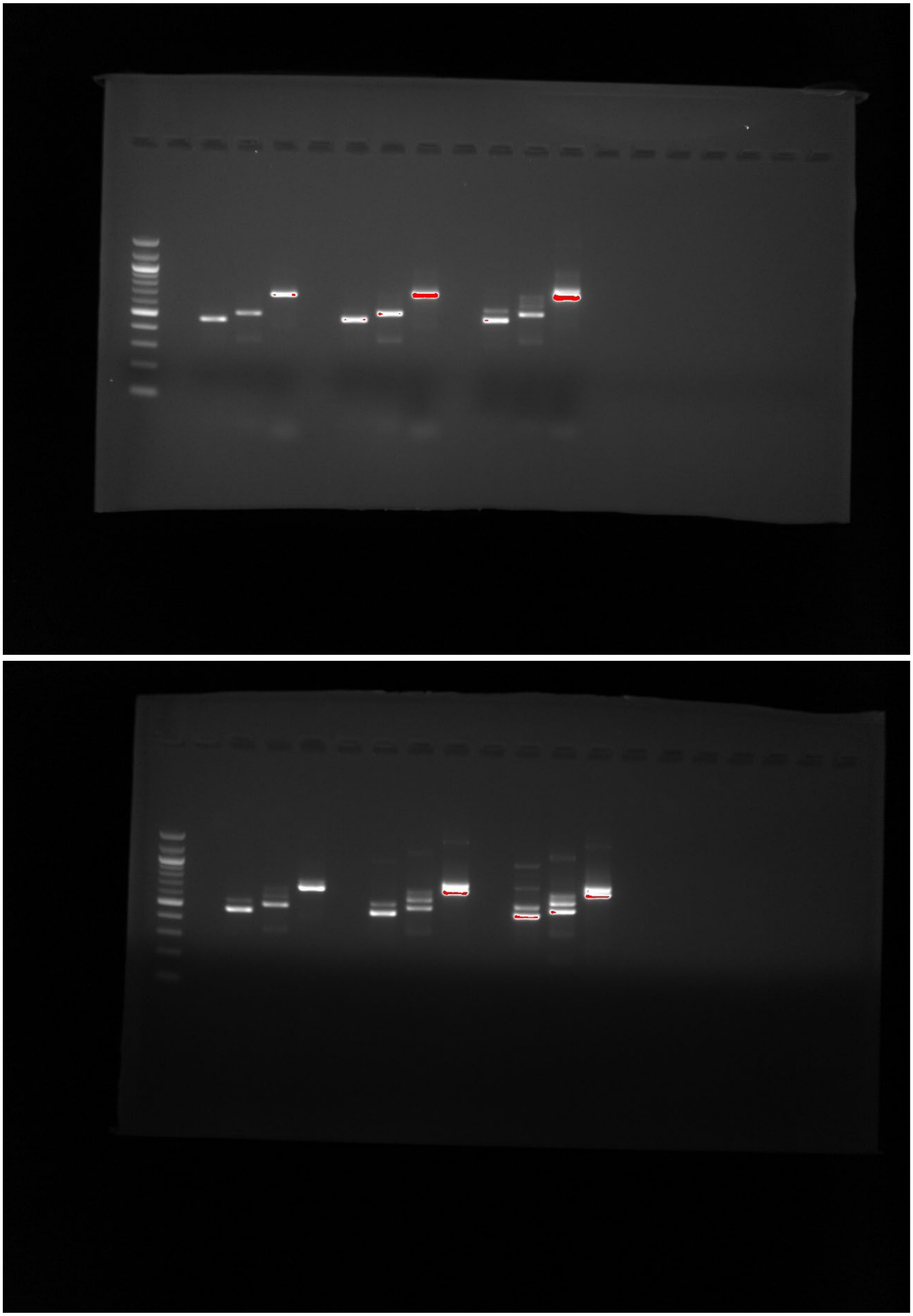

Figure S6.

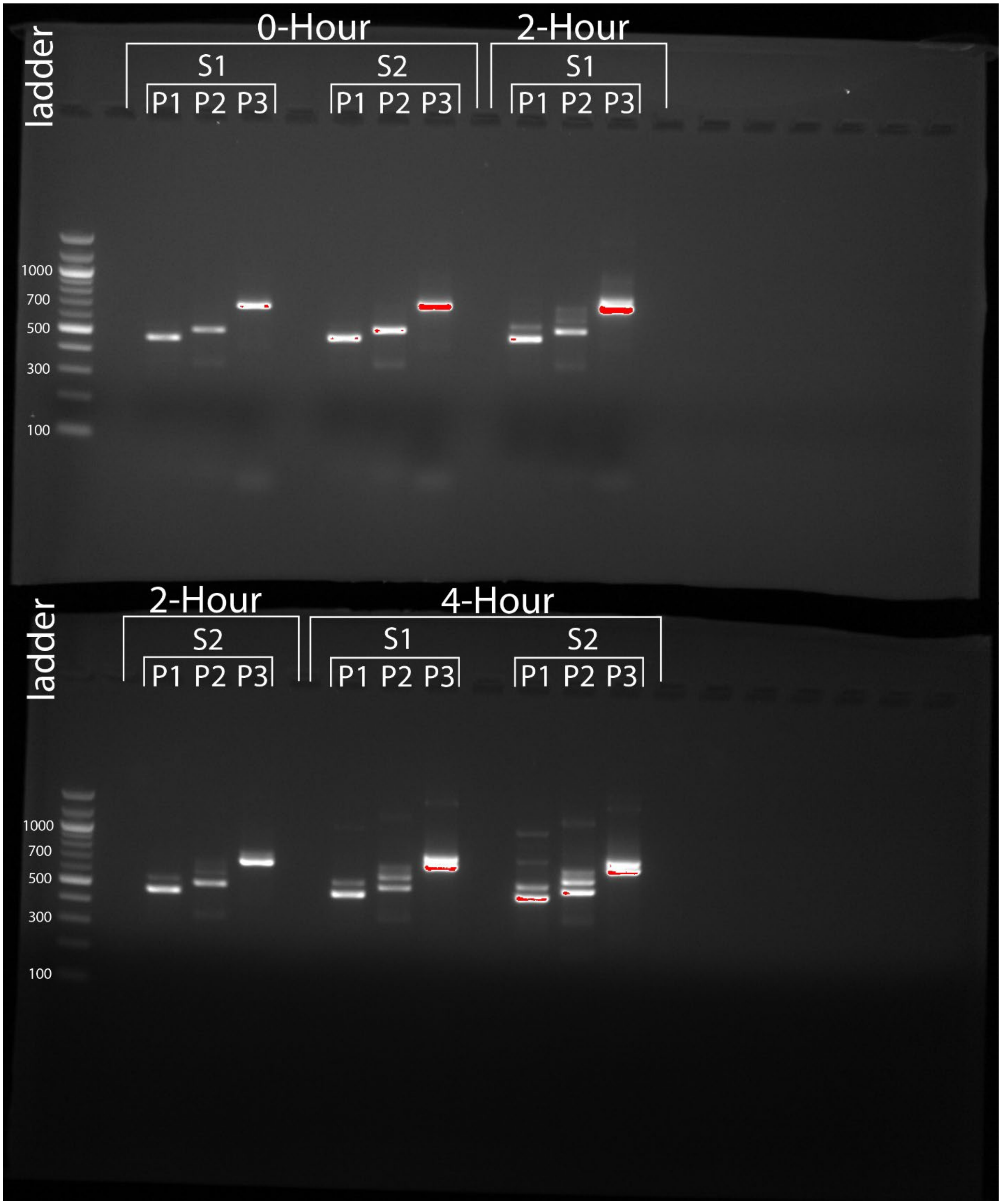
ladder
0-Hour
2-Hour
S1
S2
S1
P1 P2 P3
P1 P2 P3
P1 P2 P3
1000
700
500
300
100
ladder
2-Hour
4-Hour
S2
S1
S2
P1 P2 P3
P1 P2 P3
P1 P2 P3
1000
700
500
300
100
