# Supplementary material for: DMS-informed secondary structure modeling of Epstein–Barr Virus LMP-1 pre-mRNA defines novel elements spanning introns
Source: PLoS One. 2026 Jul 2;21(7):e0345208. doi: 10.1371/journal.pone.0345208 (PMC13327190; doi:10.1371/journal.pone.0345208)
Supplement: S2 Appendix — Appendix S2 contains gel images documenting the extraction of PCR products used to build the sequencing libraries. It features both unannotated and annotated images for 30-cycle PCR reactions across various sample time points. A detailed summary table is included to report the final DNA concentrations and total yields for each recovered band, distinguishing between spliced, unspliced, and alternatively spliced targets. (PDF) [file pone.0345208.s012.pdf]

S2 Appendix

This appendix file contains images of the gels used for extracting the PCR products used for sequencing library preparation and the yields for each extracted band that was pooled for sequencing.

Figure S1. Raw unannotated gel images for the 30-cycle PCR reactions. (Top) Gel image for the 0-hour and 2-hour samples. (Bottom) Gel image for the 4-hour samples.
Figure S2. Annotated gel images for the 30-cycle PCR reactions.
Figure S3. Raw unannotated gel images for the 30-cycle PCR reactions that were used for 20 ul extractions. (Top) Gel image for the 0-hour samples and 2-hour sample 1.
(Bottom) Gel image for 2-hour sample 2 and the 4-hour samples.
Figure S4. Raw unannotated gel images for the 30-cycle PCR reactions used for 10 ul extractions. (Top) Gel image for the 0-hour samples and 2-hour sample 1. (Bottom) Gel image for 2-hour sample 2 and the 4-hour samples.
Figure S5. Annotated gel images for the gel extractions.
Table S1. Gel extraction yields for recovered PCR product bands.

Figure S1

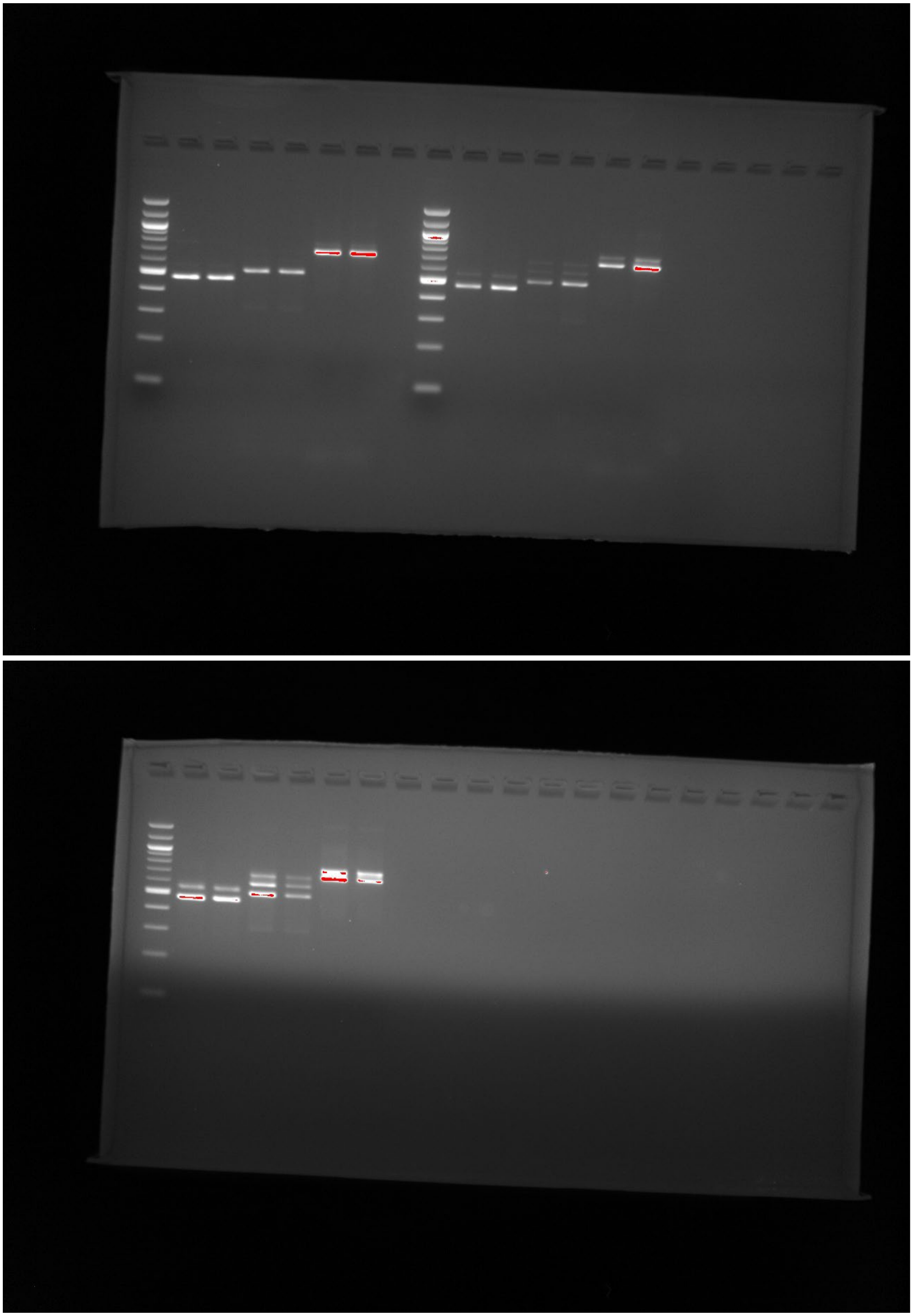

Figure S2

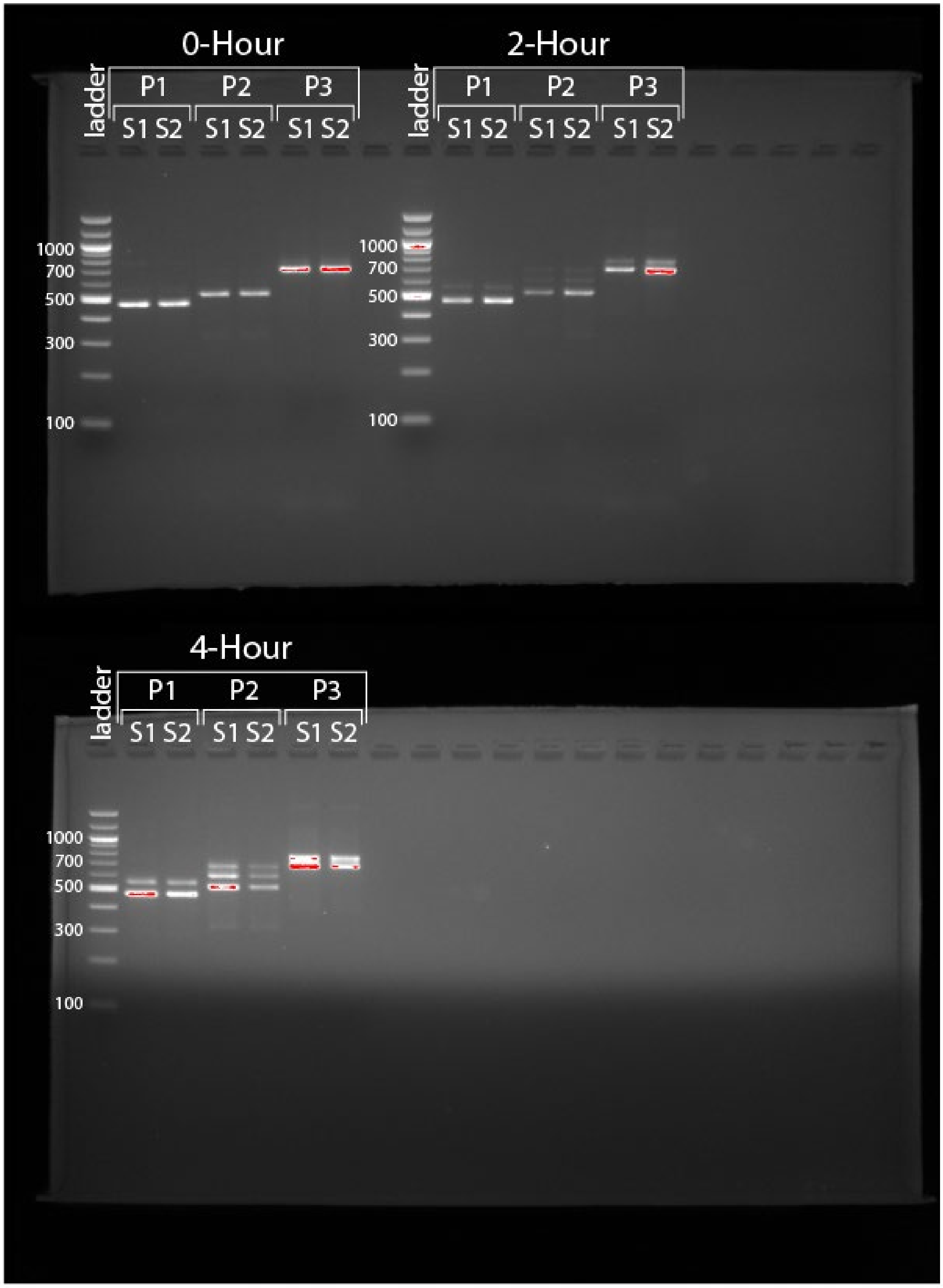

Figure S3

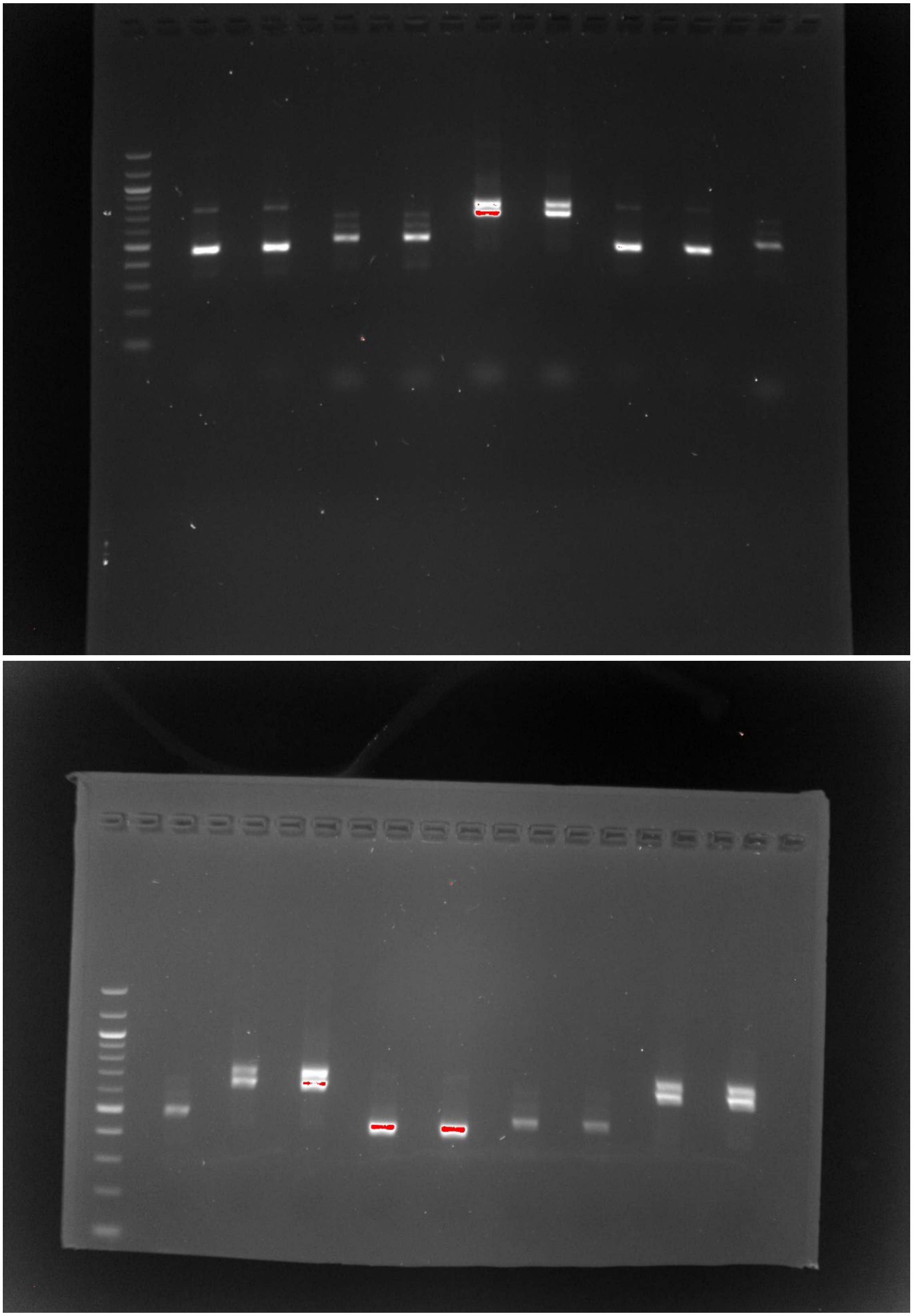

Figure S4

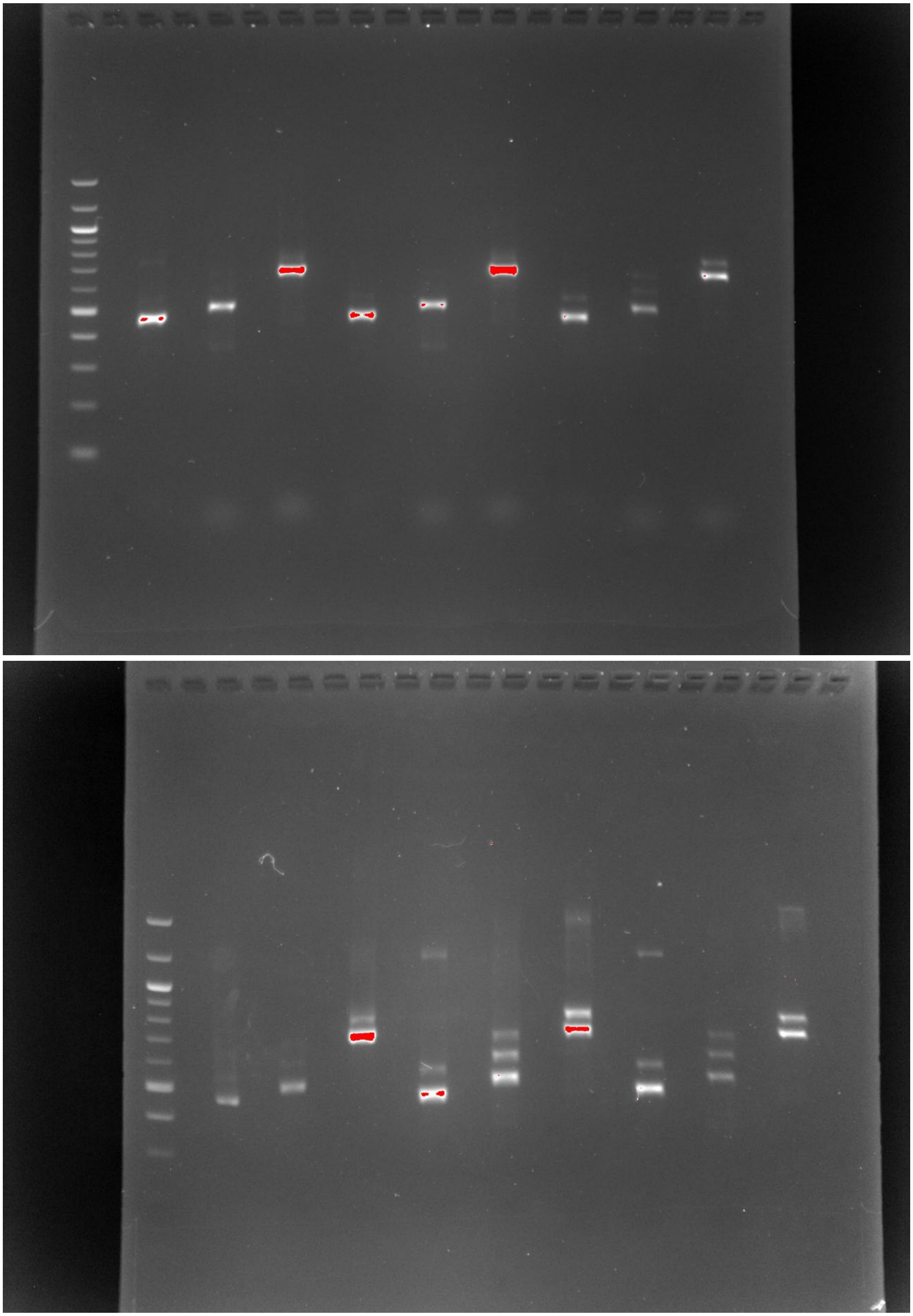

Figure S5

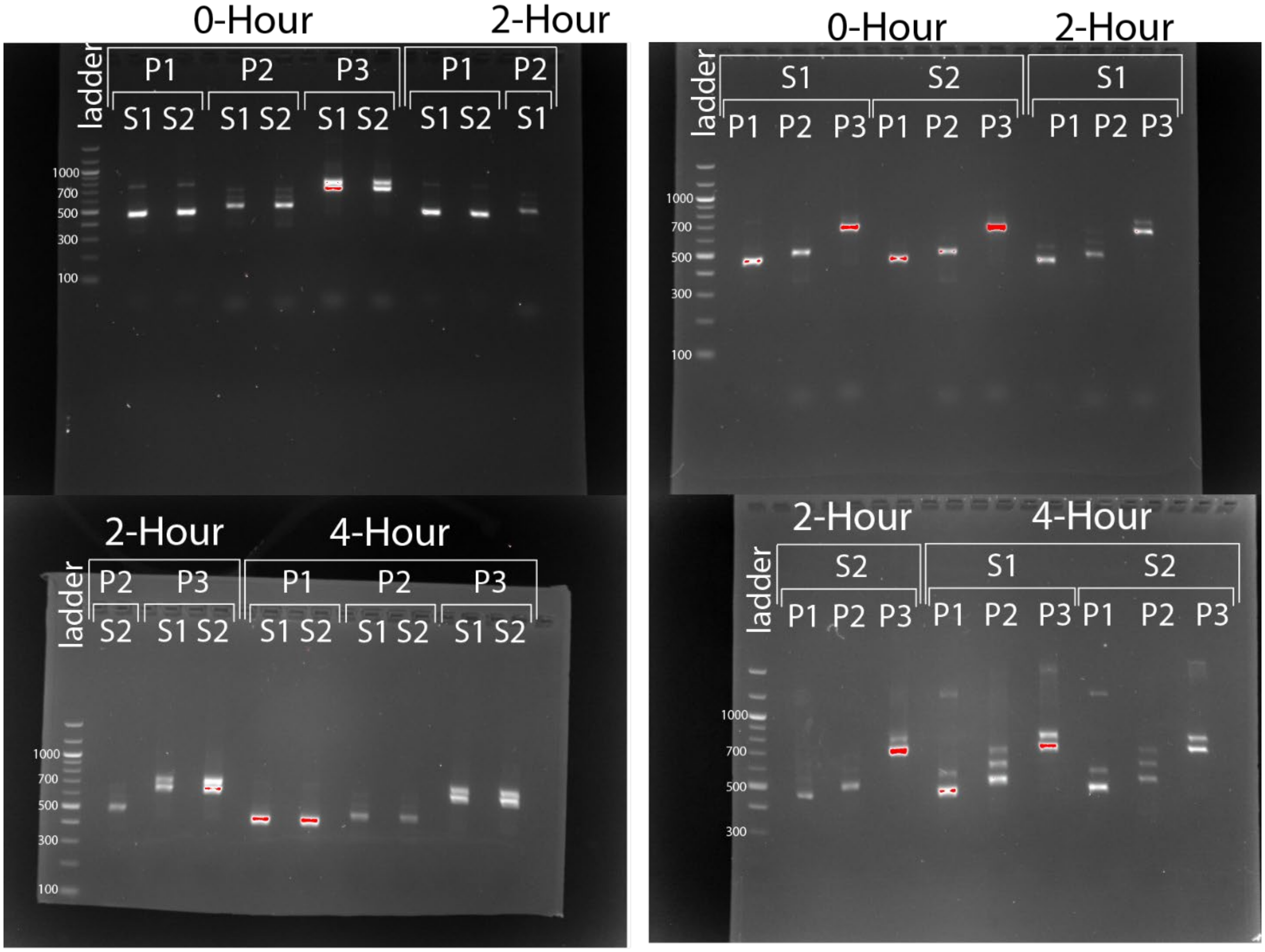
0-Hour
2-Hour
ladder
P1 P2 P3 P1 P2
S1 S2 S1 S2 S1 S2 S1 S2 S1
1000
700
500
300
100
0-Hour
2-Hour
ladder
S1 S2 S1
P1 P2 P3 P1 P2 P3 P1 P2 P3
1000
700
500
300
100
2-Hour
4-Hour
ladder
P2 P3 P1 P2 P3
S2 S1 S2 S1 S2 S1 S2 S1 S2
1000
700
500
300
100
2-Hour
4-Hour
ladder
S2 S1 S2
P1 P2 P3 P1 P2 P3 P1 P2 P3
1000
700
500
300

Table S1

Recovered Gel Sample	Time Point	Splicing Status	Target	Concentration (ng/uL)	Total DNA
1	0 Hr	Spliced	P1	3.08	24.64
2	0 Hr	Spliced	P2	1.53	12.24
3	0 Hr	Spliced	P3	3.49	27.92
4	0 Hr	Spliced	P1	4.56	36.48
5	0 Hr	Spliced	P2	1.65	13.2
6	0 Hr	Spliced	P3	3.62	28.96
7	2 Hr	Spliced	P1	1.24	9.92
8	2 Hr	Unspliced	P1	0.092	0.736
9	2 Hr	Spliced	P2	0.691	5.528
10B	2 Hr	Alt. Spliced	P2	0.495	3.96
11B	2 Hr	Unspliced	P2	1.25	10
12	2 Hr	Spliced	P3	1.52	12.16
13	2 Hr	Unspliced	P3	0.228	1.824
14	2 Hr	Spliced	P1	1.19	9.52
15	2 Hr	Unspliced	P1	0.233	1.864
16	2 Hr	Spliced	P2	1.35	10.8
17	2 Hr	Alt. Spliced	P2	0.161	1.288
18B	2 Hr	Unspliced	P2	0.195	1.17
19	2 Hr	Spliced	P3	4.79	38.32
20	2 Hr	Unspliced	P3	0.719	5.752
21	4 Hr	Spliced	P1	3.07	24.56
22	4 Hr	Unspliced	P1	0.447	3.576
23	4 Hr	Spliced	P2	2.16	17.28
24	4 Hr	Alt. Spliced	P2	0.981	7.848
25	4 Hr	Unspliced	P2	0.438	3.504
26	4 Hr	Spliced	P3	1.88	15.04
27	4 Hr	Unspliced	P3	1.22	9.76
28	4 Hr	Spliced	P1	1.48	11.84
29	4 Hr	Unspliced	P1	0.288	2.304
30	4 Hr	Spliced	P2	0.524	4.192
31	4 Hr	Alt. Spliced	P2	0.302	2.416
32	4 Hr	Unspliced	P2	0.159	1.272
33	4 Hr	Spliced	P3	1.23	9.84
34	4 Hr	Unspliced	P3	0.815	6.52
35B	0 Hr	Unspliced	P3	3.85	30.8
36B	0 Hr	Unspliced	P3	2.5	20

Note: Sample ID (B) indicates a 20 uL reaction was used for gel recovery, due to low yields. All other samples are gel purified from 10 uL reactions.
