# Supplementary material for: DMS-informed secondary structure modeling of Epstein–Barr Virus LMP-1 pre-mRNA defines novel elements spanning introns
Source: PLoS One. 2026 Jul 2;21(7):e0345208. doi: 10.1371/journal.pone.0345208 (PMC13327190; doi:10.1371/journal.pone.0345208)
Supplement: S3 Appendix — This appendix serves as a guide for the Illumina DNA library preparation and pooling process. It includes the Bioanalyzer results used for fragment size analysis to ensure a normalized distribution of DNA fragment sizes prior to sequencing on the iSeq100. These quality control steps were necessary to confirm the average fragment size for each of the sixteen individual libraries. (ZIP) [file pone.0345208.s013.zip › S3_Appendix/S3_Bioanalyzer_Library_A.pdf]

Assay Class: High Sensitivity DNA Assay
Data Path: C:\...gh Sensitivity DNA Assay_DE23101764_2024-10-31_13-02-48.xad

Created: 10/31/2024 1:02:48 PM
Modified: 10/31/2024 1:44:09 PM

Electrophoresis File Run Summary

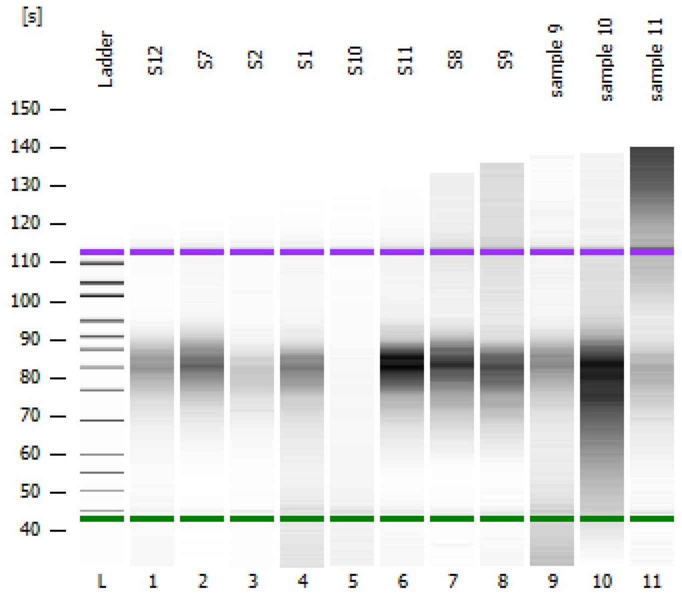

Instrument Information:

Instrument Name: DE23101764
Firmware: C.01.069
Serial#: DE23101764
Type: G2938B

Assay Information:

Assay Origin Path: C:\Program Files (x86)\Agilent\2100 bioanalyzer\2100 expert\assays\dsDNA\High Sensitivity DNA.xsy

Assay Class: High Sensitivity DNA Assay

Version: 1.03

Assay Comments: Copyright © 2003-2010 Agilent Technologies

Chip Information:

Chip Lot #: CU19BK50

Reagent Kit Lot #: 2402

Chip Comments:

S12

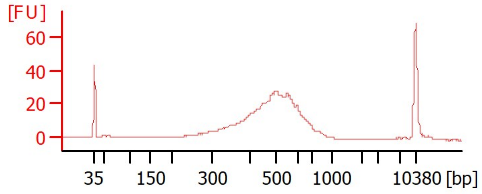

S7

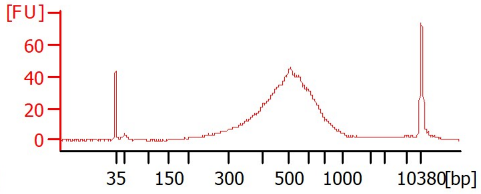

S2

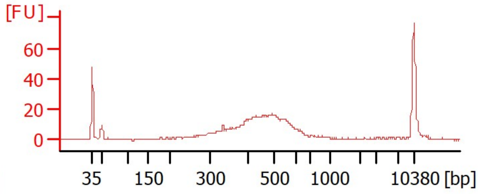

S1

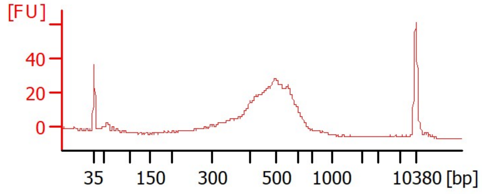

S10

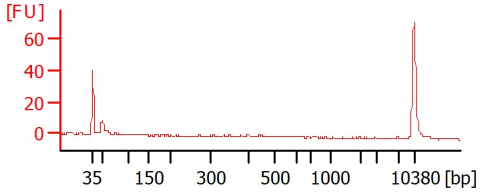

S11

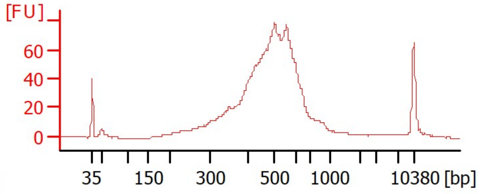

S8

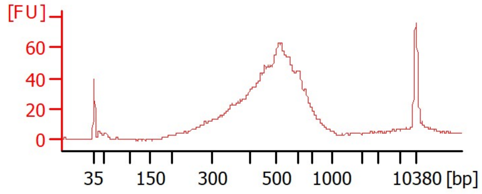

S9

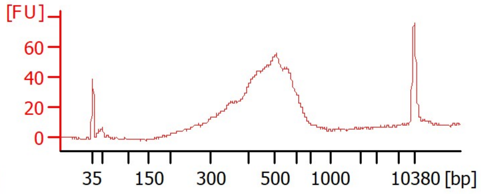

sample 9

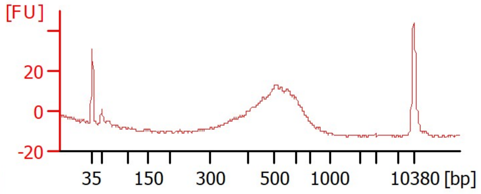

sample 10

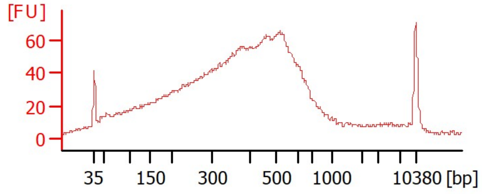

sample 11

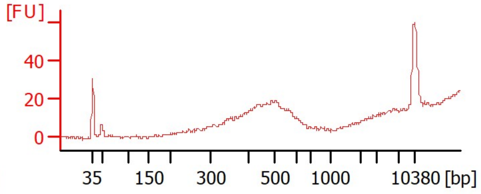

 © Copyright 2003 - 2024 Agilent, Inc.

Assay Class: High Sensitivity DNA Assay
Data Path: C:\...gh Sensitivity DNA Assay_DE23101764_2024-10-31_13-02-48.xad
Created: 10/31/2024 1:02:48 PM
Modified: 10/31/2024 1:44:09 PM

Electrophoresis File Run Summary (Chip Summary)

Sample Name	Sample Comment	Rest. Digest	Status	Observation	Result Label	Result Color
S12		☐	✔			
S7		☐	✔			
S2		☐	✔			
S1		☐	✔			
S10		☐	✔			
S11		☐	✔			
S8		☐	✔			
S9		☐	✔			
sample 9		☐	✔			
sample 10		☐	✔			
sample 11		☐	✔			
Ladder		☐	✔			

Chip Lot #	Reagent Kit Lot #
CU19BK50	2402

Chip Comments :

© Copyright 2003 - 2024 Agilent, Inc.

Assay Class: High Sensitivity DNA Assay
Data Path: C:\...gh Sensitivity DNA Assay_DE23101764_2024-10-31_13-02-48.xad
Created: 10/31/2024 1:02:48 PM
Modified: 10/31/2024 1:44:09 PM

Electrophoresis Assay Details

General Analysis Settings

Number of Available Sample and Ladder Wells (Max.) : 12
Minimum Visible Range [s] : 32
Maximum Visible Range [s] : 138
Start Analysis Time Range [s] : 33
End Analysis Time Range [s] : 137.5
Ladder Concentration [pg/µl] : 1950
Uses Standard Area for Ladder Fragments
Lower Marker Concentration [pg/µl] : 125
Upper Marker Concentration [pg/µl] : 75
Used Upper Marker for Quantitation
Standard Curve Fit is Point to Point
Show Data Aligned to Lower and Upper Marker

Integrator Settings

Integration Start Time [s] : 33.05
Integration End Time [s] : 137
Slope Threshold : 0.8
Height Threshold [FU] : 5
Area Threshold : 0.1
Width Threshold [s] : 0.6
Baseline Plateau [s] : 0.5

Filter Settings

Filter Width [s] : 0.5
Polynomial Order : 4

Ladder

	Size	Area
1	35	160.321
2	50	210.177
3	100	208.32
4	150	221.333
5	200	242.044
6	300	270.153
7	400	304.774
8	500	306.312
9	600	336.215
10	700	321.09
11	1000	365.785
12	2000	412.721
13	3000	411.427
14	7000	399.508
15	10380	213.613

 © Copyright 2003 - 2024 Agilent, Inc.

Assay Class: High Sensitivity DNA Assay
Data Path: C:\...gh Sensitivity DNA Assay_DE23101764_2024-10-31_13-02-48.xad

Created: 10/31/2024 1:02:48 PM
Modified: 10/31/2024 1:44:09 PM

Electropherogram Summary

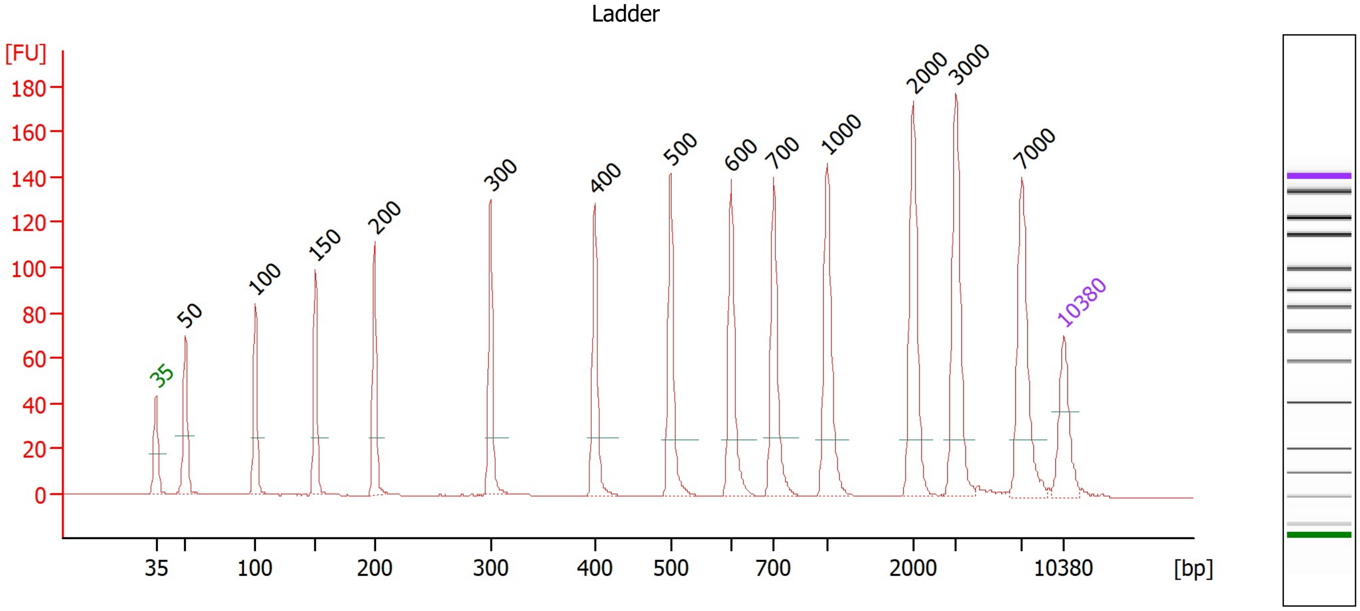

Overall Results for Ladder

Noise: 0.1

Peak table for Ladder

	Peak	Size [bp]	Conc. [pg/µl]	Molarity [pmol/l]	Observations
1		35	125	5411.25	Lower Marker
2		50	150	4545.45	Ladder Peak
3		100	150	2272.725	Ladder Peak
4		150	150	1515.15	Ladder Peak
5		200	150	1136.363	Ladder Peak
6		300	150	757.575	Ladder Peak
7		400	150	568.1813	Ladder Peak
8		500	150	454.545	Ladder Peak
9		600	150	378.7875	Ladder Peak
10		700	150	324.675	Ladder Peak
11		1000	150	227.2725	Ladder Peak
12		2000	150	113.6363	Ladder Peak
13		3000	150	75.7575	Ladder Peak
14		7000	150	32.4675	Ladder Peak
15		10380	75	10.94762	Upper Marker

 © Copyright 2003 - 2024 Agilent, Inc.

Assay Class: High Sensitivity DNA Assay
Data Path: C:\...gh Sensitivity DNA Assay_DE23101764_2024-10-31_13-02-48.xad

Created: 10/31/2024 1:02:48 PM
Modified: 10/31/2024 1:44:09 PM

Electropherogram Summary Continued ...

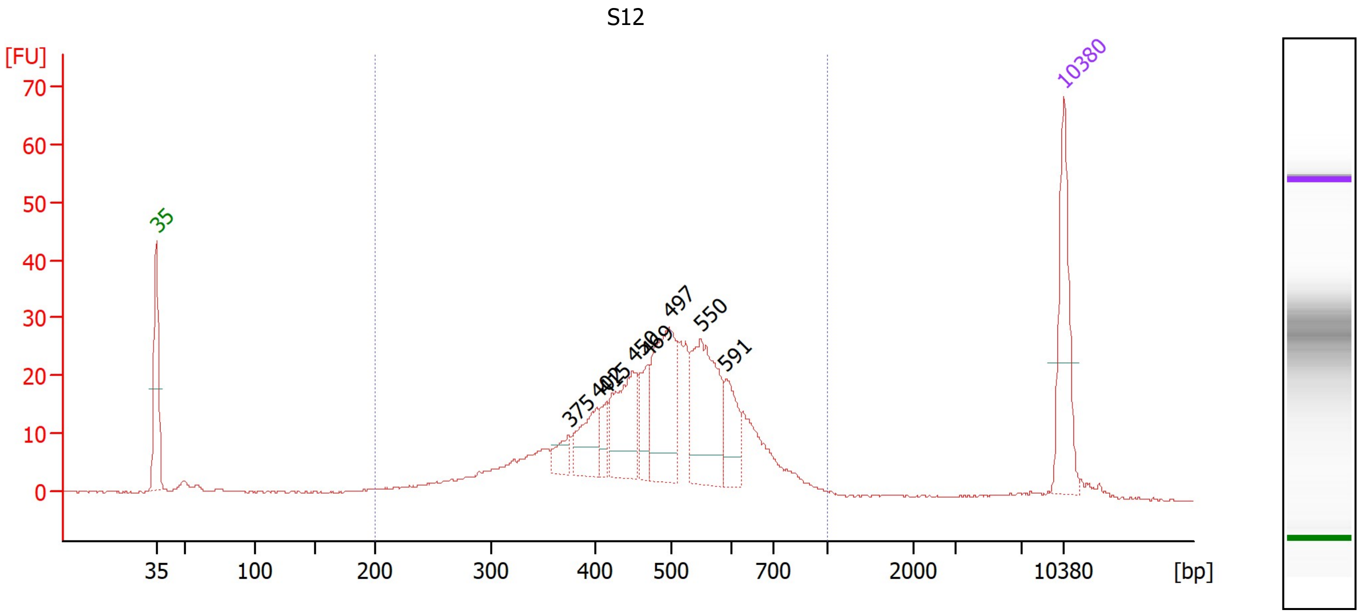

Overall Results for sample 1 : S12

Number of peaks found:	8	Corr. Area 1:	485.4
Noise:	0.2		

Peak table for sample 1 : S12

	Peak	Size [bp]	Conc. [pg/µl]	Molarity [pmol/l]	Observations
1	◀	35	125	5411.25	Lower Marker
2		375.3846	20.37513	82.23932	
3		402.1329	49.50355	186.5187	
4		414.8424	17.82949	65.11955	
5		449.9806	85.62513	288.3123	
6		469.4187	34.39651	111.0222	
7		497.0807	121.4249	370.1148	
8		549.9023	123.4555	340.1579	
9		591.2564	45.24001	115.9318	
10	▶	10380	75	10.94762	Upper Marker

Region table for sample 1 : S12

From [bp]	To [bp]	Corr. Area	% of Total	Average Size [bp]	Size distribution in CV [%]	Conc. [pg/µl]	Molarity [pmol/l]	Color
200	1000	485.432	93.86414	490.5162	24.35281	908.5567	3086.16	

 © Copyright 2003 - 2024 Agilent, Inc.

Assay Class: High Sensitivity DNA Assay
Data Path: C:\...gh Sensitivity DNA Assay_DE23101764_2024-10-31_13-02-48.xad

Created: 10/31/2024 1:02:48 PM
Modified: 10/31/2024 1:44:09 PM

Electropherogram Summary Continued ...

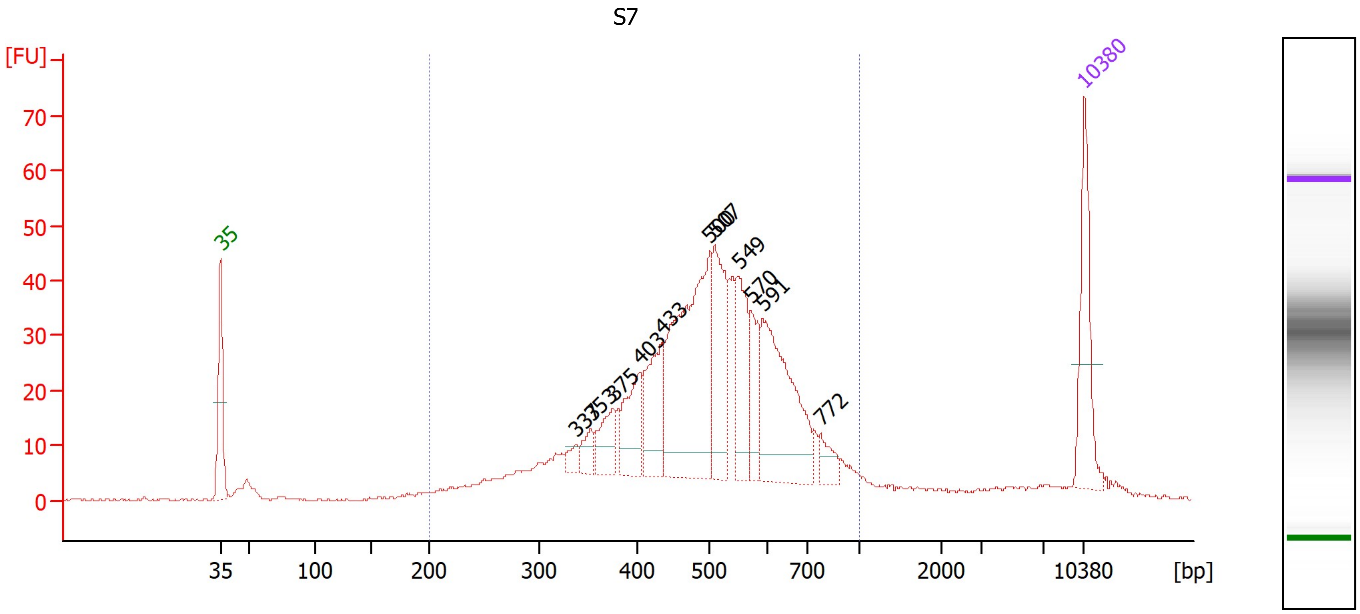

Overall Results for sample 2 : S7

Number of peaks found: 11
Corr. Area 1: 766.2
Noise: 0.2

Peak table for sample 2 : S7

	Peak	Size [bp]	Conc. [pg/µl]	Molarity [pmol/l]	Observations
1	◀	35	125	5411.25	Lower Marker
2		336.9736	14.39321	64.71684	
3		352.5608	22.18625	95.34667	
4		375.1354	45.10294	182.1682	
5		402.7561	70.16253	263.9482	
6		432.9735	89.56874	313.4374	
7		500.0524	292.6422	886.7009	
8		507.4646	122.2775	365.0872	
9		549.1584	87.87109	242.4398	
10		570.4685	51.15251	135.8598	
11		590.8522	176.8667	453.5476	
12		772.3745	21.93586	43.03109	
13	▶	10380	75	10.94762	Upper Marker

Region table for sample 2 : S7

From [bp]	To [bp]	Corr. Area	% of Total	Average Size [bp]	Size distribution in CV [%]	Conc. [pg/µl]	Molarity [pmol/l]	Color
200	1000	766.1951	93.48737	502.6078	25.94033	1430.077	4785.417	

© Copyright 2003 - 2024 Agilent, Inc.

Assay Class: High Sensitivity DNA Assay
Data Path: C:\...gh Sensitivity DNA Assay_DE23101764_2024-10-31_13-02-48.xad

Created: 10/31/2024 1:02:48 PM
Modified: 10/31/2024 1:44:09 PM

Electropherogram Summary Continued ...

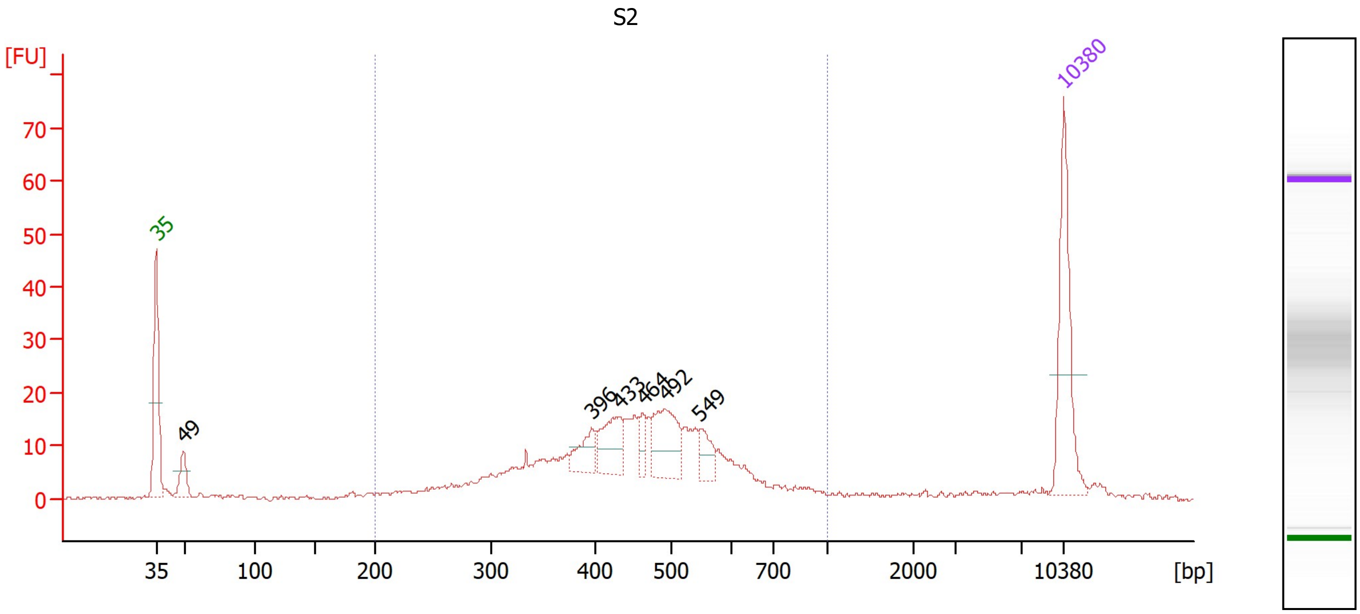

Overall Results for sample 3 : **S2**

Number of peaks found: 6
Corr. Area 1: 319.2
Noise: 0.4

Peak table for sample 3 : **S2**

	Peak	Size [bp]	Conc. [pg/µl]	Molarity [pmol/l]	Observations
1	◀	35	125	5411.25	Lower Marker
2		48.95834	29.20356	903.7844	
3		396.3415	25.96864	99.27396	
4		433.1838	43.84159	153.345	
5		463.8939	14.44782	47.18884	
6		492.357	60.7312	186.8906	
7		548.7728	21.54925	59.49703	
8	▶	10380	75	10.94762	Upper Marker

Region table for sample 3 : **S2**

From [bp]	To [bp]	Corr. Area	% of Total	Average Size [bp]	Size distribution in CV [%]	Conc. [pg/µl]	Molarity [pmol/l]	Color
200	1000	319.2066	87.49992	459.7696	26.16611	558.7609	2034.198	

© Copyright 2003 - 2024 Agilent, Inc.

Assay Class: High Sensitivity DNA Assay
Data Path: C:\...gh Sensitivity DNA Assay_DE23101764_2024-10-31_13-02-48.xad

Created: 10/31/2024 1:02:48 PM
Modified: 10/31/2024 1:44:09 PM

Electropherogram Summary Continued ...

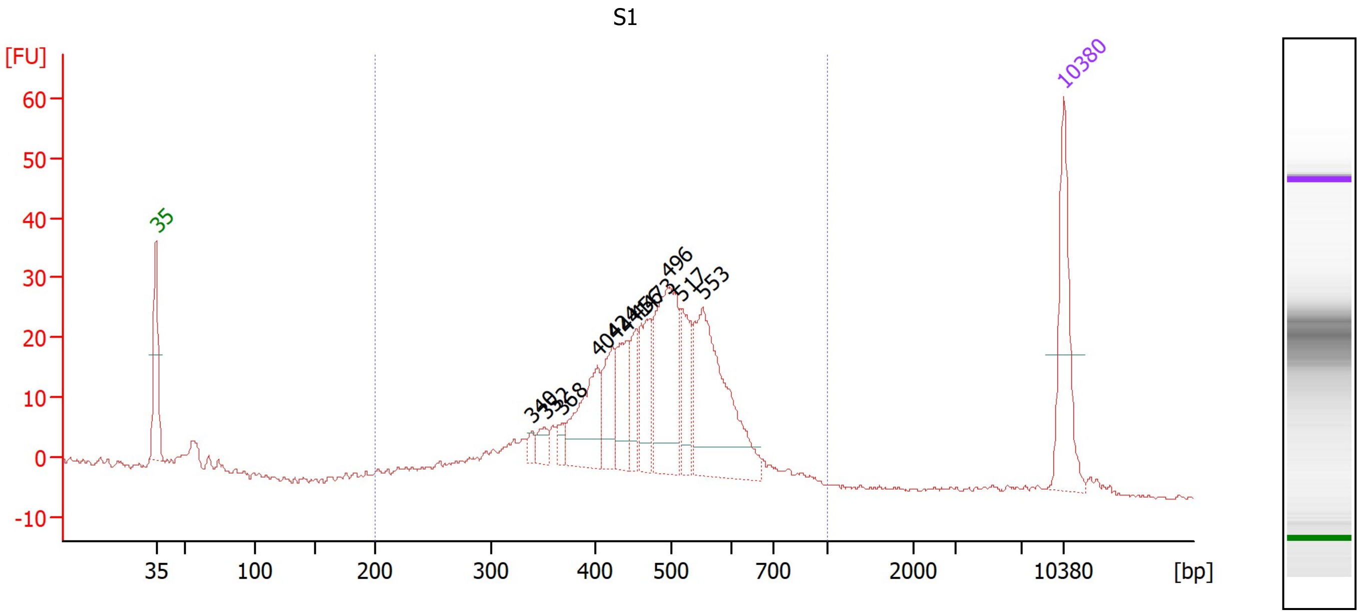

Overall Results for sample 4 : S1

Number of peaks found: 11
Corr. Area 1: 480.0
Noise: 0.5

Peak table for sample 4 : S1

	Peak	Size [bp]	Conc. [pg/µl]	Molarity [pmol/l]	Observations
1	◀	35	125	5411.25	Lower Marker
2		339.7441	8.793749	39.21731	
3		351.5256	16.0254	69.07288	
4		368.3564	11.78887	48.49081	
5		403.5362	81.35011	305.4438	
6		423.5376	48.45097	173.327	
7		441.2311	51.57198	177.0938	
8		455.8474	35.04535	116.4841	
9		472.7717	59.00302	189.0943	
10		495.8502	145.1215	443.4422	
11		517.0264	52.23368	153.0712	
12		552.809	177.7552	487.1951	
13	▶	10380	75	10.94762	Upper Marker

Region table for sample 4 : S1

From [bp]	To [bp]	Corr. Area	% of Total	Average Size [bp]	Size distribution in CV [%]	Conc. [pg/µl]	Molarity [pmol/l]	Color
200	1000	480.0135	92.89803	478.2554	21.54588	913.2794	3106.028	

© Copyright 2003 - 2024 Agilent, Inc.

Assay Class: High Sensitivity DNA Assay
Data Path: C:\...gh Sensitivity DNA Assay_DE23101764_2024-10-31_13-02-48.xad

Created: 10/31/2024 1:02:48 PM
Modified: 10/31/2024 1:44:09 PM

Electropherogram Summary Continued ...

S10

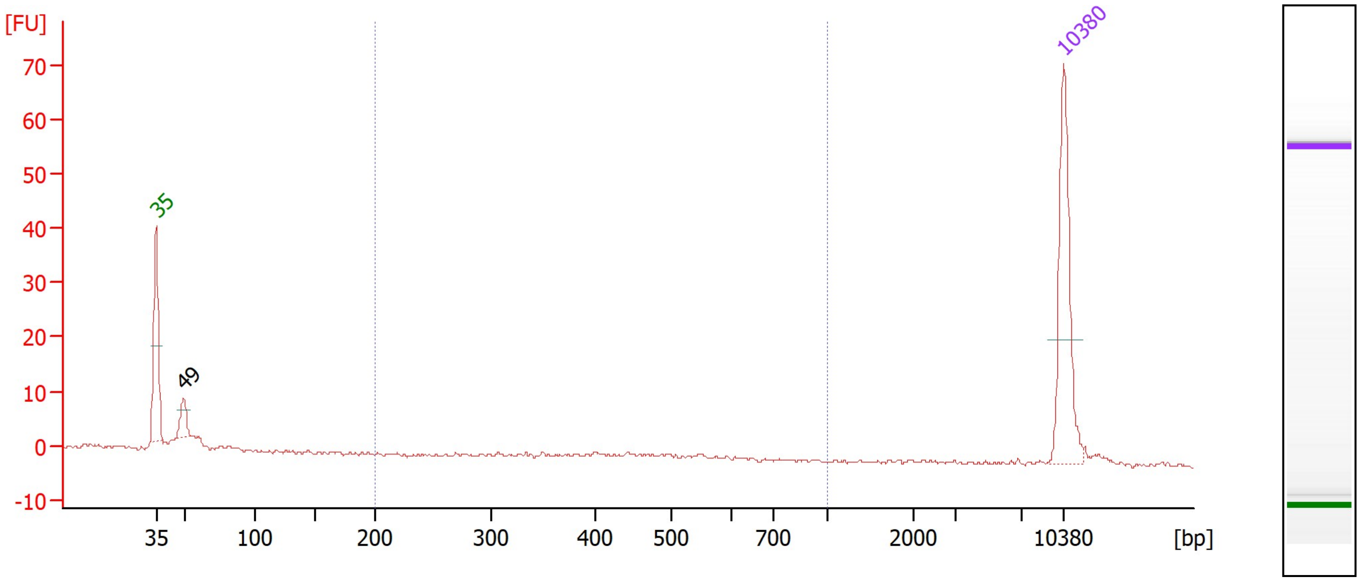

Overall Results for sample 5 : S10

Number of peaks found: 1
Corr. Area 1: 4.3
Noise: 0.3

Peak table for sample 5 : S10

	Peak	Size [bp]	Conc. [pg/µl]	Molarity [pmol/l]	Observations
1	◀	35	125	5411.25	Lower Marker
2		49.03679	22.94709	709.0243	
3	▶	10380	75	10.94762	Upper Marker

Region table for sample 5 : S10

From [bp]	To [bp]	Corr. Area	% of Total	Average Size [bp]	Size distribution in CV [%]	Conc. [pg/µl]	Molarity [pmol/l]	Color
200	1000	4.269529	10.74559	458.4943	15.72878	7.301662	24.93028	

© Copyright 2003 - 2024 Agilent, Inc.

Assay Class: High Sensitivity DNA Assay
Data Path: C:\...gh Sensitivity DNA Assay_DE23101764_2024-10-31_13-02-48.xad

Created: 10/31/2024 1:02:48 PM
Modified: 10/31/2024 1:44:09 PM

Electropherogram Summary Continued ...

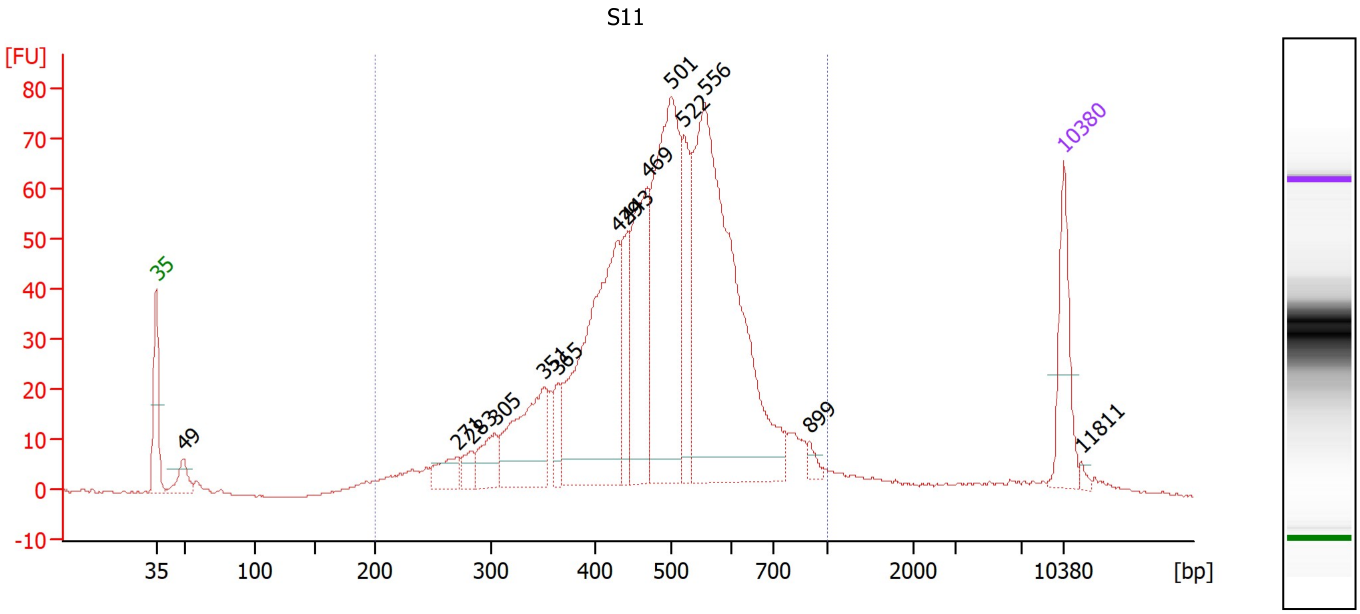

Overall Results for sample 6 : S11

Number of peaks found: 14

Corr. Area 1: 1,247.1

Noise: 0.3

Peak table for sample 6 : S11

	Peak	Size [bp]	Conc. [pg/µl]	Molarity [pmol/l]	Observations
1	◄	35	125	5411.25	Lower Marker
2		48.78936	41.76887	1297.129	
3		270.7039	43.28623	242.2763	
4		283.4268	23.19249	123.983	
5		304.5546	52.61108	261.7385	
6		351.0617	164.6829	710.7562	
7		364.6017	34.17405	142.0147	
8		428.9553	382.1618	1349.867	
9		442.6782	81.99099	280.6297	
10		469.3168	206.1889	665.6637	
11		501.0039	407.1625	1231.352	
12		522.3148	110.69	321.0935	
13		555.8033	627.2196	1709.835	
14		899.3568	10.94729	18.44294	
15	►	10380	75	10.94762	Upper Marker
16		11810.74	0	0	

Region table for sample 6 : S11

From [bp]	To [bp]	Corr. Area	% of Total	Average Size [bp]	Size distribution in CV [%]	Conc. [pg/µl]	Molarity [pmol/l]	Color
200	1000	1247.118	93.25375	492.3919	24.87135	2432.838	8252.693	

© Copyright 2003 - 2024 Agilent, Inc.

Assay Class: High Sensitivity DNA Assay
Data Path: C:\...gh Sensitivity DNA Assay_DE23101764_2024-10-31_13-02-48.xad

Created: 10/31/2024 1:02:48 PM
Modified: 10/31/2024 1:44:09 PM

Electropherogram Summary Continued ...

S8

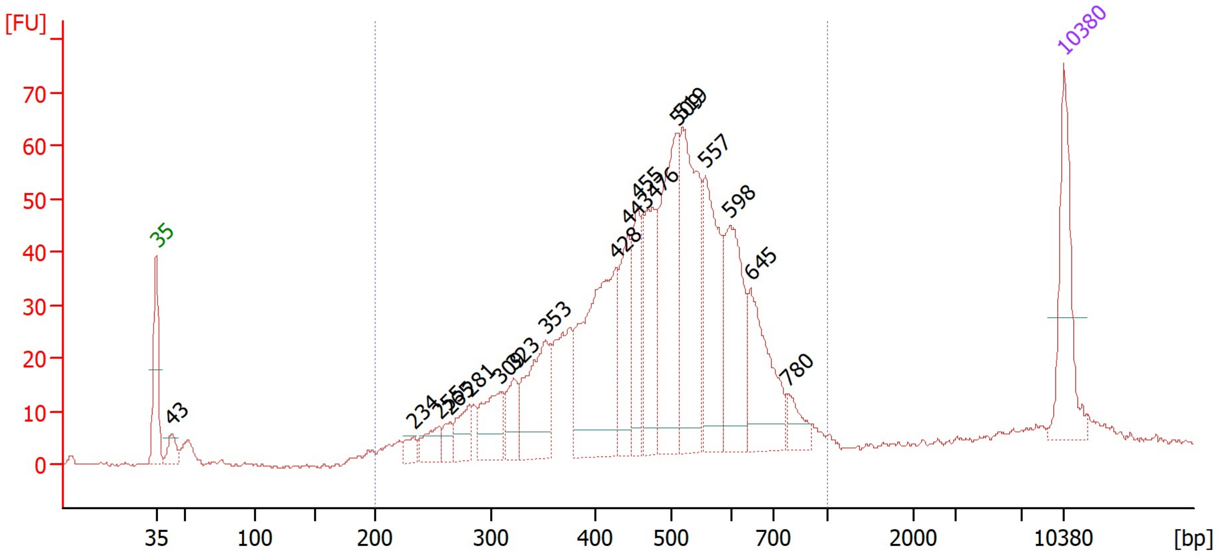

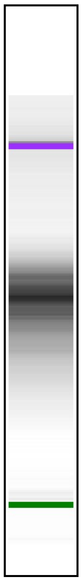

Overall Results for sample 7 : S8

Number of peaks found: 18

Corr. Area 1: 1,008.4

Noise: 0.3

Peak table for sample 7 : S8

	Peak	Size [bp]	Conc. [pg/µl]	Molarity [pmol/l]	Observations
1	◀	35	125	5411.25	Lower Marker
2		43.00802	25.70107	905.4352	
3		233.6395	15.81617	102.5677	
4		255.3129	30.19772	179.2079	
5		265.0658	17.09717	97.7296	
6		281.3209	35.5676	191.5615	
7		308.7406	60.7559	298.1606	
8		322.5798	37.94815	178.2416	
9		352.6652	105.4495	453.0413	
10		428.3264	213.3845	754.8204	
11		443.1777	81.77209	279.5651	
12		454.7286	75.00856	249.9276	
13		476.1804	98.3669	312.9919	
14		509.4699	172.709	513.6321	
15		518.8049	191.9773	560.6626	
16		557.1824	129.1551	351.2123	
17		597.6341	120.1191	304.5316	
18		645.2105	102.0675	239.6855	
19		780.0038	22.81094	44.31004	
20	▶	10380	75	10.94762	Upper Marker

Region table for sample 7 : S8

From [bp]	To [bp]	Corr. Area	% of Total	Average Size [bp]	Size distribution in CV [%]	Conc. [pg/µl]	Molarity [pmol/l]	Color
200	1000	1008.37	93.71263	487.7604	25.5042	1649.43	5687.248	

 © Copyright 2003 - 2024 Agilent, Inc.

Assay Class: High Sensitivity DNA Assay
Data Path: C:\...gh Sensitivity DNA Assay_DE23101764_2024-10-31_13-02-48.xad

Created: 10/31/2024 1:02:48 PM
Modified: 10/31/2024 1:44:09 PM

Electropherogram Summary Continued ...

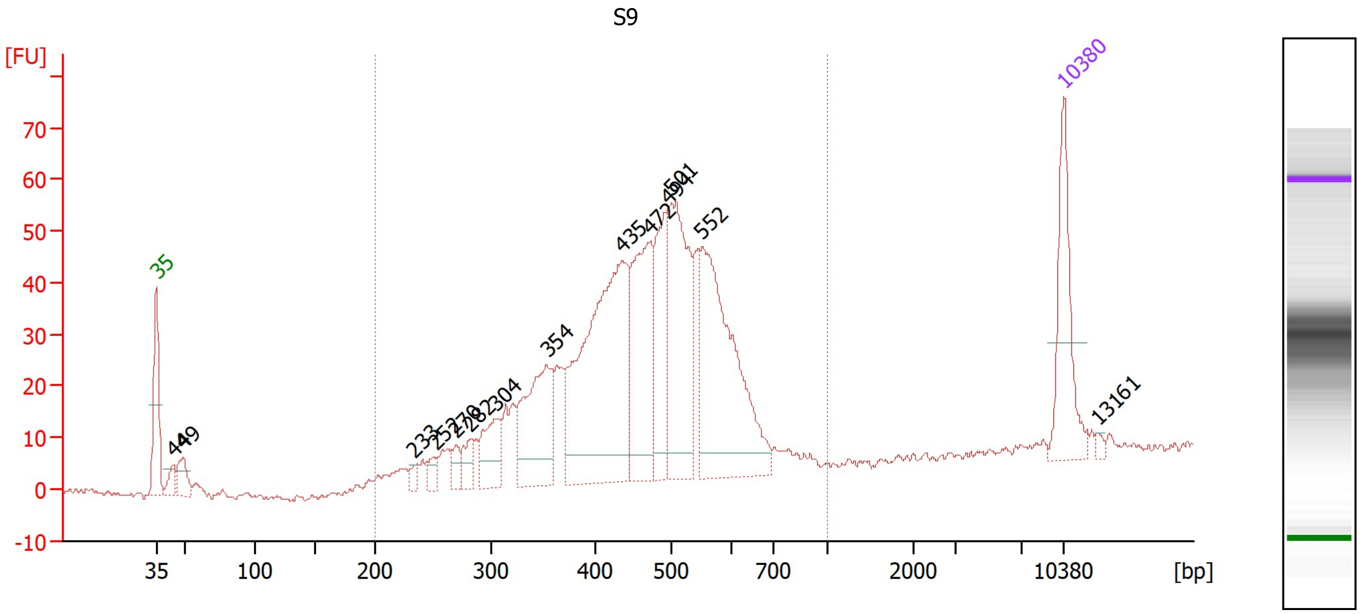

Overall Results for sample 8 : S9

Number of peaks found: 14

Corr. Area 1: 796.3

Noise: 0.4

Peak table for sample 8 : S9

	Peak	Size [bp]	Conc. [pg/µl]	Molarity [pmol/l]	Observations
1	◀	35	125	5411.25	Lower Marker
2		43.50452	19.22436	669.5349	
3		48.7381	30.00359	932.7393	
4		233.3712	9.356731	60.74808	
5		251.6301	14.42494	86.85748	
6		269.889	13.96326	78.3894	
7		282.0614	21.31278	114.4859	
8		304.0424	51.75268	257.9018	
9		353.8116	125.2699	536.451	
10		435.2411	318.2809	1107.991	
11		472.3123	152.3046	488.5842	
12		494.2178	94.12553	288.5657	
13		501.2043	177.5913	536.8619	
14		552.0446	228.6498	627.5557	
15	▶	10380	75	10.94762	Upper Marker
16		13160.63	0	0	

Region table for sample 8 : S9

From [bp]	To [bp]	Corr. Area	% of Total	Average Size [bp]	Size distribution in CV [%]	Conc. [pg/µl]	Molarity [pmol/l]	Color
200	1000	796.3391	97.13133	462.9696	20.99015	1227.776	4318.165	

© Copyright 2003 - 2024 Agilent, Inc.

Assay Class: High Sensitivity DNA Assay
Data Path: C:\...gh Sensitivity DNA Assay_DE23101764_2024-10-31_13-02-48.xad

Created: 10/31/2024 1:02:48 PM
Modified: 10/31/2024 1:44:09 PM

Electropherogram Summary Continued ...

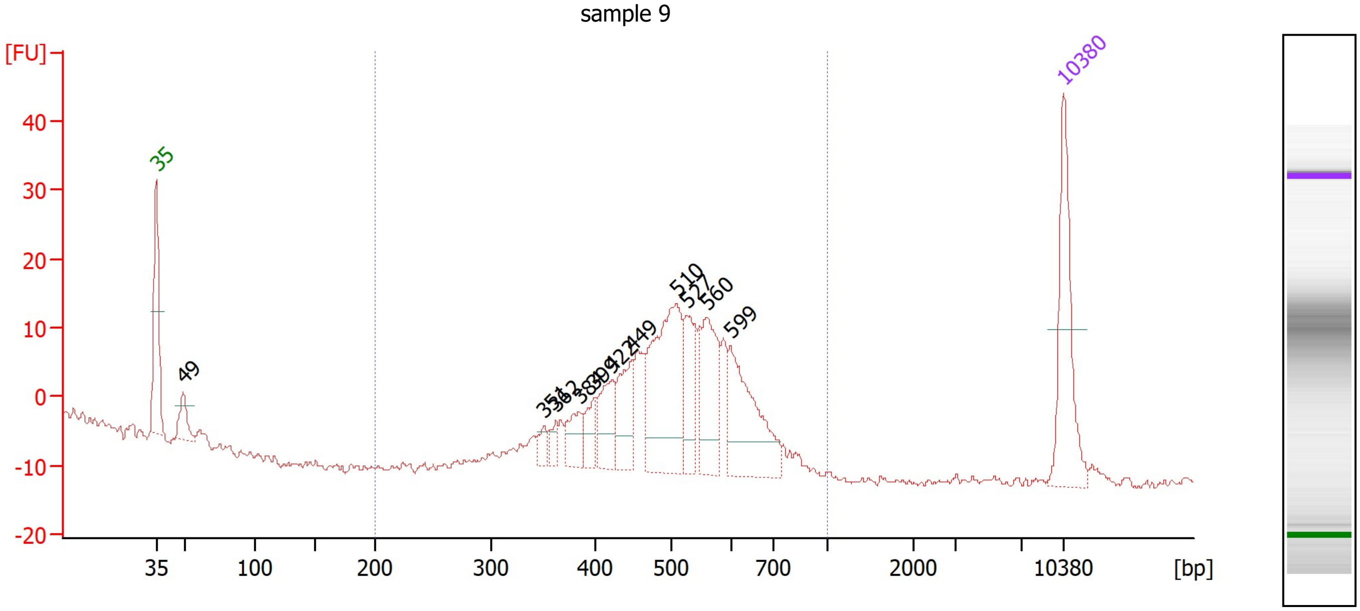

Overall Results for sample 9 : **sample 9**

Number of peaks found: 11

Corr. Area 1: 227.8

Noise: 0.6

Peak table for sample 9 : **sample 9**

	Peak	Size [bp]	Conc. [pg/µl]	Molarity [pmol/l]	Observations
1	◀	35	125	5411.25	Lower Marker
2		48.72061	28.62257	890.1262	
3		350.5214	10.77031	46.5553	
4		362.4652	10.36576	43.33018	
5		384.4669	27.23568	107.3334	
6		398.9253	20.92274	79.46625	
7		421.7997	41.86191	150.3725	
8		448.5211	46.42776	156.8377	
9		510.0536	145.7938	433.0908	
10		527.3917	45.74562	131.4232	
11		559.9004	71.60696	193.776	
12		598.9111	93.5057	236.5546	
13	▶	10380.01	75	10.94761	Upper Marker

Region table for sample 9 : **sample 9**

From [bp]	To [bp]	Corr. Area	% of Total	Average Size [bp]	Size distribution in CV [%]	Conc. [pg/µl]	Molarity [pmol/l]	Color
200	1000	227.7848	97.30797	517.0114	14.73112	437.4148	1320.164	

 © Copyright 2003 - 2024 Agilent, Inc.

Assay Class: High Sensitivity DNA Assay
Data Path: C:\...gh Sensitivity DNA Assay_DE23101764_2024-10-31_13-02-48.xad

Created: 10/31/2024 1:02:48 PM
Modified: 10/31/2024 1:44:09 PM

Electropherogram Summary Continued ...

sample 10

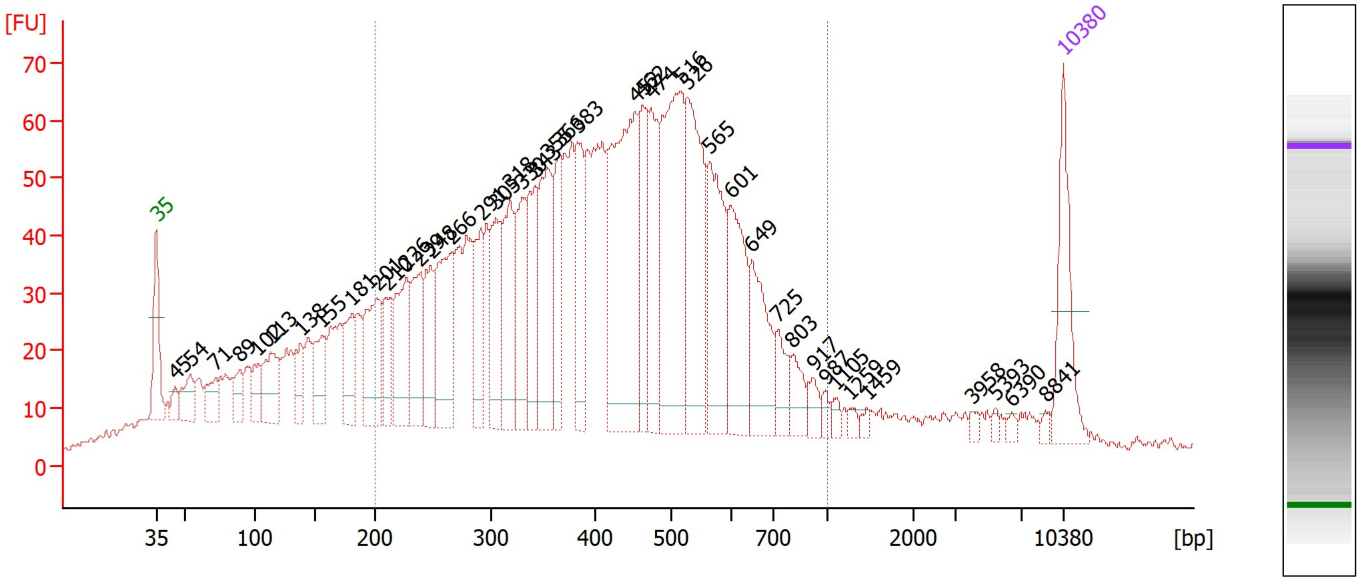

Overall Results for sample 10 : **sample 10**

Number of peaks found: 42

Corr. Area 1: 1,859.1

Noise: 0.7

Peak table for sample 10 : **sample 10**

	Peak	Size [bp]	Conc. [pg/µl]	Molarity [pmol/l]	Observations
1	◀	35	125	5411.25	Lower Marker
2		44.77534	21.09214	713.7355	
3		53.52293	43.67932	1236.493	
4		71.37165	42.16753	895.1752	
5		88.75067	32.72972	558.7614	
6		101.6806	37.41927	557.5872	
7		112.7006	69.14607	929.6021	
8		138.0465	39.14599	429.6528	
9		155.1772	63.94136	624.3235	
10		180.7691	62.51936	524.0176	
11		201.3876	112.0704	843.1675	
12		209.9404	56.35189	406.6943	
13		225.9057	113.2136	759.3241	
14		239.02	87.95718	557.5613	
15		248.143	76.20599	465.3104	
16		265.8188	124.8543	711.6614	
17		291.4773	69.95607	363.644	
18		305.0988	82.88629	411.6212	
19		318.3958	105.5122	502.1008	
20		329.7933	96.24716	442.1826	
21		343.0903	98.33172	434.251	
22		355.121	124.9712	533.1991	
23		365.8852	76.14258	315.3105	
24		382.9813	83.56865	330.6142	
25		452.2006	278.8048	934.1675	
26		461.7512	87.32046	286.5257	

© Copyright 2003 - 2024 Agilent, Inc.

Assay Class: High Sensitivity DNA Assay
Data Path: C:\...gh Sensitivity DNA Assay_DE23101764_2024-10-31_13-02-48.xad
Created: 10/31/2024 1:02:48 PM
Modified: 10/31/2024 1:44:09 PM

Electropherogram Summary Continued ...

... Peak table for sample 10 : sample 10

Peak		Size [bp]	Conc. [pg/µl]	Molarity [pmol/l]	Observations
27		473.9066	103.0262	329.39	
28		516.3145	234.9913	689.5935	
29		526.1379	171.2639	493.1988	
30		565.4318	128.0185	343.0425	
31		600.5107	107.7512	271.8672	
32		648.522	88.71666	207.2699	
33		724.9974	32.62273	68.17725	
34		802.6348	26.57913	50.17397	
35		917.2422	15.64638	25.84553	
36		987.4861	8.028349	12.3183	
37		1104.724	7.561401	10.3706	
38		1258.56	5.697617	6.859225	
39		1458.547	5.271559	5.476138	
40		3958.244	4.657464	1.7828	
41		5393.332	4.15892	1.168367	
42		6389.921	4.810107	1.140552	
43		8841.123	4.68002	0.8020399	
44	▶	10380	75	10.94762	Upper Marker

Region table for sample 10 : sample 10

From [bp]	To [bp]	Corr. Area	% of Total	Average Size [bp]	Size distribution in CV [%]	Conc. [pg/µl]	Molarity [pmol/l]	Color
200	1000	1859.122	71.10992	430.9421	34.33787	3488.008	14509.71	

© Copyright 2003 - 2024 Agilent, Inc.

Assay Class: High Sensitivity DNA Assay
Data Path: C:\...gh Sensitivity DNA Assay_DE23101764_2024-10-31_13-02-48.xad

Created: 10/31/2024 1:02:48 PM
Modified: 10/31/2024 1:44:09 PM

Electropherogram Summary Continued ...

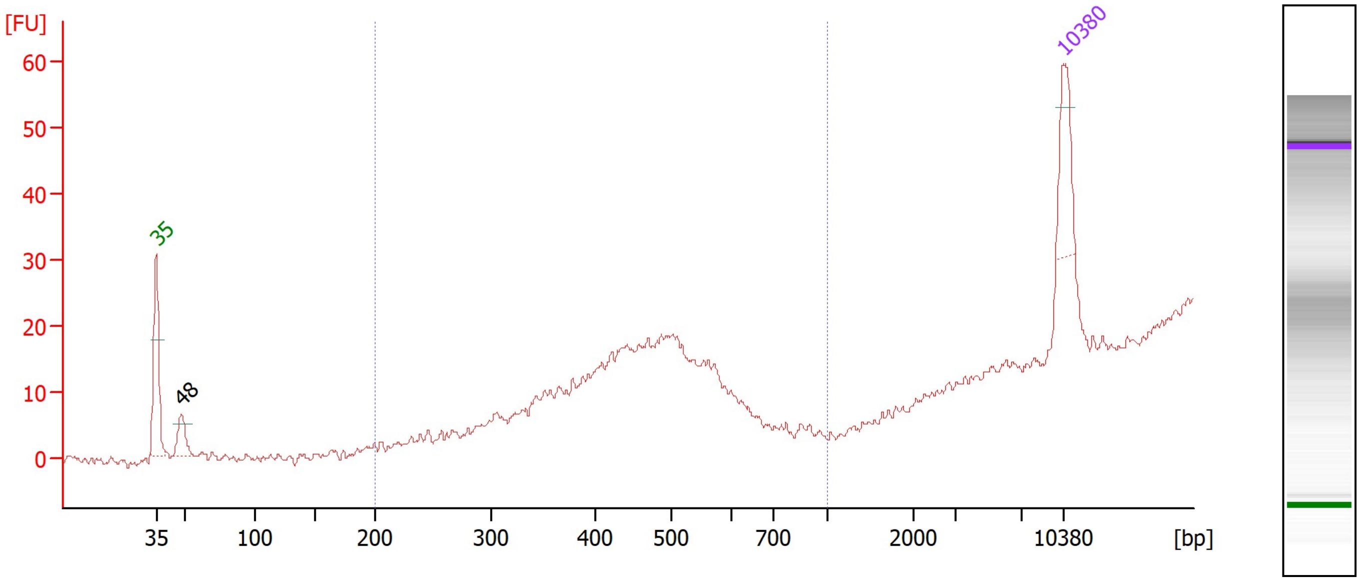

Overall Results for sample 11 : sample 11

Number of peaks found: 1
Corr. Area 1: 0.0
Noise: 0.9

Peak table for sample 11 : sample 11

	Peak	Size [bp]	Conc. [pg/µl]	Molarity [pmol/l]	Observations
1	◀	35	125	5411.25	Lower Marker
2		48.08488	63.64803	2005.543	
3	▶	10380	75	10.94762	Upper Marker

Region table for sample 11 : sample 11

From [bp]	To [bp]	Corr. Area	% of Total	Average Size [bp]	Size distribution in CV [%]	Conc. [pg/µl]	Molarity [pmol/l]	Color
200	1000	0	0	0	0	0	0	

© Copyright 2003 - 2024 Agilent, Inc.

Assay Class:	High Sensitivity DNA Assay	Created:	10/31/2024 1:02:48 PM
Data Path:	C:\...gh Sensitivity DNA Assay_DE23101764_2024-10-31_13-02-48.xad	Modified:	10/31/2024 1:44:09 PM

Gel Image

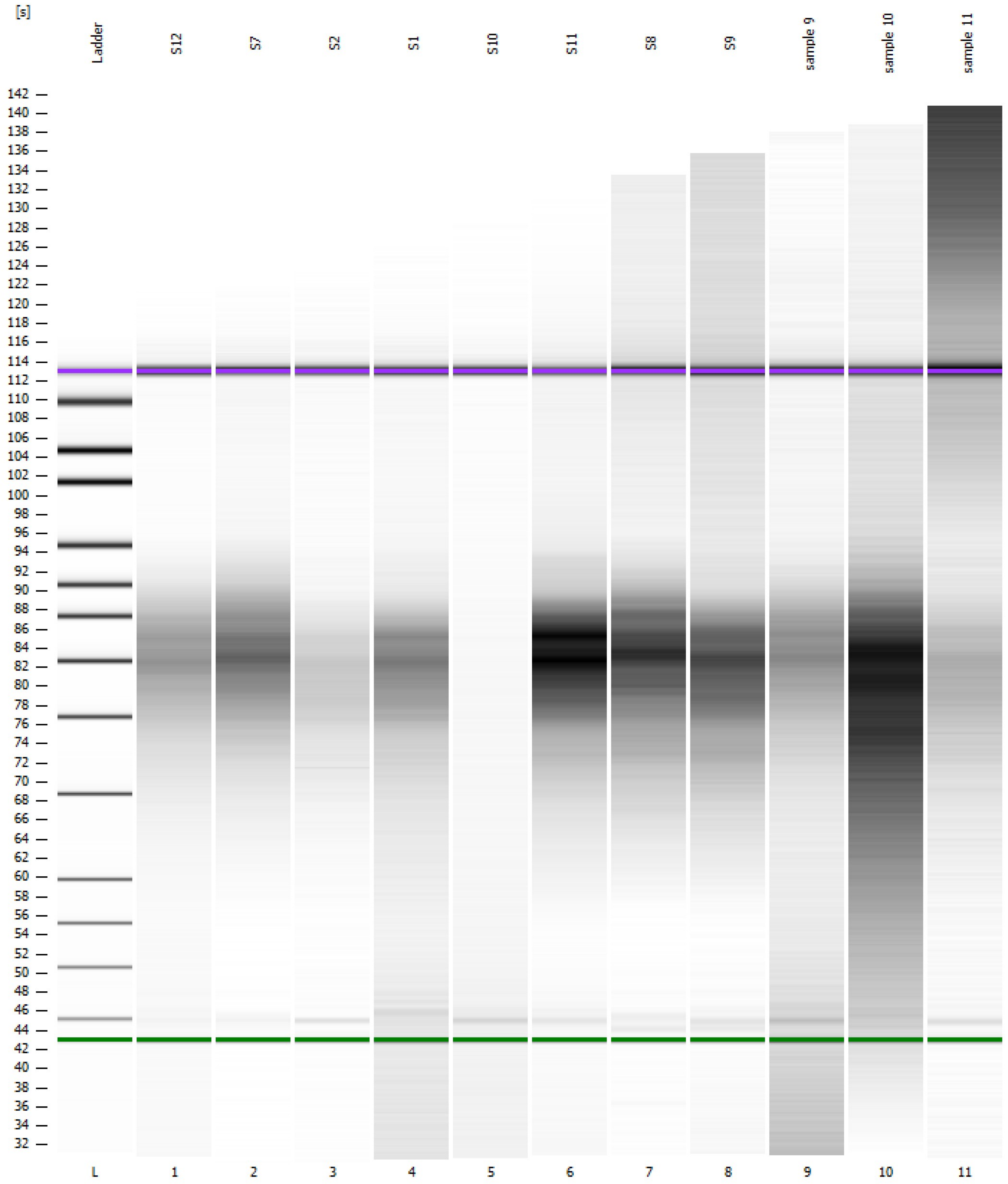

 © Copyright 2003 - 2024 Agilent, Inc.
