# Supplementary material for: DMS-informed secondary structure modeling of Epstein–Barr Virus LMP-1 pre-mRNA defines novel elements spanning introns
Source: PLoS One. 2026 Jul 2;21(7):e0345208. doi: 10.1371/journal.pone.0345208 (PMC13327190; doi:10.1371/journal.pone.0345208)
Supplement: S3 Appendix — This appendix serves as a guide for the Illumina DNA library preparation and pooling process. It includes the Bioanalyzer results used for fragment size analysis to ensure a normalized distribution of DNA fragment sizes prior to sequencing on the iSeq100. These quality control steps were necessary to confirm the average fragment size for each of the sixteen individual libraries. (ZIP) [file pone.0345208.s013.zip › S3_Appendix/S3_Bioanalyzer_Library_B.pdf]

Assay Class: High Sensitivity DNA Assay
Data Path: C:\...gh Sensitivity DNA Assay_DE23101764_2024-11-07_11-35-32.xad

Created: 11/7/2024 11:35:31 AM
Modified: 11/7/2024 12:16:58 PM

Electrophoresis File Run Summary

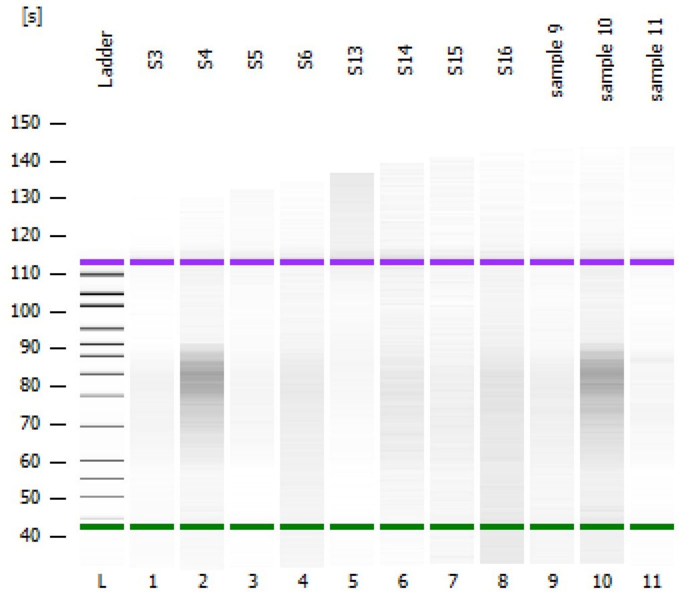

<u>Instrument Information:</u>

Instrument Name: DE23101764
Firmware: C.01.069
Serial#: DE23101764
Type: G2938B

<u>Assay Information:</u>

Assay Origin Path: C:\Program Files (x86)\Agilent\2100 bioanalyzer\2100 expert\assays\dsDNA\High Sensitivity DNA.xsy

Assay Class: High Sensitivity DNA Assay

Version: 1.03

Assay Comments: Copyright © 2003-2010 Agilent Technologies

<u>Chip Information:</u>

Chip Lot #: CU19BK50

Reagent Kit Lot #: 2236

Chip Comments:

S3

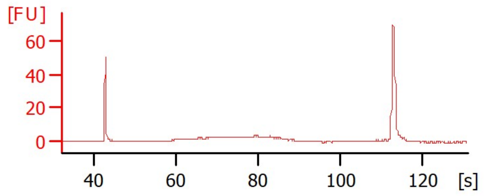

S4

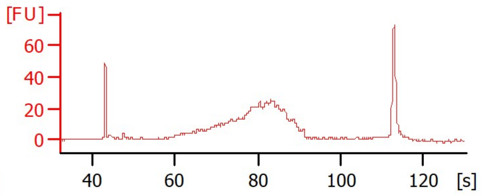

S5

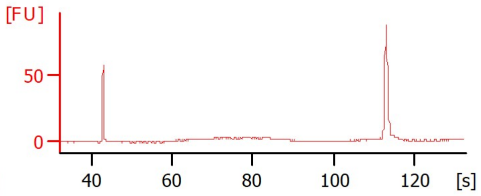

S6

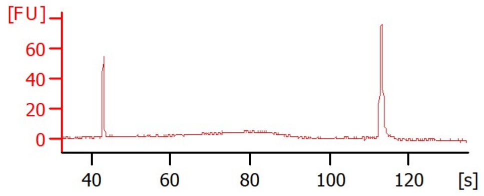

S13

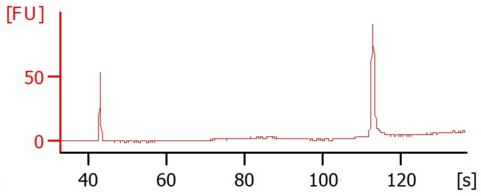

S14

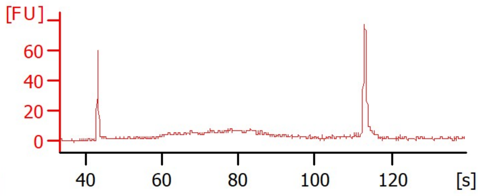

S15

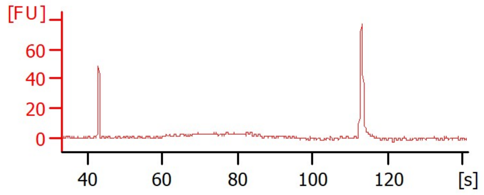

S16

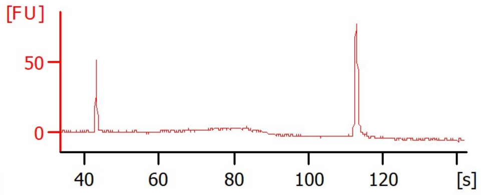

sample 9

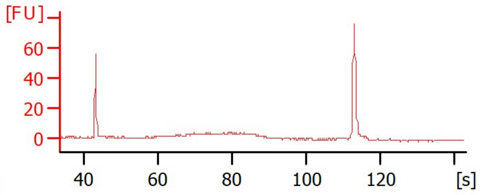

sample 10

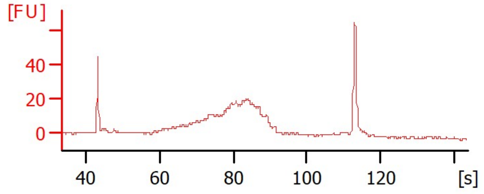

sample 11

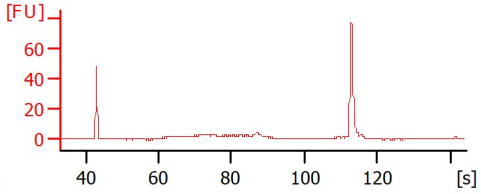

© Copyright 2003 - 2024 Agilent, Inc.

Assay Class: High Sensitivity DNA Assay
Data Path: C:\...gh Sensitivity DNA Assay_DE23101764_2024-11-07_11-35-32.xad
Created: 11/7/2024 11:35:31 AM
Modified: 11/7/2024 12:16:58 PM

Electrophoresis File Run Summary (Chip Summary)

Sample Name	Sample Comment	Rest. Digest	Status	Observation	Result Label	Result Color
S3		☐	✓			
S4		☐	✓			
S5		☐	✓			
S6		☐	✓			
S13		☐	✓			
S14		☐	✓			
S15		☐	✓			
S16		☐	✓			
sample 9		☐	✓			
sample 10		☐	✓			
sample 11		☐	✓			
Ladder		☐	✓			

Chip Lot #

CU19BK50

Reagent Kit Lot #

2236

Chip Comments :

© Copyright 2003 - 2024 Agilent, Inc.

Assay Class: High Sensitivity DNA Assay
Data Path: C:\...gh Sensitivity DNA Assay_DE23101764_2024-11-07_11-35-32.xad
Created: 11/7/2024 11:35:31 AM
Modified: 11/7/2024 12:16:58 PM

Electrophoresis Assay Details

General Analysis Settings

Number of Available Sample and Ladder Wells (Max.) : 12
Minimum Visible Range [s] : 32
Maximum Visible Range [s] : 138
Start Analysis Time Range [s] : 33
End Analysis Time Range [s] : 137.5
Ladder Concentration [pg/µl] : 1950
Uses Standard Area for Ladder Fragments
Lower Marker Concentration [pg/µl] : 125
Upper Marker Concentration [pg/µl] : 75
Used Upper Marker for Quantitation
Standard Curve Fit is Point to Point
Show Data Aligned to Lower and Upper Marker

Integrator Settings

Integration Start Time [s] : 33.05
Integration End Time [s] : 137
Slope Threshold : 0.8
Height Threshold [FU] : 5
Area Threshold : 0.1
Width Threshold [s] : 0.6
Baseline Plateau [s] : 0.5

Filter Settings

Filter Width [s] : 0.5
Polynomial Order : 4

Ladder

	**Size**	**Area**
1	35	160.321
2	50	210.177
3	100	208.32
4	150	221.333
5	200	242.044
6	300	270.153
7	400	304.774
8	500	306.312
9	600	336.215
10	700	321.09
11	1000	365.785
12	2000	412.721
13	3000	411.427
14	7000	399.508
15	10380	213.613

© Copyright 2003 - 2024 Agilent, Inc.

Assay Class: High Sensitivity DNA Assay
Data Path: C:\...gh Sensitivity DNA Assay_DE23101764_2024-11-07_11-35-32.xad

Created: 11/7/2024 11:35:31 AM
Modified: 11/7/2024 12:16:58 PM

Electropherogram Summary

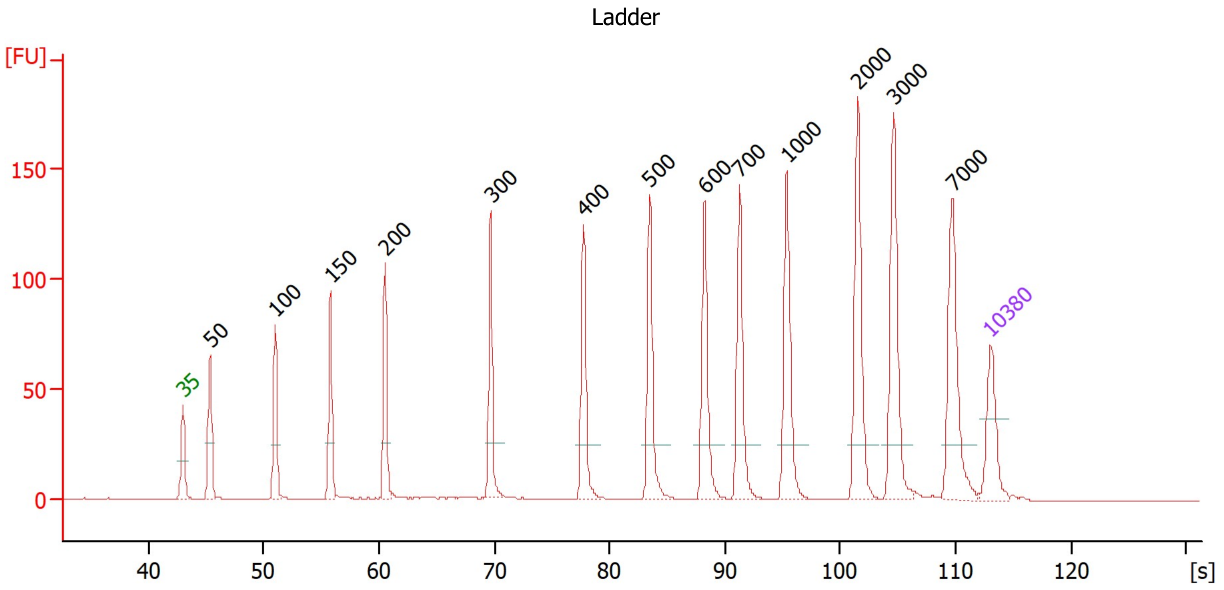

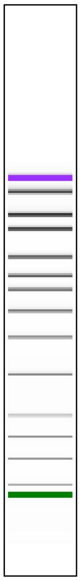

Overall Results for Ladder

Noise: 0.1

Peak table for Ladder

	Peak	Size [bp]	Conc. [pg/µl]	Molarity [pmol/l]	Observations
1		35	125	5411.25	Lower Marker
2		50	150	4545.45	Ladder Peak
3		100	150	2272.725	Ladder Peak
4		150	150	1515.15	Ladder Peak
5		200	150	1136.363	Ladder Peak
6		300	150	757.575	Ladder Peak
7		400	150	568.1813	Ladder Peak
8		500	150	454.545	Ladder Peak
9		600	150	378.7875	Ladder Peak
10		700	150	324.675	Ladder Peak
11		1000	150	227.2725	Ladder Peak
12		2000	150	113.6363	Ladder Peak
13		3000	150	75.7575	Ladder Peak
14		7000	150	32.4675	Ladder Peak
15		10380	75	10.94762	Upper Marker

© Copyright 2003 - 2024 Agilent, Inc.

Assay Class: High Sensitivity DNA Assay
Data Path: C:\...gh Sensitivity DNA Assay_DE23101764_2024-11-07_11-35-32.xad
Created: 11/7/2024 11:35:31 AM
Modified: 11/7/2024 12:16:58 PM

Electropherogram Summary Continued ...

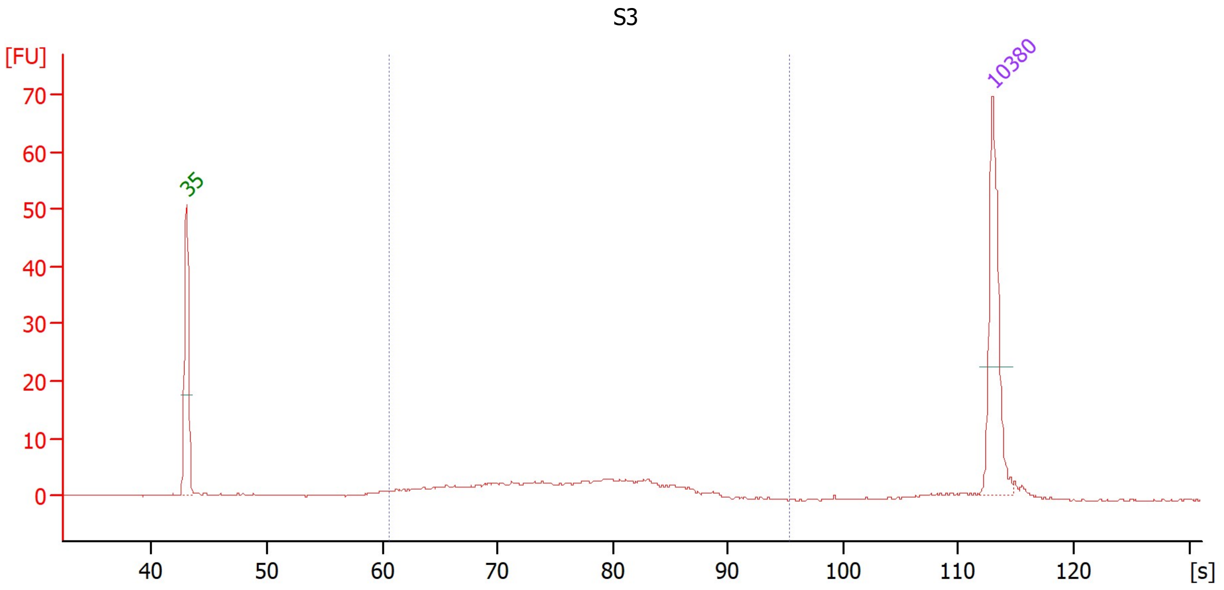

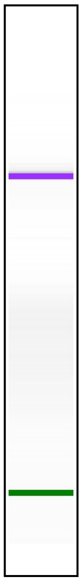

Overall Results for sample 1 : S3

Number of peaks found:	0	Corr. Area 1:	83.3
Noise:	0.1		

Peak table for sample 1 : S3

	Peak	Size [bp]	Conc. [pg/µl]	Molarity [pmol/l]	Observations
1	◀	35	125	5411.25	Lower Marker
2	▶	10380	75	10.94762	Upper Marker

Region table for sample 1 : S3

From [s]	To [s]	Corr. Area	% of Total	Average Size [bp]	Size distribution in CV [%]	Conc. [pg/µl]	Molarity [pmol/l]	Color
60.51165	95.34863	83.31657	81.4432	386.8298	27.01254	155.3187	682.0177	

 © Copyright 2003 - 2024 Agilent, Inc.

Assay Class: High Sensitivity DNA Assay
Data Path: C:\...gh Sensitivity DNA Assay_DE23101764_2024-11-07_11-35-32.xad

Created: 11/7/2024 11:35:31 AM
Modified: 11/7/2024 12:16:58 PM

Electropherogram Summary Continued ...

S4

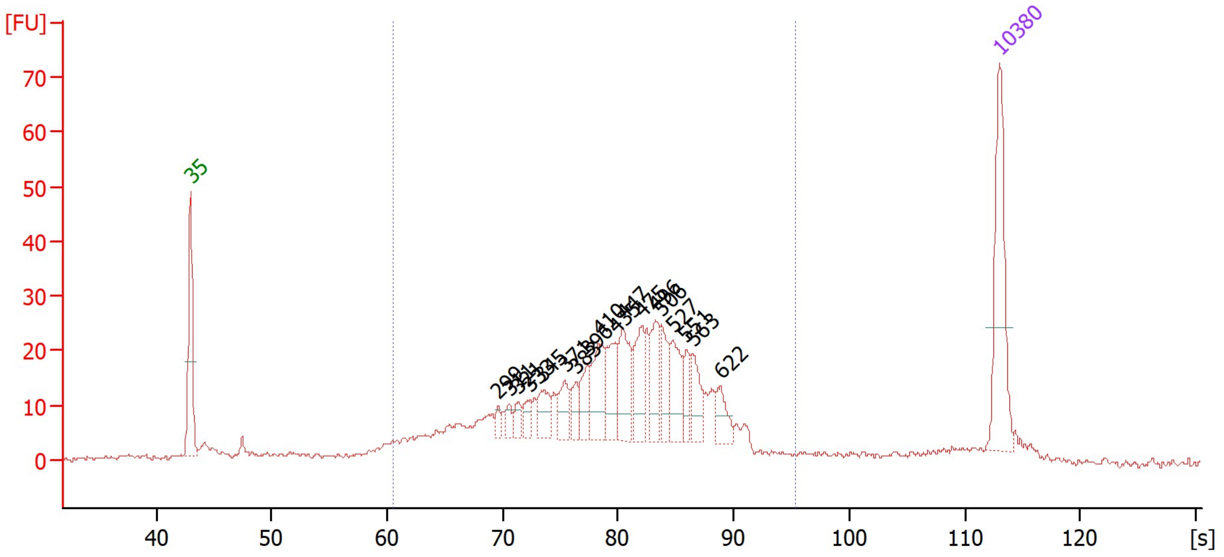

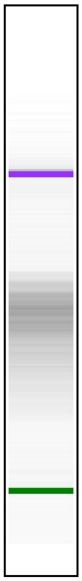

Overall Results for sample 2 : S4

Number of peaks found: 18

Corr. Area 1: 533.5

Noise: 0.4

Peak table for sample 2 : S4

	Peak	Size [bp]	Conc. [pg/µl]	Molarity [pmol/l]	Observations
1	◀	35	125	5411.25	Lower Marker
2		299.4186	8.589692	43.46647	
3		311.4559	9.077199	44.15816	
4		320.6861	11.24644	53.1362	
5		333.3775	12.25025	55.67552	
6		345.492	24.93536	109.3536	
7		370.8749	23.7069	96.85073	
8		382.9894	16.27939	64.40314	
9		395.6809	24.78305	94.89979	
10		410.071	45.53181	168.2331	
11		435.0212	37.72071	131.3788	
12		447.0939	51.1765	173.4313	
13		475.2634	42.31313	134.8952	
14		496.1895	39.47619	120.5434	
15		506.1303	32.08666	96.05451	
16		526.6776	37.13036	106.8169	
17		551.1386	19.1223	52.56962	
18		562.8799	24.11787	64.92005	
19		621.5905	20.6105	50.23886	
20	▶	10380	75	10.94762	Upper Marker

Region table for sample 2 : S4

From [s]	To [s]	Corr. Area	% of Total	Average Size [bp]	Size distribution in CV [%]	Conc. [pg/µl]	Molarity [pmol/l]	Color
60.51165	95.34863	533.4929	84.22347	442.8397	27.71867	940.6061	3644.166	

© Copyright 2003 - 2024 Agilent, Inc.

Assay Class: High Sensitivity DNA Assay
Data Path: C:\...gh Sensitivity DNA Assay_DE23101764_2024-11-07_11-35-32.xad
Created: 11/7/2024 11:35:31 AM
Modified: 11/7/2024 12:16:58 PM

Electropherogram Summary Continued ...

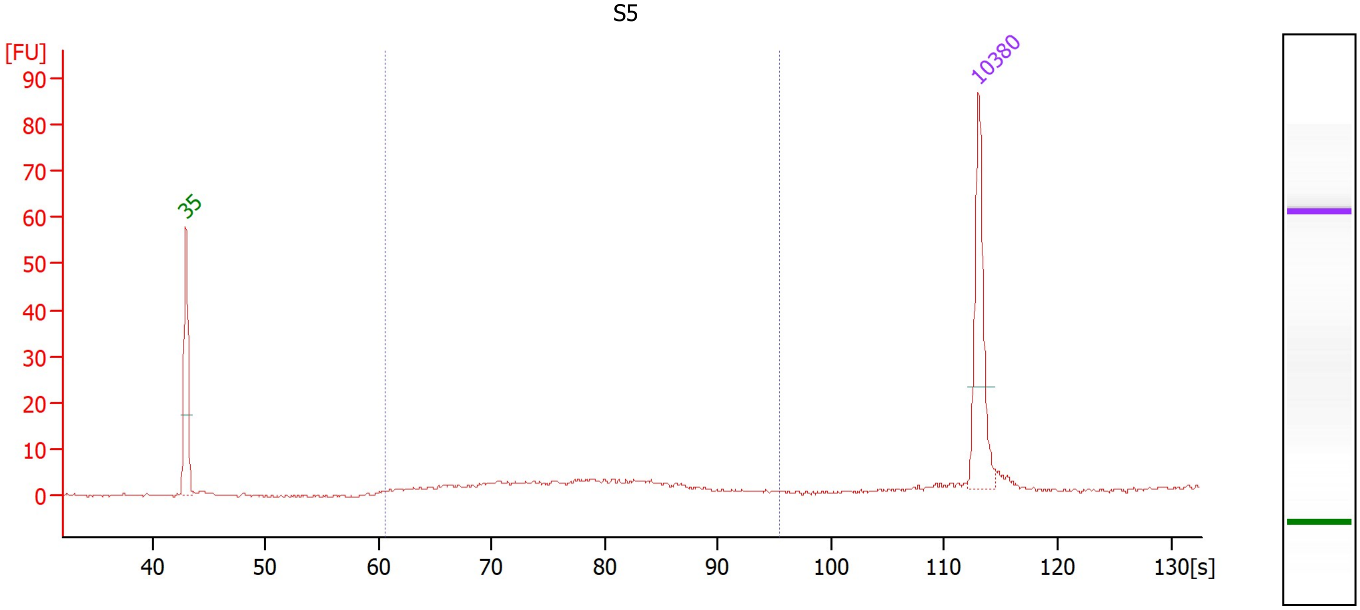

Overall Results for sample 3 : S5

Number of peaks found: 0
Corr. Area 1: 67.9
Noise: 0.3

Peak table for sample 3 : S5

	Peak	Size [bp]	Conc. [pg/µl]	Molarity [pmol/l]	Observations
1	◀	35	125	5411.25	Lower Marker
2	▶	10380	75	10.94762	Upper Marker

Region table for sample 3 : S5

From [s]	To [s]	Corr. Area	% of Total	Average Size [bp]	Size distribution in CV [%]	Conc. [pg/µl]	Molarity [pmol/l]	Color
60.51165	95.34863	67.86057	83.88619	394.8374	27.63266	111.7767	480.2101	

© Copyright 2003 - 2024 Agilent, Inc.

Assay Class: High Sensitivity DNA Assay
Data Path: C:\...gh Sensitivity DNA Assay_DE23101764_2024-11-07_11-35-32.xad

Created: 11/7/2024 11:35:31 AM
Modified: 11/7/2024 12:16:58 PM

Electropherogram Summary Continued ...

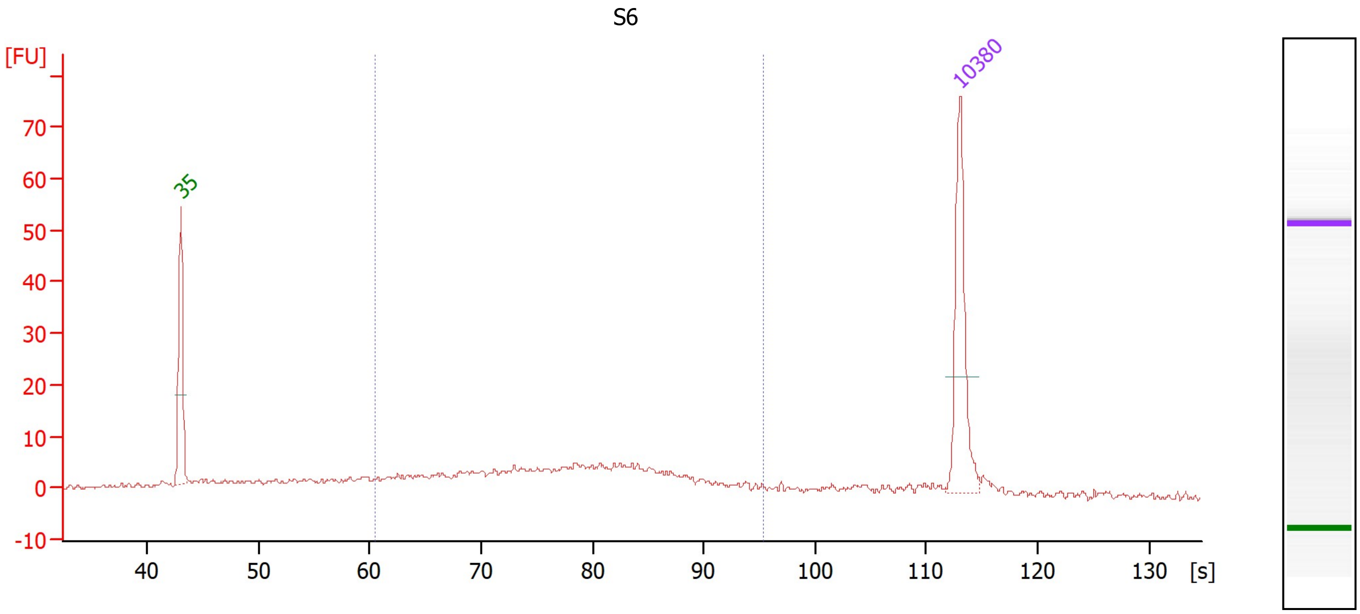

Overall Results for sample 4 : S6

Number of peaks found: 0
Corr. Area 1: 159.9
Noise: 0.5

Peak table for sample 4 : S6

	Peak	Size [bp]	Conc. [pg/µl]	Molarity [pmol/l]	Observations
1	◀	35	125	5411.25	Lower Marker
2	▶	10380	75	10.94762	Upper Marker

Region table for sample 4 : S6

From [s]	To [s]	Corr. Area	% of Total	Average Size [bp]	Size distribution in CV [%]	Conc. [pg/µl]	Molarity [pmol/l]	Color
60.51165	95.34863	159.9133	64.33517	430.2047	35.83505	256.7502	1076.385	

 © Copyright 2003 - 2024 Agilent, Inc.

Assay Class: High Sensitivity DNA Assay
Data Path: C:\...gh Sensitivity DNA Assay_DE23101764_2024-11-07_11-35-32.xad

Created: 11/7/2024 11:35:31 AM
Modified: 11/7/2024 12:16:58 PM

Electropherogram Summary Continued ...

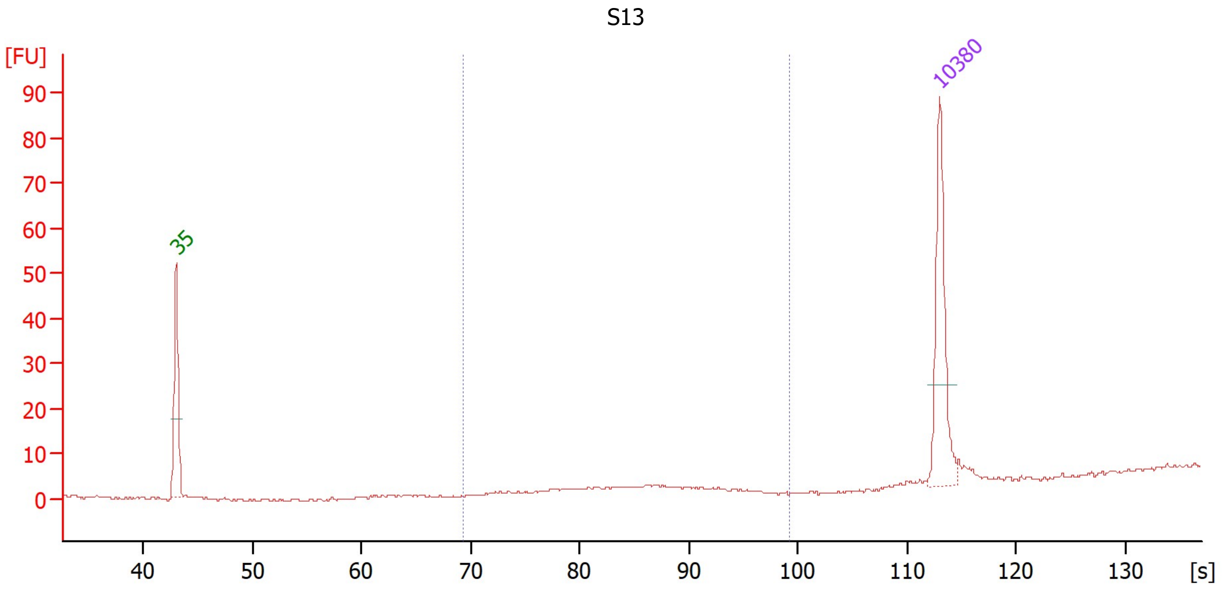

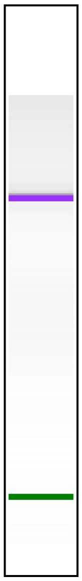

Overall Results for sample 5 : S13

Number of peaks found: 0

Corr. Area 1: 1.8

Noise: 0.2

Peak table for sample 5 : S13

	Peak	Size [bp]	Conc. [pg/µl]	Molarity [pmol/l]	Observations
1	◀	35	125	5411.25	Lower Marker
2	▶	10380	75	10.94762	Upper Marker

Region table for sample 5 : S13

From [s]	To [s]	Corr. Area	% of Total	Average Size [bp]	Size distribution in CV [%]	Conc. [pg/µl]	Molarity [pmol/l]	Color
69.38396	99.30679	1.757641	20.54807	507.8468	10.77235	2.464051	7.454307	

© Copyright 2003 - 2024 Agilent, Inc.

Assay Class: High Sensitivity DNA Assay
Data Path: C:\...gh Sensitivity DNA Assay_DE23101764_2024-11-07_11-35-32.xad
Created: 11/7/2024 11:35:31 AM
Modified: 11/7/2024 12:16:58 PM

Electropherogram Summary Continued ...

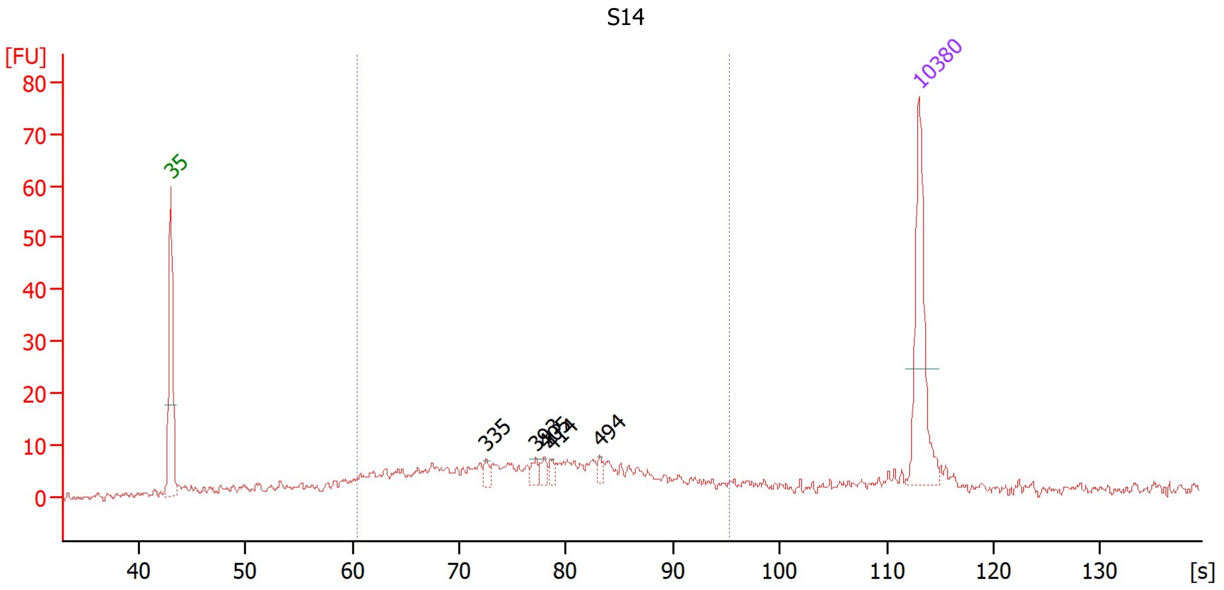

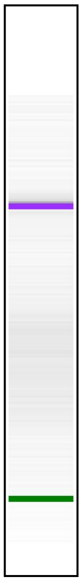

Overall Results for sample 6 : S14

Number of peaks found: 5 Corr. Area 1: 191.8

Noise: 0.6

Peak table for sample 6 : S14

	Peak	Size [bp]	Conc. [pg/µl]	Molarity [pmol/l]	Observations
1	◀	35	125	5411.25	Lower Marker
2		335.4643	7.71531	34.84678	
3		392.8427	7.817872	30.15265	
4		404.8069	7.286517	27.27267	
5		414.3782	6.436485	23.53463	
6		493.5605	5.747595	17.64418	
7	▶	10380	75	10.94762	Upper Marker

Region table for sample 6 : S14

From [s]	To [s]	Corr. Area	% of Total	Average Size [bp]	Size distribution in CV [%]	Conc. [pg/µl]	Molarity [pmol/l]	Color
60.51165	95.34863	191.8033	68.71842	406.617	36.19201	323.7516	1429.855	

 © Copyright 2003 - 2024 Agilent, Inc.

Assay Class: High Sensitivity DNA Assay
Data Path: C:\...gh Sensitivity DNA Assay_DE23101764_2024-11-07_11-35-32.xad

Created: 11/7/2024 11:35:31 AM
Modified: 11/7/2024 12:16:58 PM

Electropherogram Summary Continued ...

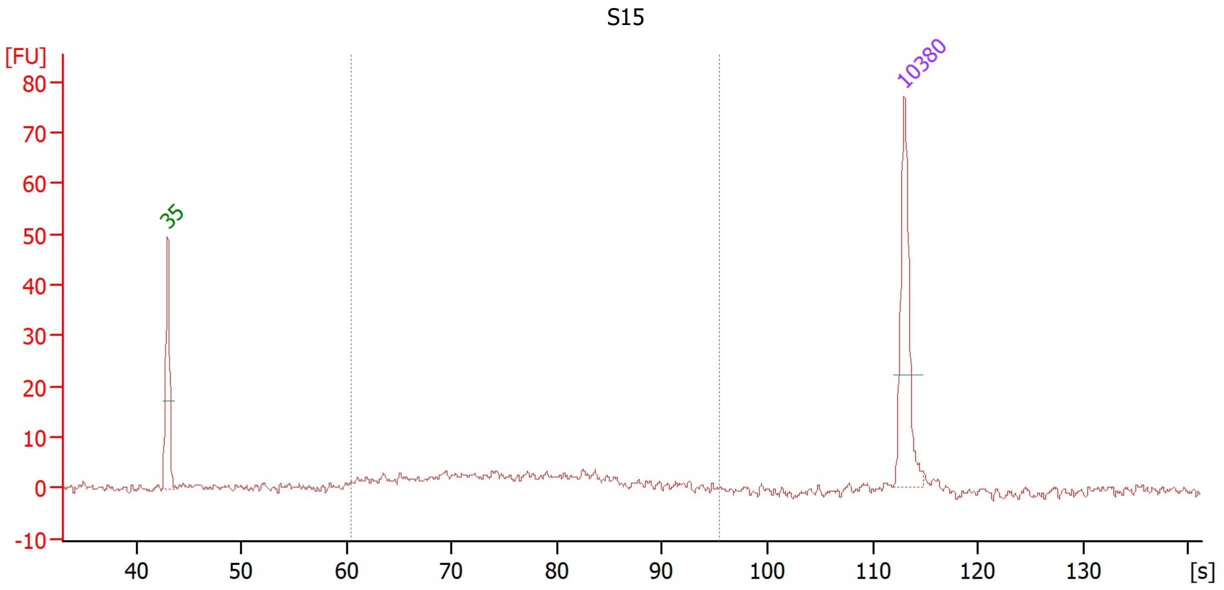

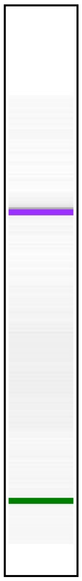

Overall Results for sample 7 : S15

Number of peaks found:	0	Corr. Area 1:	85.5
Noise:	0.8		

Peak table for sample 7 : S15

	Peak	Size [bp]	Conc. [pg/µl]	Molarity [pmol/l]	Observations
1	◀	35	125	5411.25	Lower Marker
2	▶	10380	75	10.94762	Upper Marker

Region table for sample 7 : S15

From [s]	To [s]	Corr. Area	% of Total	Average Size [bp]	Size distribution in CV [%]	Conc. [pg/µl]	Molarity [pmol/l]	Color
60.51165	95.34863	85.54471	78.95391	388.9453	33.85117	151.3376	682.4999	

 © Copyright 2003 - 2024 Agilent, Inc.

Assay Class: High Sensitivity DNA Assay
Data Path: C:\...gh Sensitivity DNA Assay_DE23101764_2024-11-07_11-35-32.xad
Created: 11/7/2024 11:35:31 AM
Modified: 11/7/2024 12:16:58 PM

Electropherogram Summary Continued ...

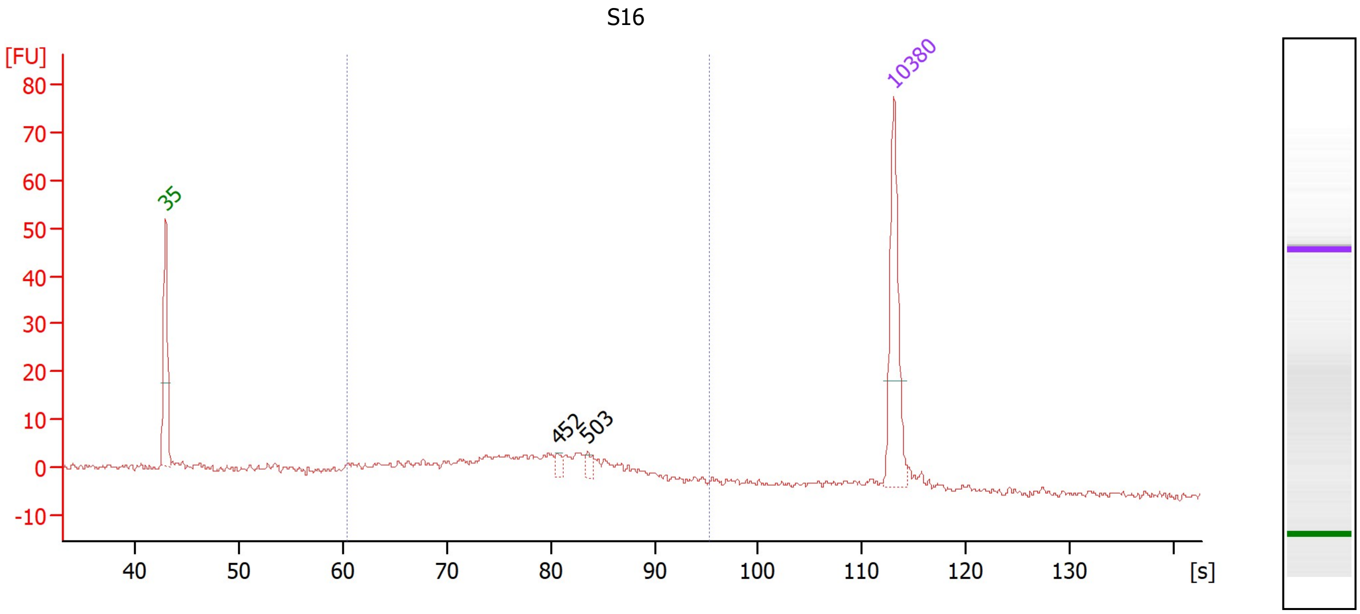

Overall Results for sample 8 : S16

Number of peaks found: 2
Corr. Area 1: 116.8
Noise: 0.7

Peak table for sample 8 : S16

	Peak	Size [bp]	Conc. [pg/µl]	Molarity [pmol/l]	Observations
1	◀	35	125	5411.25	Lower Marker
2		451.7235	6.891012	23.11351	
3		503.2512	6.511692	19.6049	
4	▶	10380	75	10.94762	Upper Marker

Region table for sample 8 : S16

From [s]	To [s]	Corr. Area	% of Total	Average Size [bp]	Size distribution in CV [%]	Conc. [pg/µl]	Molarity [pmol/l]	Color
60.51165	95.34863	116.849	85.02299	418.3803	29.29969	188.9216	778.374	

© Copyright 2003 - 2024 Agilent, Inc.

Assay Class: High Sensitivity DNA Assay
Data Path: C:\...gh Sensitivity DNA Assay_DE23101764_2024-11-07_11-35-32.xad

Created: 11/7/2024 11:35:31 AM
Modified: 11/7/2024 12:16:58 PM

Electropherogram Summary Continued ...

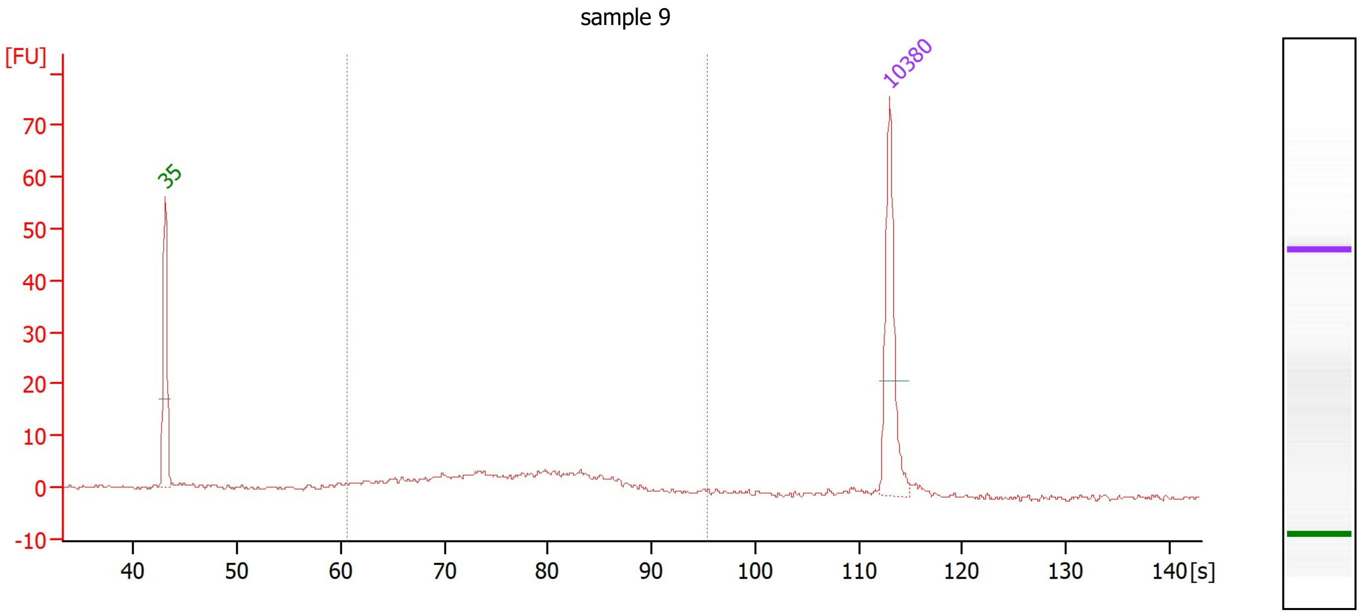

Overall Results for sample 9 : sample 9

Number of peaks found: 0
Corr. Area 1: 106.1
Noise: 0.3

Peak table for sample 9 : sample 9

	Peak	Size [bp]	Conc. [pg/µl]	Molarity [pmol/l]	Observations
1	◀	35	125	5411.25	Lower Marker
2	▶	10380	75	10.94762	Upper Marker

Region table for sample 9 : sample 9

From [s]	To [s]	Corr. Area	% of Total	Average Size [bp]	Size distribution in CV [%]	Conc. [pg/µl]	Molarity [pmol/l]	Color
60.51165	95.34863	106.0851	77.19463	404.6603	31.67827	180.1603	773.5215	

 © Copyright 2003 - 2024 Agilent, Inc.

Assay Class: High Sensitivity DNA Assay
Data Path: C:\...gh Sensitivity DNA Assay_DE23101764_2024-11-07_11-35-32.xad

Created: 11/7/2024 11:35:31 AM
Modified: 11/7/2024 12:16:58 PM

Electropherogram Summary Continued ...

sample 10

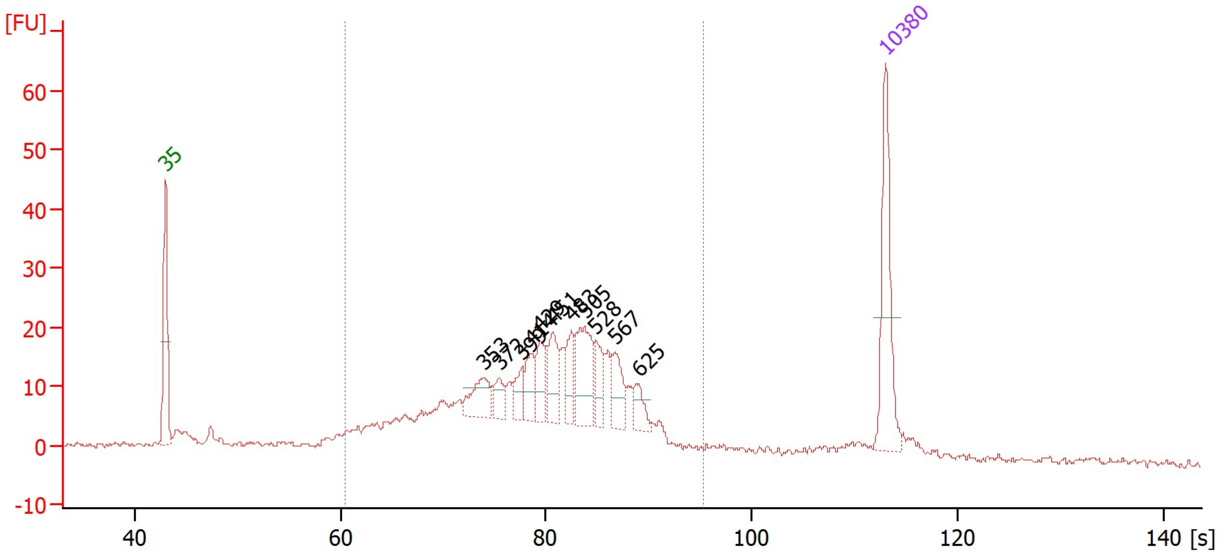

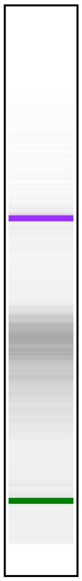

Overall Results for sample 10 : sample 10

Number of peaks found:	11	Corr. Area 1:	447.9
Noise:	0.5		

Peak table for sample 10 : sample 10

	Peak	Size [bp]	Conc. [pg/µl]	Molarity [pmol/l]	Observations
1	◀	35	125	5411.25	Lower Marker
2		352.8998	37.76183	162.1277	
3		371.6879	18.56474	75.67735	
4		398.8982	19.92756	75.69162	
5		413.8287	25.80498	94.47973	
6		429.1945	32.26575	113.9051	
7		450.8876	37.74818	126.8479	
8		482.5233	29.89996	93.88754	
9		505.1256	64.88491	194.6256	
10		528.201	27.75711	79.62154	
11		566.6602	31.45761	84.11213	
12		625.2763	16.23565	39.34171	
13	▶	10380	75	10.94762	Upper Marker

Region table for sample 10 : sample 10

From [s]	To [s]	Corr. Area	% of Total	Average Size [bp]	Size distribution in CV [%]	Conc. [pg/µl]	Molarity [pmol/l]	Color
60.51165	95.34863	447.8918	82.89736	445.8702	28.36453	884.5687	3419.955	

 © Copyright 2003 - 2024 Agilent, Inc.

Assay Class: High Sensitivity DNA Assay
Data Path: C:\...gh Sensitivity DNA Assay_DE23101764_2024-11-07_11-35-32.xad

Created: 11/7/2024 11:35:31 AM
Modified: 11/7/2024 12:16:58 PM

Electropherogram Summary Continued ...

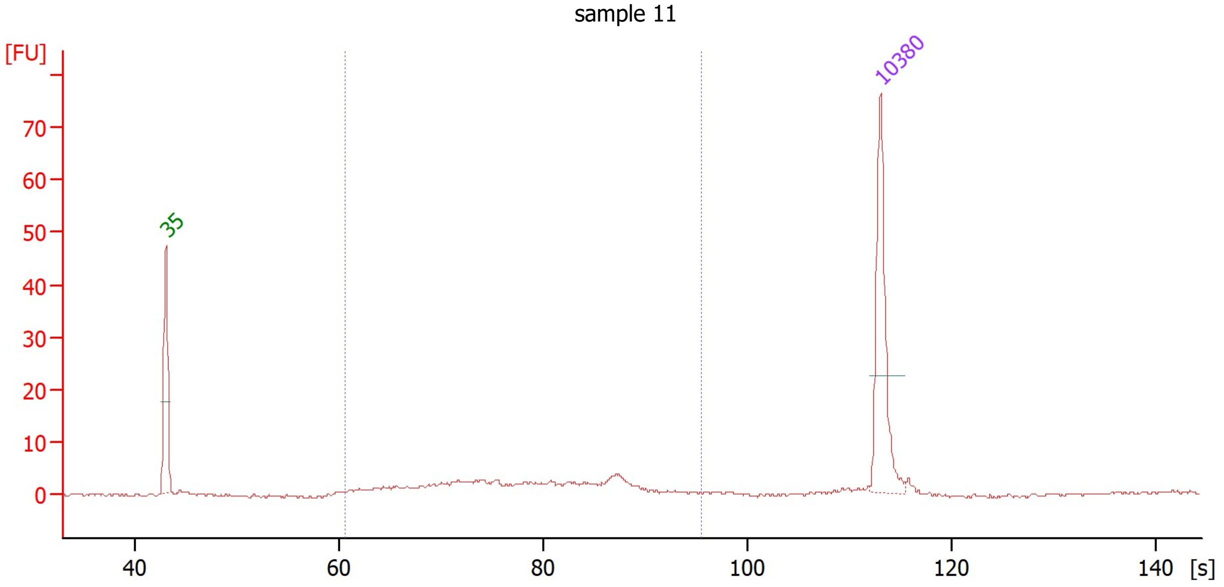

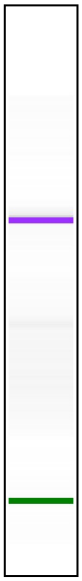

Overall Results for sample 11 : sample 11

Number of peaks found: 0
Corr. Area 1: 69.9
Noise: 0.2

Peak table for sample 11 : sample 11

	Peak	Size [bp]	Conc. [pg/µl]	Molarity [pmol/l]	Observations
1	◀	35	125	5411.25	Lower Marker
2	▶	10380	75	10.94762	Upper Marker

Region table for sample 11 : sample 11

From [s]	To [s]	Corr. Area	% of Total	Average Size [bp]	Size distribution in CV [%]	Conc. [pg/µl]	Molarity [pmol/l]	Color
60.51165	95.34863	69.85625	89.58527	421.0891	31.55183	111.6007	464.2974	

© Copyright 2003 - 2024 Agilent, Inc.

Assay Class: High Sensitivity DNA Assay
Data Path: C:\...gh Sensitivity DNA Assay_DE23101764_2024-11-07_11-35-32.xad
Created: 11/7/2024 11:35:31 AM
Modified: 11/7/2024 12:16:58 PM

Gel Image

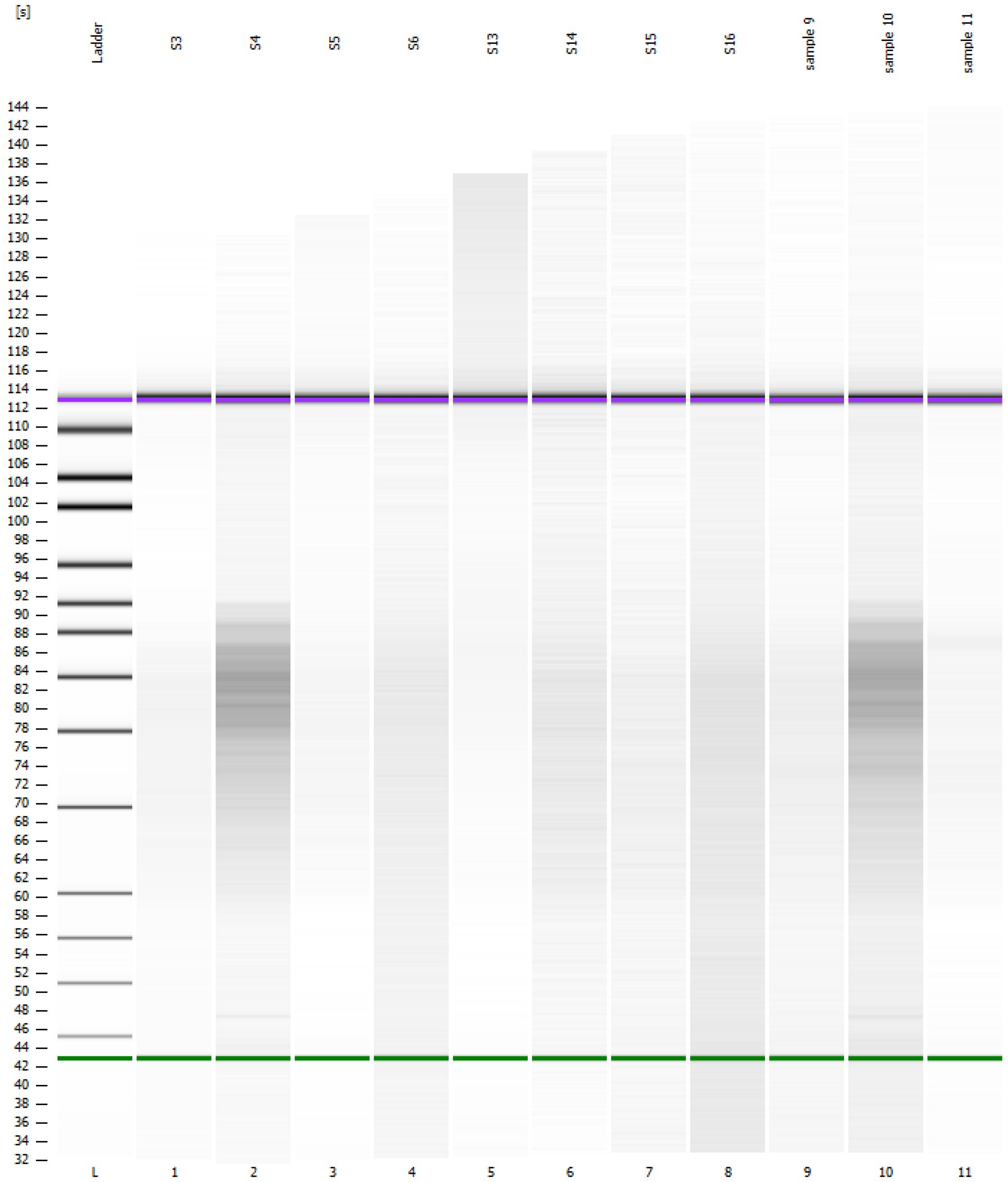

 © Copyright 2003 - 2024 Agilent, Inc.
